# mRNA Profiling Reveals Determinants of Trastuzumab Efficiency in HER2-Positive Breast Cancer

**DOI:** 10.1371/journal.pone.0117818

**Published:** 2015-02-24

**Authors:** Silvia von der Heyde, Steve Wagner, Alexander Czerny, Manuel Nietert, Fabian Ludewig, Gabriela Salinas-Riester, Dorit Arlt, Tim Beißbarth

**Affiliations:** 1 Statistical Bioinformatics, Department of Medical Statistics, University Medical Center Göttingen, Göttingen, Germany; 2 IndivuTest GmbH, Hamburg, Germany; 3 Division of Stem Cells and Cancer, German Cancer Research Center, Heidelberg, Germany; 4 Division of Molecular Genome Analysis, German Cancer Research Center, Heidelberg, Germany; 5 DNA Microarray and Deep-Sequencing Facility Göttingen, Department of Developmental Biochemistry, University of Göttingen, Göttingen, Germany; University of South Alabama, UNITED STATES

## Abstract

Intrinsic and acquired resistance to the monoclonal antibody drug trastuzumab is a major problem in the treatment of HER2-positive breast cancer. A deeper understanding of the underlying mechanisms could help to develop new agents. Our intention was to detect genes and single nucleotide polymorphisms (SNPs) affecting trastuzumab efficiency in cell culture. Three HER2-positive breast cancer cell lines with different resistance phenotypes were analyzed. We chose BT474 as model of trastuzumab sensitivity, HCC1954 as model of intrinsic resistance, and BTR50, derived from BT474, as model of acquired resistance. Based on RNA-Seq data, we performed differential expression analyses on these cell lines with and without trastuzumab treatment. Differentially expressed genes between the resistant cell lines and BT474 are expected to contribute to resistance. Differentially expressed genes between untreated and trastuzumab treated BT474 are expected to contribute to drug efficacy. To exclude false positives from the candidate gene set, we removed genes that were also differentially expressed between untreated and trastuzumab treated BTR50. We further searched for SNPs in the untreated cell lines which could contribute to trastuzumab resistance. The analysis resulted in 54 differentially expressed candidate genes that might be connected to trastuzumab efficiency. 90% of 40 selected candidates were validated by RT-qPCR. ALPP, CALCOCO1, CAV1, CYP1A2 and IGFBP3 were significantly higher expressed in the trastuzumab treated than in the untreated BT474 cell line. GDF15, IL8, LCN2, PTGS2 and 20 other genes were significantly higher expressed in HCC1954 than in BT474, while NCAM2, COLEC12, AFF3, TFF3, NRCAM, GREB1 and TFF1 were significantly lower expressed. Additionally, we inferred SNPs in HCC1954 for CAV1, PTGS2, IL8 and IGFBP3. The latter also had a variation in BTR50. 20% of the validated subset have already been mentioned in literature. For half of them we called and analyzed SNPs. These results contribute to a better understanding of trastuzumab action and resistance mechanisms.

## Introduction

The ‘HER2-positive’ subtype of breast cancer overexpresses the human epidermal growth factor receptor 2 (HER2). This receptor tyrosine kinase is part of the epidermal growth factor receptor (EGFR) family, further including HER1 (EGFR), HER3 and HER4 [1]. It is overexpressed in 10–20% of breast tumors, and the related subtype is associated with increased recurrence and mortality rates [2, 3].

The humanized monoclonal antibody trastuzumab targets specifically the extracellular domain of HER2 and is part of the adjuvant treatment of patients with HER2-positive (HER2+) early breast cancer [4]. The improved outcome by adding trastuzumab to chemotherapy for example is not completely understood. So far it has been associated with different mechanisms of action apart from inhibiting HER2, its dimerization and cleavage [4, 5]. These mechanisms include inhibition of downstream signal transduction pathways like the PI3K pathway, antigen-dependent cellular cytotoxicity (ADCC), induction of cell cycle arrest and apoptosis or inhibition of tumor angiogenesis.

Although trastuzumab provides clinical benefit to women with HER2+ breast cancer, not all patients respond [6]. Primary or acquired resistance limits the success of trastuzumab. Diverse possible mechanisms have been discussed [4, 5]. Among others, these include increased HER2, HER1 or HER3 expression, steric hindrance of HER2-antibody interaction, constitutive activation of the PI3K pathway due to mutations in the PIK3CA gene or loss of PTEN, alternative cell signaling induced by EGFR family members, MET receptor or insulin-like growth factor 1 receptor (IGF-IR), and overexpression of transforming growth factor (TGF)-*α*, neuregulin or vascular endothelial growth factor (VEGF). Corresponding therapeutic strategies to overcome or avoid resistance to trastuzumab have been developed, and several new agents are in clinical development. Studies in metastatic disease led to the approval of new HER2-targeted therapies using small molecule tyrosine kinase inhibitors such as lapatinib and HER2/HER3 antibodies such as pertuzumab [7]. However, so far it is not possible to predict prior to trastuzumab treatment which patients will develop resistance. A need for a better understanding of the mechanisms of trastuzumab action and resistance persists.

This study aims at detecting genes and single nucleotide polymorphisms (SNPs) affecting trastuzumab efficiency in three cell lines with different resistance phenotypes. These include trastuzumab sensitivity, intrinsic resistance due to a mutated PIK3CA gene, and acquired resistance. Ten percent of the candidate genes inferred via mRNA profiling have already been supported by literature. The remaining ones, partially known to be involved in breast cancer, could also contribute to novel strategies preventing trastuzumab resistance.

SNPs are the most common genetic variations and can be associated with heritable phenotypes. Related data is deposited in public databases [8]. SNPs are defined as single base pair positions in genomic DNA at which different sequence alternatives (alleles) exist in normal (non-diseased) individuals in some population(s), wherein the least frequent allele has an abundance of at least 1% [9]. According to this definition, Brookes concluded that single base insertion/deletion variants (indels) would not formally be considered as SNPs. However, he stated that in practice the term SNP is used rather loosely. For example, single base variants in cDNAs (cSNPs) are usually called SNPs, since most of them reflect underlying genomic DNA variants. This could be misleading in case of disease predisposing single base variants, which occur in some non-diseased individuals. Also Brookes warned that the ‘some population’ component of the definition is limited by practical challenges of attaining representative global population samples. He summarized that the term ‘SNP’ is being widely used as a label for many different types of subtle sequence variation. Being aware of the definition mentioned before, we decided to call our detected sequence variations, which are potentially related to trastuzumab efficacy, ‘SNPs’ in the following.

SNPs may unravel multifactorial diseases such as most cancers or drug response, bearing pharmacogenetic potential in the context of personalized medicine. Direct DNA sequencing is the favored high-throughput method for SNP identification [10, 11]. However, Quinn et al. evaluated the performance of different SNP calling methods by applying them to RNA-Seq data and comparing results with sequence variation data from *1000 Genomes* [12]. They regard RNA-Seq SNP data as a useful by-product of sequence-based transcriptome analysis. According to their results, one can detect a high proportion of mutations of expressed genes via RNA-Seq. We intended to reveal SNPs in our candidate genes which might determine trastuzumab efficiency in the untreated cell lines. Above that we analyzed SNPs that affect cell signaling in the MAPK and PI3K pathway. These variations could be responsible for the different resistance cell phenotypes. Hence, the combination of differential gene expression and SNP analysis could help to predict the efficacy of trastuzumab therapy. Consequently, the detection of corresponding genes and their variations could contribute to an improved patient stratification.

## Materials and Methods

### Cell lines

Three human HER2-amplified breast cancer cell lines (BT474, HCC1954 and BTR50) were chosen as model systems of trastuzumab sensitivity (BT474 [6]), intrinsic (HCC1954 [6, 13]) and acquired (BTR50) resistance. While HCC1954 is known to be trastuzumab resistant due to a hotspot PIK3CA mutation (H1047R, PI3K gain-of-function), BT474 exhibits PIK3CA wild type behavior [14]. The cell line BTR50 is a trastuzumab-conditioned version of BT474, as explained in the corresponding subsection.

The cells were grown in a monolayer and collected as a cell pellet after trypsin treatment. RNA was harvested from cell pellet using the miRNeasy kit (Qiagen).

#### BT474

The human breast cancer cell line BT474 was directly obtained from the American Type Culture Collection (ATCC), catalogue no. HTB-20. It was cultured in Dulbecco’s Modified Eagle Medium (DMEM) supplemented with 10% fetal bovine serum, 0.01 mg/ml of insulin and 1% penicillin/streptomycin. The cells were cultured at 37°C in an atmosphere containing 5% CO_2_. Cells were harvested with trypsin-ethylenediamine tetraacetic acid (EDTA) (0.5 g/L trypsin; 0.2 g/L EDTA; Sigma). The cells were split three times per week.

#### HCC1954

The human breast cancer cell line HCC1954 was directly obtained from ATCC, catalogue no. CRL-2338, and cultured in RPMI media (Gibco) supplemented with 10% fetal bovine serum (Gibco). The medium was supplemented with 1% penicillin/streptomycin (Gibco). The cells were cultured at 37°C in an atmosphere containing 5% CO_2_. Cells were harvested with EDTA (0.5 g/L trypsin; 0.2 g/L EDTA; Sigma). The cells were split three times per week.

#### BTR50

**Fig 1:**
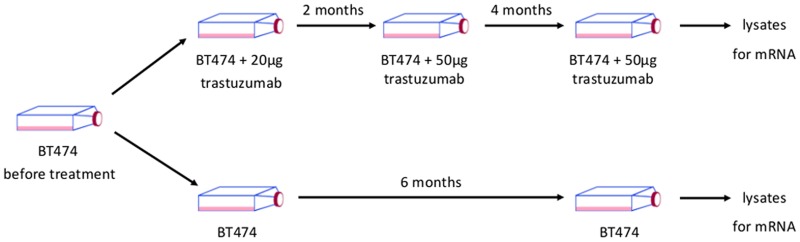
Development of BTR50 cells. Trastuzumab resistant cells were developed by culturing parental BT474 cells in the presence of 20/50 *μ*g trastuzumab for around 6 months.

**Fig 2:**
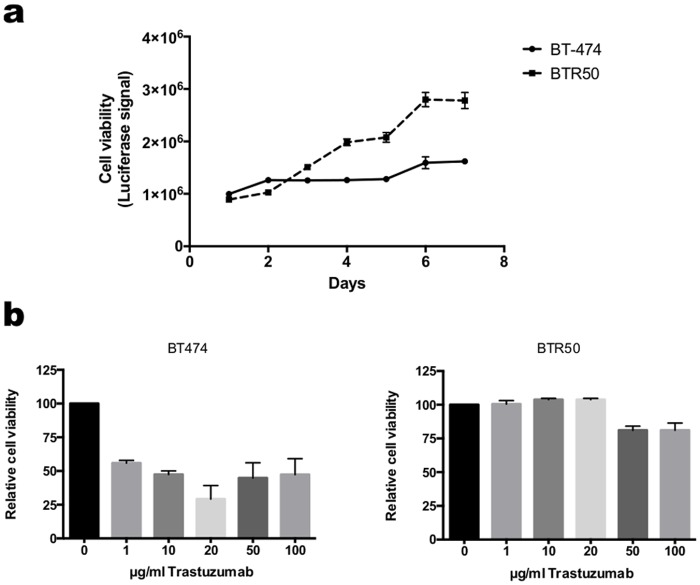
BT474 cells have acquired resistance to trastuzumab. (a) Proliferation rate of BT474 (parental) and trastuzumab resistant BT474 (BTR50) cells treated with 20 *μ*g/ml trastuzumab. Proliferation rates were measured daily over 7 days by a luciferase-based viability assay. (b) Sensitivity of BT474 (parental) and trastuzumab resistant BTR50 cells towards increasing concentrations of trastuzumab. Cell viability was determined by a luciferase-based viability assay after 7 days of treatment.

Resistant cells (BTR) were developed by culturing the epithelial BT474 breast cancer cells (wild type, wt) in the presence of 50 *μ*g trastuzumab (Roche) for around six months. Parental cells (wt) were cultured in parallel to resistant ones without the addition of trastuzumab (Fig. 1). Resistance of the cells to trastuzumab was verified by cell viability assays (Fig. 2). Trastuzumab markedly reduced the growth of BT474 (wt) cells compared to trastuzumab resistant BT474 (BTR50) cells.

#### Cell viability assay and determination of trastuzumab sensitivity

For the measurement of cell viability, both parental BT474 and trastuzumab resistant BTR50 cells were seeded in 96 well plates. Cell viability was determined every day for in total 7 days using Cell Titer Glo Luminescent Cell Viability Assay (Promega) following the manufacturer’s instructions (Fig. 2a). The sensitivity towards trastuzumab was defined by treatment of both cell lines with increasing concentrations of trastuzumab and a subsequent measurement of cell viability after 7 days (Fig. 2b).

#### Trastuzumab (Herceptin) treatment

For treatment experiments, 2×10^5^ cells were seeded in T25 flasks and cultivated as described before. Cells were treated with 20 *μ*g/ml trastuzumab (Roche Diagnostics GmbH, Penzberg, Germany) or grown in full growth media without inhibitors. Cell pellets were harvested after 72 h. The cells were incubated in the standard media 24 h before addition of trastuzumab or fresh full growth media.

### RNA sequencing

Total RNA was isolated from the cell lines BT474, HCC1954 and BTR50 using the Trizol (Invitrogen) method according to the manufacturer’s recommendations. Afterwards, the samples were DNAse I (Sigma) treated in order to remove DNA contamination. RNA quality was determined using the Agilent 2100 Bioanalyzer (Agilent Technologies, Santa Clara, CA, USA) microfluidic electrophoresis. Only samples with comparable RNA integrity numbers were selected for deep sequencing.

Library preparation for RNA-Seq was performed using the TruSeq RNA Sample Preparation Kit (Illumina, catalog ID RS-122-2002) starting from 800 ng of total RNA. Accurate quantitation of cDNA libraries was performed by using the QuantiFluor™dsDNA System (Promega). The size range of final cDNA libraries was determined applying the DNA 1000 chip on the Bioanalyzer 2100 from Agilent (280 bp). cDNA libraries were amplified and sequenced by using the cBot and HiSeq 2000 from Illumina (SR, 1×51 bp, 6 GB ca. 30–35 million reads per sample).

Sequence images were transformed with Illumina software BaseCaller to bcl files, which were demultiplexed to fastq files with CASAVA (version 1.8.2). Quality check was done via FastQC (version 0.10.1, Babraham Bioinformatics).

The sequenced reads were hard (first five bases) and soft (last bases) trimmed as well as trimmed for adapter sequences via Flexbar [15] (version 2.32). Afterwards they were mapped to the human reference genome (GRCh37, Gencode [16] release 14) using STAR [17] (version 2.3.0), allowing maximal three mismatches. Conversion of SAM to BAM files and corresponding sorting was done via SAMtools [18] (version 0.1.18). Counting the reads to each gene to the UCSC gtf gene annotation file (March 2012) was done via HTSeq [19] (0.5.3p9, htseq-count).

### SNP calling

The reads were aligned against the Ensembl [20] reference genome release 71 (GRCh37) with STAR (version 2.3.0), allowing for 5% mismatches of total read length. STAR was used with additional splice junction annotation. Read group definition as well as removal of duplicates after the alignment step was done via Picard command line tools (release 1.99, http://picard.sourceforge.net). For calling variants from RNA-Seq data, the Genome Analysis Toolkit (GATK) [21] (version 2.7.2) standard SNP best-practice protocol was used with the additional option -U ALLOW_N_CIGAR_READS. Standard hard filtering was applied. Read quality was reassigned from 255 to 60. Low quality reads were neglected. Just SNPs with a read depth ≥ 10 were selected.

For the analysis of the derived SNP candidates by applying GATK we used the Variant Effect Predictor (VEP) [22].

### Differential expression analysis

Normalization of read counts to the library size, estimation of dispersions (method = ‘blind’, sharingMode = ‘fit-only’) and testing for differentially expressed (DE) genes based on a statistical test assuming negative binomial data distribution was computed via the DESeq [23] (version 1.12.1) R [24] package. Just genes exceeding 20 counts for at least one sample were kept for further analysis. The numerator and denominator of fold changes (FC) were increased by one to account for zero values. Significant genes were filtered to a minimum of 2xFC and fdr < 0.05 with multiple testing correction according to Benjamini and Hochberg [25].

Based on RNA-Seq data, we performed DE analyses on six samples, i.e. the breast cancer cell lines BT474, HCC1954 and BTR50 with and without trastuzumab treatment. In detail, five separate two-sample tests were performed and normalization was done per sample pair of consideration. First we tested for DE between resistant and wild type cells, i.e. HCC1954 and BTR50 vs. BT474, respectively. This revealed 46 significant genes which might contribute to resistance. Next we tested for DE between untreated and trastuzumab treated cells, i.e. each of the three cell lines vs. its trastuzumab treated version. The test for BT474 revealed 18 significant genes which might contribute to trastuzumab efficiency. To exclude false positives from the combined set of 64 genes, we removed ten genes that were also significant in the test for BTR50. No trastuzumab effect was expected for the resistant cell line. The same would have held for HCC1954, but the related test revealed no significant genes overlapping with our candidate set. This way we discovered 54 genes that might determine trastuzumab efficiency in HER2+ breast cancer cell lines. Annotation and functional association of the candidate genes to biological processes (BP, Gene Ontology annotation) was added via biomaRt [26] (version 2.16.0). The raw and normalized data have been submitted to the Gene Expression Omnibus (GEO) with accession number GSE55005.

### Real-time quantitative PCR

Out of the 54 candidate genes detected by mRNA profiling we chose 40 for validation via real-time quantitative PCR (RT-qPCR). mRNA purification was performed at 4°C using the miRNeasy Kit (Qiagen, Hilden, Germany) according to the manufactures recommendation. cDNA was generated using the High Capacity cDNA Reverse Transcription kit (Applied Biosystems, Darmstadt, Germany) from total RNA isolated from breast cancer cell lines. Primer combinations for the respective genes were designed according to the Harvard Primer Bank (http://pga.mgh.harvard.edu/primerbank) and are listed in S1 Table. 10 ng of each cDNA were used per approach. The final concentrations were 1x Applied Biosystems Power SYBR Green Master Mix (Applied Biosystems, Darmstadt, Germany) and 10 *μ*M of each primer. RT-qPCR was performed in a total volume of 12*μ*l. Glycerinaldehyd-3-phosphat-Dehydrogenase (GAPDH) was used as reference gene. The ViiA 7 Real-Time PCR System (Applied Biosystems, Darmstadt, Germany) was used for RT-qPCR analysis. Data analysis was performed as described in Livak and Schmittgen [27] with two biological replicates. The corresponding data are listed in S2 Table.

## Results and Discussion

### mRNA profiling reveals genes associated with trastuzumab efficiency

The differential expression (DE) revealed by RNA-Seq analysis was confirmed via RT-qPCR for 36 out of 40 candidate genes. The whole set consisted of 54 genes (S3 Table). The selection criterion was mainly based on literature support for association of genes with trastuzumab efficiency or breast cancer.

Six of the candidate genes were selected from the gene set inferred by testing DE between BT474 and its trastuzumab treated version. The whole subset consisted of eight genes. For the selected ones (ALPP, CALCOCO1, CAV1, CYP1A2, IGFBP3, L1CAM) a significantly higher expression after trastuzumab treatment of BT474 was validated apart from L1CAM.

Of the gene set inferred by testing DE between BT474 and HCC1954, 33 candidate genes were selected. The whole subset consisted of 45 genes. For all of the selected ones (AFF3, AKR1C1, CES1, CLDN1, COLEC12, CTGF, FXYD5, GBP1, GDF15, GREB1, IFI16, IFI27, IFITM1, IL8, KLK5, KLK6, KLK8, KRT17, KRT5, KRT81, LCN2, LIF, MYEOV, NCAM2, NRCAM, PGR, PTGS2, PTRF, S100A9, TFF1, TFF3, TGM2 and TINAGL1) the expected DE was validated apart from PGR and IFI16. AFF3, COLEC12, GREB1, NCAM2, NRCAM, TFF1 and TFF3 were significantly lower expressed in HCC1954 than in BT474, while the remaining ones were significantly higher expressed.

One candidate gene (MALAT1) was inferred by testing DE between BT474 and BTR50. Its significantly higher expression in BT474 was not validated.

#### Upregulated genes in BT474 upon trastuzumab treatment—indicators for drug sensitivity

**Fig 3:**
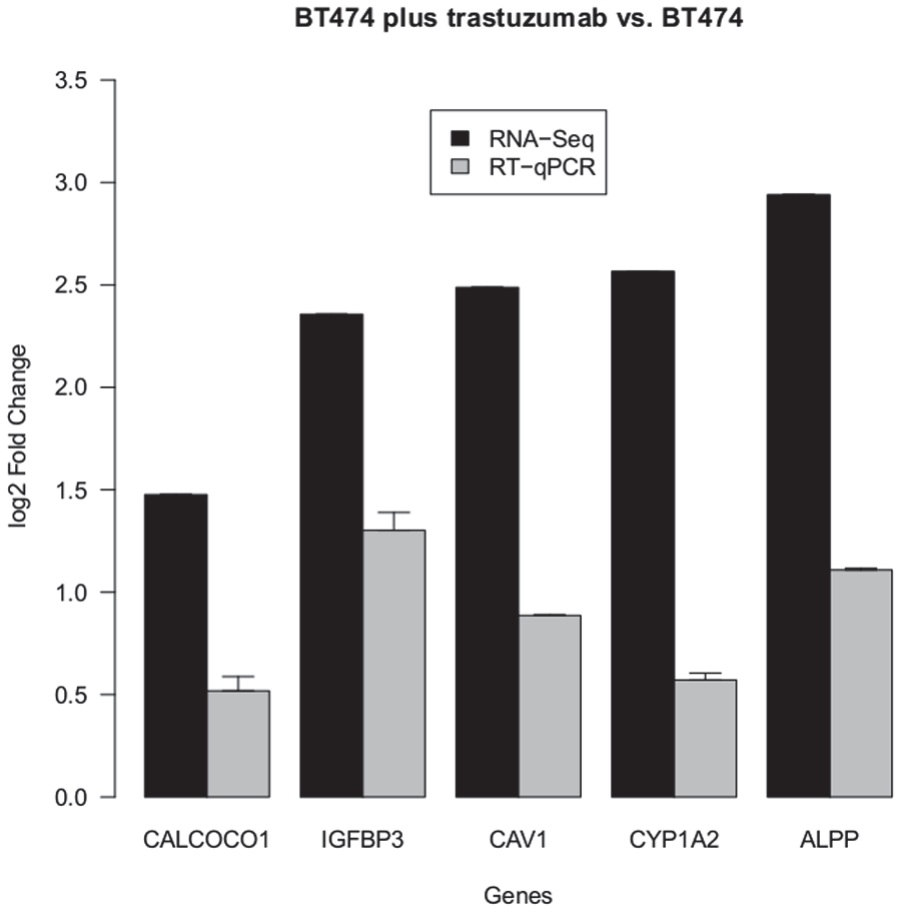
Fold Changes of DE genes (BT474 plus trastuzumab vs. BT474). The barchart displays the log2 fold changes of validated candidate genes, which significantly changed their expression in BT474 after trastuzumab treatment. The positive values indicate an upregulation upon drug treatment. Black bars denote values resulting from RNA-Seq analysis. Gray bars denote values resulting from RT-qPCR analysis.

The validated candidate genes ALPP, CYP1A2, CAV1, IGFBP3 and CALCOCO1 had positive log2 fold changes (FC) indicating an upregulation in BT474 when treated with trastuzumab. Fig. 3 shows the log2 FC resulting from RNA-Seq and RT-qPCR analysis, respectively. Table 1 lists the gene descriptions as well as the log2 FC and fdr of the RNA-Seq analysis. It is an excerpt of S3 Table.

**Table 1:** Upregulated genes in BT474 upon trastuzumab treatment.

HGNC Symbol	Description	Log2 FC	FDR
ALPP	alkaline phosphatase placental	2.94	1.7e-04
CALCOCO1	calcium binding and coiled-coil domain 1	1.48	3.0e-02
CAV1	caveolin 1 caveolae protein 22kDa	2.49	2.7e-02
CYP1A2	cytochrome P450 family 1 subfamily A polypeptide 2	2.57	8.1e-03
IGFBP3	insulin-like growth factor binding protein 3	2.36	5.9e-08

The table displays the results of the RNA-Seq data comparison between BT474 and its trastuzumab treated version. It holds the description, log2 fold change (FC) and fdr of the differentially expressed genes. The positive log2 fold changes indicate upregulated gene expression after trastuzumab treatment.

ALPP and CYP1A2 showed the highest FC, but no association with trastuzumab has been reported so far. ALPP encodes placental alkaline phosphatase (PLAP) which is known as a tumor marker in seminoma and ovarian cancer [28]. CYP1A2 (cytochrome P450, family 1, subfamily A, polypeptide 2), which activity is known to be modulated by specific polymorphisms, is already supposed to influence breast cancer, as it is involved in breast carcinogen activation on the one hand, but produces beneficial estrogen and anti-inflammatory acids on the other hand [29].

**Fig 4:**
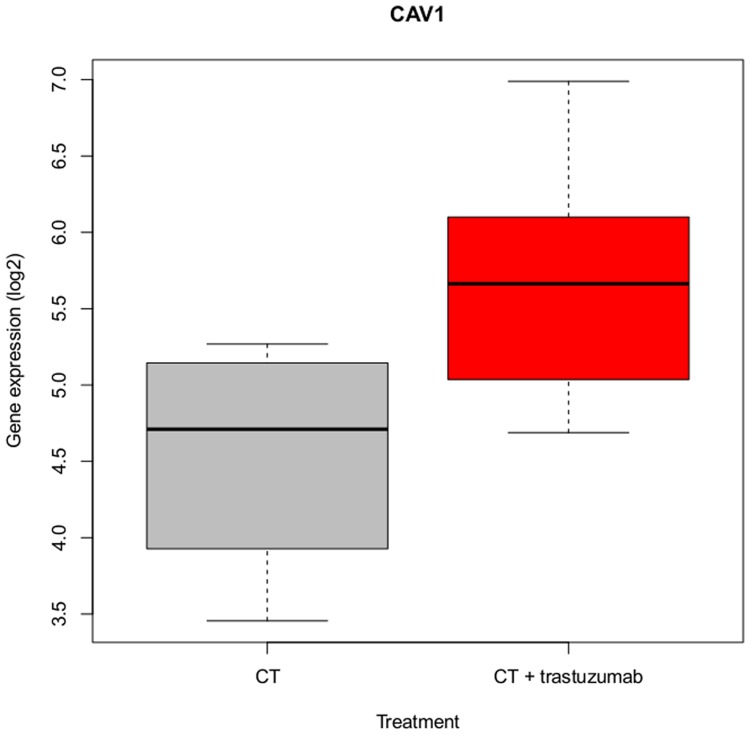
CAV1 expression is raised upon trastuzumab treatment. The boxplot displays CAV1 gene expression (log2) of seven patients treated with trastuzumab for one year in addition to neoadjuvant chemotherapy (red) and four patients treated with neoadjuvant chemotherapy only (gray). The patient data samples were selected from the transNOAH breast cancer trial (GEO series GSE50948).

IGFBP3 and CAV1 have already been reported in the context of trastuzumab efficacy. Regarding CAV1 (caveolin 1), Sekhar et al. discovered that although its tumor suppressor efficacy may be related to a HER2 downregulation in breast cancer cells, CAV1 and caveolae deficiency might be preferable under trastuzumab treatment [30]. Based on observations in the HER2+ human breast cancer cell line SKBR3, in which CAV1 was stably transduced, they speculated that an attenuated ADCC effect might be contributing to trastuzumab resistance. They found trastuzumab to be internalized and co-localized with CAV1, mediating endocytosis of HER2 by CAV1. Interestingly, in our study CAV1 was overexpressed upon trastuzumab treatment in the sensitive cell line BT474. The RT-qPCR data further revealed a stronger upregulation of CAV1 in HCC1954 compared to BT474, which was even stronger than the one in the trastuzumab treated BT474 cell line (S2 Table), underlining the benefit of a reduced CAV1 expression for drug efficacy. To further examine the role of CAV1 in trastuzumab treatment, we analyzed its expression in public available data from the transNOAH breast cancer trial (GEO series GSE50948) [31]. The original NeOAdjuvant Herceptin (NOAH) trial revealed the benefit of trastuzumab addition to treatment with neoadjuvant chemotherapy in HER2+ breast cancer patients [32]. We selected 11 patient samples with similar receptor status as BT474, i.e. estrogen receptor (ER)+/progesterone receptor (PR)+/HER2+ [33], of which four received neoadjuvant doxorubicin/paclitaxel treatment followed by cyclophosphamide/methotrexate/fluorouracil. The remaining seven patients received trastuzumab in addition for one year. CAV1 was differentially expressed between the two groups (p < 0.05) with an approximately two-fold upregulation in the trastuzumab treated group (Fig. 4) supporting our observation. S1 Fig. shows the corresponding boxplots for all of the 54 candidate genes.

For IGFBP3, an insulin-like growth factor binding protein (IGFBP), several publications already hint at a positive association between its expression levels and trastuzumab efficacy. The corresponding biological processes of the Gene Ontology (GO) annotation included regulation of cell growth, negative regulation of signal transduction, protein phosphorylation and cell proliferation as well as positive regulation of apoptotic process, IGF receptor (IGFR) signaling pathway and MAPK cascade. According to Dokmanovic et al., trastuzumab enhanced IGFBP3 expression which in turn contributed to its sensitivity by growth inhibition [34]. They observed high IGFBP3 levels in the trastuzumab sensitive cell lines SKBR3 and BT474 and explained the way of growth inhibition by blocking crosstalk between the IGF-IR and HER2 signaling pathway. IGFBP2 and IGFBP3 were considered as potential predictive biomarkers for trastuzumab resistance by them, since IGFBP3 interfered with IGF-I-mediated mitogenic signaling and reduced IGFBP2-induced HER2 activation. Jerome et al. also observed that recombinant human IGFBP3 (rhIGFBP3) inhibited growth of HER2+ breast tumors and potentiated trastuzumab activity [35]. They observed that IGF-IR activation countered early effects of trastuzumab on HER2 signaling via AKT and ERK1/2. Downregulated phosphorylation of both proteins was associated with rhIGFBP3 inhibition of tumor growth. That is why Jerome et al. suggested simultaneous blockade of HER2 and IGF-IR pathways via rhIGFBP3 plus trastuzumab for HER2+ breast cancer patients. Lu et al. also reported a relation between IGF-IR signaling and trastuzumab resistance in HER2+ breast cancer cell models [36]. Their explanation for enhanced trastuzumab-induced growth inhibition by IGFBP3 was that IGFBP3 interferes with ligand-IGFR interactions.

#### Differentially expressed genes between HCC1954 and BT474—indicators for intrinsic resistance

**Fig 5:**
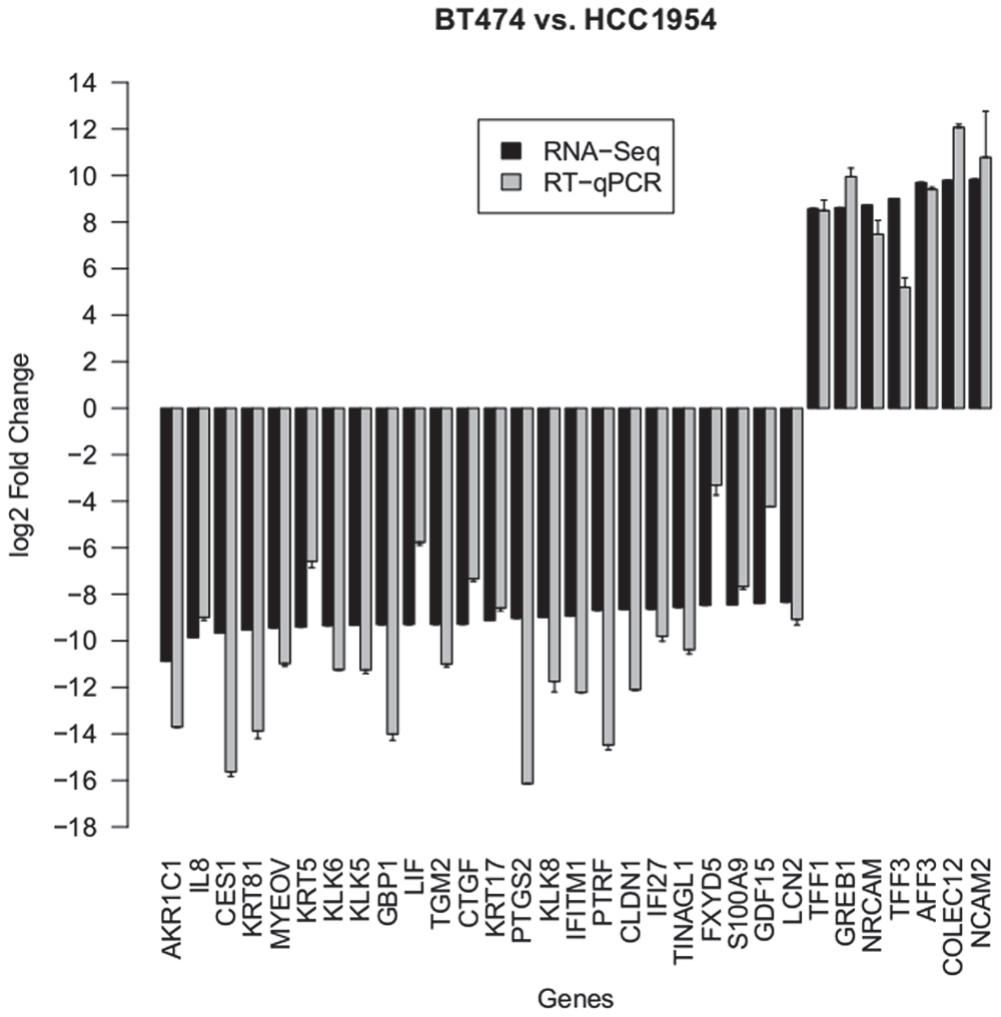
Fold Changes of DE genes (BT474 vs. HCC1954). The barchart displays the log2 fold changes of validated candidate genes, which showed significant differences in their expression in BT474 and HCC1954, respectively. Positive values indicate an upregulation in BT474. Negative values indicate an upregulation in HCC1954. Black bars denote values resulting from RNA-Seq analysis. Gray bars denote values resulting from RT-qPCR analysis.

Among the validated candidate genes, 24 had negative log2 fold changes (FC) and seven had positive ones. A negative log2 FC indicated significantly higher expression in HCC1954 compared to BT474. The genes with a positive log2 FC were significantly lower expressed in HCC1954 compared to BT474 and might contribute to trastuzumab efficiency. Fig. 5 shows the log2 FC resulting from RNA-Seq and RT-qPCR analysis, respectively. Table 2 lists the gene descriptions as well as the log2 FC and fdr of the RNA-Seq analysis. It is an excerpt of S3 Table.

**Table 2:** Differentially expressed genes between HCC1954 and BT474.

HGNC Symbol	Description	Log2 FC	FDR
AKR1C1	aldo-keto reductase family 1 member C1	-10.88	4.6e-02
CES1	carboxylesterase 1	-9.67	4.6e-02
CLDN1	claudin 1	-8.65	4.6e-02
CTGF	connective tissue growth factor	-9.28	4.6e-02
FXYD5	FXYD domain containing ion transport regulator 5	-8.47	4.6e-02
GBP1	guanylate binding protein 1 interferon-inducible	-9.30	4.9e-02
GDF15	growth differentiation factor 15	-8.38	4.6e-02
IFI27	interferon alpha-inducible protein 27	-8.63	4.6e-02
IFITM1	interferon induced transmembrane protein 1	-8.94	4.6e-02
IL8	interleukin 8	-9.86	4.6e-02
KLK5	kallikrein-related peptidase 5	-9.32	4.6e-02
KLK6	kallikrein-related peptidase 6	-9.35	4.6e-02
KLK8	kallikrein-related peptidase 8	-8.99	4.6e-02
KRT17	keratin 17	-9.12	4.6e-02
KRT5	keratin 5	-9.41	4.6e-02
KRT81	keratin 81	-9.53	4.6e-02
LCN2	lipocalin 2	-8.34	4.6e-02
LIF	leukemia inhibitory factor	-9.29	4.9e-02
MYEOV	myeloma overexpressed	-9.44	4.6e-02
PTGS2	prostaglandin-endoperoxide synthase 2 (prostaglandin G/H synthase and cyclooxygenase)	-9.03	4.6e-02
PTRF	polymerase I and transcript release factor	-8.68	4.6e-02
S100A9	S100 calcium binding protein A9	-8.45	4.6e-02
TGM2	transglutaminase 2	-9.29	4.6e-02
TINAGL1	tubulointerstitial nephritis antigen-like 1	-8.55	4.6e-02
AFF3	AF4/FMR2 family member 3	9.70	4.6e-02
COLEC12	collectin sub-family member 12	9.79	4.6e-02
GREB1	growth regulation by estrogen in breast cancer 1	8.62	4.6e-02
NCAM2	neural cell adhesion molecule 2	9.83	4.6e-02
NRCAM	neuronal cell adhesion molecule	8.73	4.6e-02
TFF1	trefoil factor 1	8.57	4.6e-02
TFF3	trefoil factor 3 (intestinal)	9.01	4.6e-02

The table displays the results of the RNA-Seq data comparison between BT474 and HCC1954. It holds the description, log2 fold change (FC) and fdr of the differentially expressed genes. Positive log2 fold changes indicate higher gene expression in BT474, while negative log2 fold changes indicate higher gene expression in HCC1954.

Due to the large amount of candidates in this DE analysis, we focused on the ones which have already been mentioned in the context of trastuzumab efficacy, namely IL8, PTGS2, GDF15 and LCN2, all of which were upregulated in HCC1954. We analyzed the remaining genes of this group more generally in the context of breast cancer.

**Upregulated genes in HCC1954 compared to BT474** IL8 (interleukin 8) is a chemotactic and inflammatory cytokine, which is produced upon inflammatory stimulation [37]. IL8 is associated with biological processes such as positive regulation of neutrophil chemotaxis, neutrophil activation, inflammatory and immune response. It was overexpressed in the resistant cell line HCC1954 compared to BT474. Korkaya et al. observed that PTEN downregulation and HER2 overexpression synergize to increase the expression of IL8 and IL6. Both of them are speculated to participate in an inflammatory loop, mediating trastuzumab resistance in HER2+ breast cancer by expanding the cancer stem cell population [38]. This loop implies AKT and subsequent NF-*κ*B activation. Interestingly, HCC1954 is already known for dysregulated PI3K/AKT signaling due to its PIK3CA mutation. Our results point to an important role of IL8 in driving trastuzumab resistance via a hyperactive PI3K pathway.

PTGS2 (cyclooxygenase prostaglandin-endoperoxide synthase 2), also known as COX2, was upregulated in HCC1954 as well and is connected to inflammation and carcinogenesis. Accordingly, it is usually not detectable in healthy tissues but can be induced in response to proinflammatory cytokines, growth factors and tumor promoters [39]. Ristimäki et al. stated that PTGS2 was detected in many breast tumors, correlated with poor patient prognosis, and its overexpression was shown to correlate with HER2 oncogene amplification in breast cancer [39]. Wang et al. also reported that nuclear HER2 bound at and transactivated the PTGS2 promoter, determining PTGS2 gene expression [40]. They further showed that trastuzumab triggered HER2 protein depletion and mitigated the association between nuclear HER2 and the PTGS2 promoter. Flowers and Thompson treated SKBR3 with t10c12 conjugated linoleic acid (CLA) and observed a HER2 suppression as well as enhanced apoptosis [41]. This confirmed the expected anti-tumor properties of t10c12 CLA via inhibition of NF-*κ*B activity and PTGS2-derived prostaglandin E2 (PGE2). Both factors play a role in resistance against HER2-targeted therapy with trastuzumab, which usually improves disease-free survival (DFS) for women with HER2+ breast cancers. In this context we derived the GO terms ‘response to fatty acid’, ‘positive regulation of NF-*κ*B import into nucleus’ and ‘positive regulation of prostaglandin biosynthetic process’. Regulation of HER2 oncogene expression by PTGS2 and PGE2 was analyzed by Benoit et al. [42]. According to their study, high levels of the proinflammatory and antiapoptotic enzyme PTGS2 have already been detected in HER2+ tumors, linked to HER2-mediated induction of PTGS2 gene transcription. In turn, PTGS2 expression and synthesis of its enzymatic product PGE2 lead to enhanced HER2 expression, which is known to correlate with adverse prognosis in breast cancers. Thus, HER2 and PTGS2 transcriptionally regulate each other in a positive feedback loop. Indeed, PTGS2 inhibition reduced HER2 protein levels, increased cancer cells sensitivity to chemotherapeutic treatment and acted synergistically with trastuzumab. The association between PTGS2 and HER2 overexpression was also analyzed by Howe et al. [43]. They emphasize that PTGS2 or derived prostaglandins are known to promote angiogenesis and invasiveness of diverse cancer types. We inferred the GO term ‘positive regulation of cell migration involved in sprouting angiogenesis’ in this context. Especially the HER2/HER3 signaling pathway was shown to regulate expression of PTGS2 and its associated pathway [44]. As the HER2/HER3 heterodimer is known to stimulate the PI3K pathway [45], it confirms our observation that PTGS2 was overexpressed in the PIK3CA mutated HCC1954 cell line. Basu et al. detected that PTGS2 inhibition decreased cell growth and promoted apoptosis in metastatic breast cancer [46]. Indeed, PTGS2 is known to positively regulate apoptotic processes and growth factor (GF) production, e.g. TGF-*β*, VEGF, fibroblast GF (FGF) and platelet-derived GF (PDGF). As a link is assumed between overexpression of HER2 and PTGS2 activity in breast cancer, Gianni speculated that a combination of PTGS2 inhibitors with trastuzumab may be efficient in high-risk patients [47]. Within a corresponding phase II study, Dang et al. tested a combined treatment of metastatic breast cancer patients who have progressed after prior trastuzumab-based treatments with trastuzumab and celecoxib, a PTGS2 inhibitor [48], which already showed promising results in colorectal cancer [49]. Unfortunately, they revealed that the combination was not active in patients with HER2-overexpressing, trastuzumab-refractory disease. Cho et al. suggested to combine the HIV protease inhibitor nelfinavir and celecoxib or analogs to increase endoplasmic reticulum stress and toxicity in human breast cancer cell lines that were resistant to trastuzumab [50]. The background was that moderate activity of the stress response system exerts anti-apoptotic function, supporting tumor cell survival and chemoresistance, whereas more severe aggravation may turn on its pro-apoptotic module. In this context PTGS2 was also associated with the GO term ‘positive regulation of cell death’. Morrison et al. reported a marginal association of abnormal copy number of PTGS2 with objective trastuzumab response in metastatic breast cancer [51].

GDF15 (growth differentiation factor 15) was overexpressed in HCC1954, too. Hence, it could contribute to trastuzumab resistance as well. According to GO annotation, it is associated to cell-cell signal transduction, especially to the TGF-*β* signaling pathway. Indeed, Joshi et al. observed GDF15-mediated HER2 phosphorylation reducing trastuzumab sensitivity of HER2+ breast cancer cells [52]. They regarded GDF15-mediated activation of TGF-*β* receptor-Src-HER2 signaling crosstalk as a mechanism of trastuzumab resistance.

Another upregulated gene in HCC1954 was LCN2, encoding the chaperone protein lipocalin 2. Kumandan et al. reported that LCN2 is known to be highly upregulated during the unfolded protein response (UPR) in an NF-*κ*B-dependent manner [53]. As UPR activates the HER2/PI3K/AKT/NF-*κ*B signaling pathway, LCN2 is assumed to play an important role as a common downstream effector molecule. Kumandan et al. observed that trastuzumab inhibited the expression of LCN2 in SKBR3 cells. The downregulation of LCN2 was abrogated by activation of the UPR. An increase of LCN2 transcription and secretion was observed. It was speculated that UPR induction bypasses trastuzumab-mediated inhibition of the PI3K pathway. Lin et al. observed that exposure of human endometrial carcinoma cells to LCN2 for more than 24 hours reduced LCN2-induced cell apoptosis, changed proliferation and upregulated, amongst other cytokines, IL8 secretion [54]. Recombinant IL8 lead to decreased activity of the apoptotic enzyme caspase-3. The addition of IL8 antibodies resulted in significantly increased caspase-3 activity and decreased cell migration, indicating an important role of IL8 in the induction of cell migration. Interestingly, we associated the GO term ‘regulation of apoptotic process’ with LCN2 and UPR related GO terms with IL8, namely ‘activation of signaling protein activity involved in unfolded protein response’ and ‘endoplasmic reticulum unfolded protein response’. Hence, IL8 does not only seem to be stimulated by UPR-induced LCN2, but also participates in the activation of UPR. This points to a synergistic feedback mechanism involving IL8 and LCN2, which could contribute to trastuzumab resistance.

Many of the remaining validated candidates which were differentially expressed between BT474 and HCC1954 with overexpression in HCC1954 have already been mentioned in the context of breast cancer, mainly as tumor promoters.

Regarding AKR1C1 (aldo-keto reductase family 1, member C1), a loss of expression and related decreased progesterone catabolism, leading to decreased growth in the presence of progesterone, has been observed in breast tumors by Ji et al. [55]. They speculated that loss of AKR1C1 in combination with increased PR expression may enhance progesterone-dependent PR activation, which plays an essential role in breast development and cancer formation. This fits to our observation that AKR1C1 was downregulated in BT474 compared to HCC1954. BT474 is known for its PR+ status [33], while HCC1954 is negative for expression of ER and PR according to the manufacturer’s product information.

A relation to steroid receptors was also found for MYEOV (myeloma overexpressed), which DNA amplification correlated with ER+ and PR+ cancer in a study of Janssen et al. [56]. Although MYEOV is overexpressed in breast tumors, they did not detect its expression in BT474. They showed that abnormal expression levels of the candidate oncogene correlated only partially with DNA amplification and that overexpression could act independently or cooperatively with the cell cycle regulator Cyclin D1 to favor tumorigenesis. Hence, overexpression of MYEOV in HCC1954 underlined the outstanding role in tumor promotion of this cell line.

The keratin KRT5 (CK5) is related to progesterone as well, since CK5+ cells were shown to lose ER and PR expression [57], fitting to our observation of overexpression in HCC1954 compared to BT474. Furthermore Axlund et al. observed that cells overexpressing CK5 are more invasive, sphere-forming, and quiescent with an increased resistance to endocrine and chemotherapy [57]. CK5 is a basal marker in breast carcinoma, related to poor prognosis and to unfavorable overall survival (OS) as well as relapse-free interval [58]. Apart from KRT5, two further keratins were overexpressed in HCC1954, namely KRT17 (CK17) and KRT81 (KRTHB1). CK17 is a basal-type cytokeratin as well, and the expression of CK17 and CK5/6 appears to define a group of breast tumors with a poor clinical outcome, especially high mortality rates [59].

Another group of related genes was formed by the three kallikrein-related peptidases KLK5, KLK6 and KLK8, all of them overexpressed in HCC1954. High KLK5 expression was found more frequently in node-positive and ER-negative (ER-) patients [60], confirming our observation of upregulation in HCC1954. Yousef et al. showed that KLK5 overexpression was a predictor of reduced DFS and OS in breast cancer, and hence a marker of poor prognosis. They regarded KLK5 as a candidate that might stimulate cellular growth, angiogenesis or degradation of the extracellular matrix (ECM) [60]. Analogously higher KLK6 protein levels were found to be associated with tumors which are negative for ER and PR [61], as it is the case for HCC1954. Yousef et al. observed that the closely localized kallikreins KLK5, KLK6 and KLK8 are in general downregulated with a similar pattern of differential expression in breast cancer [62].

A promoting effect on tumorigenesis and metastasis of breast cancer via the AKT/mTOR signaling pathway, independent of ER status, was detected for the multi-functional cytokine protein LIF (leukemia inhibitory factor) by Li et al. [63]. We observed an upregulation of LIF in HCC1954. Li et al. reported that its overexpression has been observed in several types of cancers including breast cancer and is associated with a poorer relapse-free survival. They showed that LIF promotes cell proliferation and growth of breast cancer cells in vitro, and growth of xenograft breast tumors in vivo. Furthermore it promoted invasion and migration of breast cancer cells in vitro and metastasis of breast cancer in vivo.

A further gene upregulated in HCC1954 was TGM2. As reviewed by Agnihotri et al., TGM2 is a stress-responsive gene, encoding the multifunctional ubiquitously expressed enzyme transglutaminase 2 (TG2) which seems to play a crucial role in promoting an aggressive phenotype in mammary epithelial cells [64]. Its expression is upregulated during inflammation and wounding, as it crosslinks ECM component proteins and stabilizes the matrix for increased cell attachment and motility. It has also been observed that anti-apoptotic TGM2 is upregulated in cancer, especially those resistant to chemo- and radiation therapy and those isolated from metastatic sites. In mammary epithelial cells, chronic TG2 expression initiates signaling contributing to drug resistance and an invasive phenotype, and high expression levels are associated with activation of indicators of aggressive tumors, such as AKT and NF-*κ*B in a feedback loop. Above that TG2 expression induces epithelial-to-mesenchymal transition and confers cancer stem cell trait, both of which have been implicated in metastasis and resistance to standard therapies. TG2 expression in tumor samples is associated with poor disease outcome, increased (chemotherapeutic) drug resistance, and increased incidence of metastasis [64].

Another gene highly upregulated in HCC1954 was CTGF (connective tissue growth factor). Although literature is ambivalent, assigning either a tumor suppressor or enhancer role to this gene, the oncogenic character of CTGF seems to dominate. Its expression is elevated in advanced stages of breast cancer, and Chen et al. observed enhanced cellular migratory ability in breast cancer cells overexpressing CTGF [65]. According to them, CTGF mediated ERK1/2 activation and hence cellular migration. Additionally, CTGF mediated upregulation of the prometastatic gene S100A4, dependent on ERK1/2. This points to an important role of CTGF in migration and invasion, and supports other investigators who linked overexpression to tumor size and lymph node metastasis or related CTGF to angiogenesis and bone metastasis in breast cancer. Interestingly, Chen et al. detected extremely low or no levels of CTGF mRNA in BT474, confirming our observation. Furthermore, the relation of CTGF and S100A4 is of special interest, as we also detected a downregulation of the S100 calcium binding protein family member S100A9 in BT474 compared to HCC1954. Gonçalves et al. found an association between S100A9, a protein expressed in invasive breast cancer, with basal subtypes as well as corresponding poor differentiation and prognosis value [66]. Most interestingly, they inferred BT474 and HCC1954 as different molecular subtypes, i.e. BT474 as luminal-like, and HCC1954 clustered together with basal-like cell lines, although no specific type was assigned. S100A9 expression was, amongst others, tightly associated with high grade, negative ER and PR status, and HER1 and HER2 expression. Furthermore, it was significantly associated with reduced metastasis-free and overall survival.

PTRF (polymerase I and transcript release factor) was upregulated in HCC1954 as well in our study. The RT-qPCR data of BT474 and HCC1954, respectively, revealed similar expression patterns for PTRF and CAV1 (S2 Table). Verma et al. detected that PTRF (cavin-1) and caveolin 1 are lost coordinately in cancer cells [67]. Membrane recruitment of the cavin complex requires caveolin 1, and PTRF is important for regulating caveolin 1 membrane dynamics.

Another upregulated gene in HCC1954 was CLDN1, encoding the tight junction protein claudin 1. Di Cello et al. pointed out that CLDN1 may alternatively function as a tumor suppressor or as an oncogene, but in breast cancer they assumed a tumor suppressor role, since CLDN1 is frequently downregulated in this type of cancer, associated with recurrence, metastasis, and reduced survival [68]. They explained its downregulation by epigenetic silencing via DNA methylation. Interestingly, they demonstrated this using amongst others the BT474 cell line, which showed low CLDN1 expression. According to their results, in breast cancer, CLDN1 expression varies according to the molecular subtype. While methylation of the CLDN1 promoter CpG island was frequent in ER+ breast cancer, it was not the case in most of the ER- breast cancers samples, of which some overexpressed CLDN1. HCC1954 fell into a cluster composed mostly of ER-, basal-like cell lines, which featured loss of methylation at sites downstream of the CpG island. Lu et al. detected positive associations between claudin 1 and CK5/6 and HER1 [69], which is of special interest, as HER1 and HER2 function as heterodimers [45], and we detected CK5 as being overexpressed in HCC1954 as well. Above that they found a negative association between CLDN1 and ER, fitting to our results of upregulation in the ER- cell line HCC1954. Confirming the results of Di Cello et al., Lu et al. observed high levels of CLDN1 in the basal-like subtype.

Like CLDN1, FXYD5 (FXYD domain containing ion transport regulator 5), also known as dysadherin, is involved in cell-cell adhesion, which was downregulated in BT474 compared to HCC1954 as well. Lee et al. detected no dysadherin expression in BT474, likely due to its ER+ status, as dysadherin is expressed in the more aggressive ER- breast cancer [70]. In their study the introduction of dysadherin cDNA into BT474 enhanced levels of AKT phosphorylation. Hence, they speculated that dysadherin, which overexpression is a predictor of metastasis, invasion and poor prognosis, might contribute to breast cancer progression through ER-dependent AKT activation.

**Upregulated genes in BT474 compared to HCC1954** Many of the remaining validated candidates which were differentially expressed between BT474 and HCC1954 with overexpression in BT474 are induced by estrogen receptors (ER). This is explainable by the ER status of the cell lines. While BT474 is known to be an ER+/PR+ cell line [33], HCC1954 is an ER-/PR- one.

COLEC12 (collectin sub-family member 12) was highly overexpressed in BT474 and is likely to be an ER*β* primary target gene, indicating an antiproliferative role [71].

Analogously, TFF3 (trefoil factor 3) expression has been reported to correlate with ER*α*, hinting at induction by this receptor subtype. Additionally, a positive correlation was observed between PR and TFF3 protein expression by Ahmed et al. [72], confirming our observed TFF3 overexpression in the BT474 cell line. According to their results, TFF3 expression is induced by estrogens and hardly detected in breast tumors which do not express ER. The luminal subtype A, which is primarily composed of ER+ tumors, demonstrates a better prognosis than other breast cancer subtypes and is characterized by overexpression of estrogen-related genes such as TFF3 and ER*α* [73]. Ahmed et al. observed a higher TFF3 expression in well-differentiated tumors and an association with low histological grade. Nevertheless, they speculated that in invasive cancer the positive characteristics of TFF3 turn into oncogenic ones [72]. Obviously, the ER+/PR+ cell line BT474 benefits from the estrogen-related positive TFF3 effects. The ER*α*-responsive gene TFF1 is known to be coexpressed with TFF3 in ER+ malignant breast cancer cells and is likewise upregulated by estrogens. Although TFF1 is a disseminated tumor cells detection marker, TFF1 and TFF3 are components of a luminal epithelial signature defining a well-differentiated, low-grade breast cancer subtype [74].

The upregulation of NRCAM (neuronal cell adhesion molecule) in BT474 can be ascribed to the ER status, too. This gene was reported to be expressed in ER+ breast cancer cell lines but not in ER- ones [75].

Another estrogen-induced, ER-responsive gene upregulated in BT474 was GREB1 (growth regulation by estrogen in breast cancer 1). It is an important factor in ER induced proliferation in breast cancer cells [76]. Liu et al. reported that overexpression of the ER cofactor GREB1 also increased the clonogenic ability in breast cancer cells and that overexpression was observed in ER+ breast cancer patients compared to ER- ones [77]. They stressed that reduced levels of GREB1 are predictive of worse disease outcome for breast cancer patients in general, and for ER+ patients in particular.

Interestingly, the RT-qPCR data of all genes overexpressed in BT474 compared to HCC1954 revealed a three-fold overexpression in the trastuzumab treated version of BT474 compared to its untreated version (S2 Table). Evans et al. already speculated that in HER2+/ER+ tumors increased TFF1 expression and estrogen signaling may occur as a result of inhibiting the EGFR/HER2 signaling pathway [78]. Collins et al. confirmed that trastuzumab treatment upregulated ER transcriptional activity and TFF1 expression [79]. However, they reported a mostly poor response to trastuzumab treatment in HER2+ cells with co-expression of the steroid pathway.

**Fig 6:**
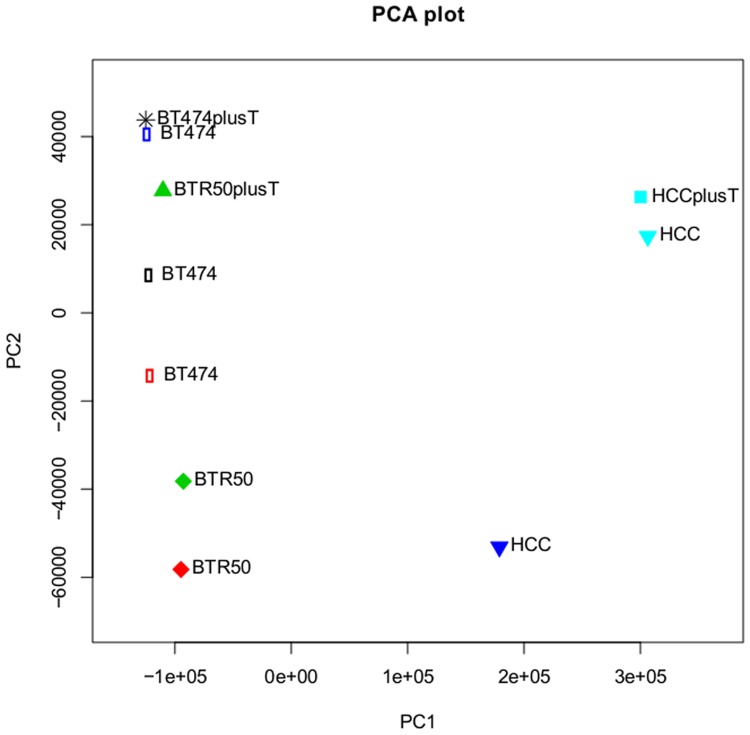
Principle Component Analysis (PCA) of all measured samples. The plot displays the result of a PCA on the pairwise normalized RNA-Seq expression values of all 23367 annotated genes in the samples BT474, BTR50 and HCC1954 with and without trastuzumab (T) treatment. Same colors denote that the samples belong to the same conducted statistical test and thus were normalized in pair. The five two sample tests included *BT474 vs. HCC1954*, *BT474 vs. BTR50*, *HCC1954+T vs. HCC1954*, *BTR50+T vs. BTR50*, and *BT474+T vs. BT474*.

Furthermore, the RT-qPCR data also show that the mentioned genes are at least as strongly expressed in the BTR50 cell line compared to BT474, and not downregulated as in HCC1954 (S2 Table). This hints at a strong discrepancy between expression profiles in the cell line models of intrinsic and acquired drug resistance. This could be explained by the individual characteristics of the HCC1954 cell line, maybe also driven by specific SNPs, which are analyzed in the following section. Also, the Principal Component Analysis (PCA) plot of the pairwise normalized cell lines, considering all 23367 annotated genes, demonstrates that the intrinsically resistant cell line HCC1954 differs much more from BT474 than BTR50, and that trastuzumab treatment hardly influences expression in this cell line (Fig. 6).

### SNP analysis of genes associated with trastuzumab efficiency

We searched for SNPs in the three untreated cell lines HCC1954, BTR50 and BT474. In the following, we describe the results for the validated gene candidates of the DE analysis on BT474 and its trastuzumab treated version (ALPP, CALCOCO1, CAV1, CYP1A2, IGFBP3) as well as the candidates of the DE analysis on BT474 vs. HCC1954 which were already reported in the context of trastuzumab efficacy, namely GDF15, IL8, LCN2 and PTGS2. Additionally we focus on HER2 due to the HER2+ status of the cell lines, PIK3CA due to the known mutation in HCC1954, as well as MAPK1 and AKT1 as main players in the MAPK and PI3K/AKT pathway, respectively. S1 Variant Call Format File, S2 Variant Call Format File and S3 Variant Call Format File store the SNPs called in the HCC1954 (S1 Variant Call Format File), BTR50 (S2 Variant Call Format File) and BT474 (S3 Variant Call Format File) sample.

#### SNPs in the HCC1954 cell line

S1 Variant Call Format File stores the SNPs called in the HCC1954 sample. For eight of the 13 genes of interest we detected SNPs in HCC1954 which passed our filter criteria.

We called 13 variations for HER2, five variations for MAPK1, three variations for PTGS2 and AKT1, respectively, two variations for IGFBP3 and one SNP for PIK3CA, CAV1 and IL8 each. The ALPP SNP at position 233243981 on chromosome 2 (rs13026692, COSM1162692) had a read depth of nine, just slightly failing our filter criterion. The related locations and reference SNP ID numbers [8] are listed in Table 3. 80% of the SNPs have already been listed in the *dbSNP* database but just 40% of those have been analyzed more deeply in literature, even less in the context of breast cancer.

**Table 3:** SNPs called in the HCC1954 cell line.

Gene	Chromosome	Position	Variation
AKT1	14	105235824	rs58565216
AKT1	14	105236287	rs35416681
AKT1	14	105241304	rs2230506
CAV1	7	116200554	rs374946197
HER2	17	37855834	rs4252596
HER2	17	37869895	-
HER2	17	37870378	rs2934967
HER2	17	37871081	-
HER2	17	37876835	rs2952156
HER2	17	37877412	rs2952157
HER2	17	37877447	rs11653998
HER2	17	37878371	-
HER2	17	37878574	-
HER2	17	37878635	-
HER2	17	37879030	rs2088126
HER2	17	37884037	rs1058808, rs370420724, CM087578
HER2	17	37885332	rs2952158
IGFBP3	7	45957678	rs3793345
IGFBP3	7	45960645	rs2854746, CM057457
IL8	4	74609045	rs1126647, CR025956
MAPK1	22	22115004	rs6928
MAPK1	22	22115353	rs9340
MAPK1	22	22115498	rs3810610
MAPK1	22	22116202	rs13943
MAPK1	22	22116467	rs1063311
PIK3CA	3	178952085	rs121913279, COSM776, COSM775, COSM249874, COSM94987, COSM94986
PTGS2	1	186641626	rs2853805
PTGS2	1	186642429	rs2206593
PTGS2	1	186643058	rs5275

The table displays information about chromosomal location of the detected SNPs in the genes of interest as well as corresponding SNP ID numbers [8]. In case of missing ID numbers (‘-’), we likely inferred a novel mutation.

Regarding rs2952156 and rs4252596 of HER2, Einarsdóttir et al. did not observe an association with breast cancer survival [80]. Analogously, Benusiglio et al. analyzed whether common polymorphisms (frequency ≥ 5%) in HER2 were associated with breast cancer risk in a white British population [81]. Although they considered rs4252596 near the promoter as potentially regulatory functional and the nonsynonymous coding SNP rs1058808 of HER2 potentially affecting tyrosine kinase activity or protein structure, there was no evidence that these variations were associated with breast cancer. Breyer et al. analyzed the role of genetic variation of the HER2 gene in breast cancer risk in a study of invasive breast cancer cases from the Shanghai Breast Cancer Study [82]. According to their results, HER2 resides within a locus of high linkage disequilibrium, composed of three major ancestral haplotypes in the study population. These haplotypes are marked by simple tandem repeat and SNPs including the missense variants I655V and P1170A (rs1058808). However, they did not observe an association of the SNP haplotypes with breast cancer predisposition.

For PTGS2 we detected the polymorphism rs5275 in the 3’ untranslated region of the PTGS2 gene. Festa-Vasconcellos et al. investigated the association between PTGS2 haplotypes and histopathological parameters with prognostic value on clinical outcome, i.e. risk of tumor recurrence, of breast cancer [83]. Their study involved women under treatment for non-metastatic breast cancer who were genotyped for rs689465, rs689466, rs20417 and rs5275. Eight haplotypes were inferred with a significant difference in their distribution as a function of tumor size, ER and HER2 status. No associations were found between rs5275 solely and histopathological features, which was in line with the observations of Gerger et al. [84] and Abraham et al. [85] who found no associations between rs5275 and DFS or OS of breast cancer patients. Above that two breast cancer meta-analyses indicated no strong risk association for rs5275 [86, 87]. Though, Festa-Vasconcellos et al. observed an rs5275 dependent positive association between higher tumor size and the haplotype formed by rs689465G, rs689466A, rs20417C and rs5275T. At least SNPs rs689465 and rs5275 were required to infer this haplotype, which was further positively associated with ER and PR negativity and HER2 positivity. Moore et al. supported an important influence of rs5275, reporting it disrupts micro-RNA-mediated regulation of PTGS2 mRNA degradation, leading to increased mRNA stability and PTGS2 protein levels [88]. Jung et al. analyzed polymorphisms of apoptosis-related genes and their impact on the survival of patients with early invasive ductal breast cancer [89]. The PTGS2 gene polymorphism rs5275 in a dominant model of the C allele was associated with a higher distant DFS. Cox et al. genotyped the five most common PTGS2 polymorphisms (rs20417, rs5277, rs20432, rs5275, and rs4648298) in the Nurses’ Health Study to test for an association with breast cancer risk [90]. The SNP rs5275 was associated with a decrease in breast cancer risk and further genotyped in the Nurses’ Health Study 2 and Harvard Women’s Health Study. In pooled analyses, women homozygous for the T allele at rs5275 had a 20% lower risk of breast cancer than those homozygous for the C allele. Cox et al. concluded that this polymorphism may be associated with a decrease in breast cancer risk among Caucasian women, but is not associated with an increased risk of breast cancer.

We further identified the IGFBP3 SNP rs2854746. Qian et al. evaluated IGF-I and IGFBP3 genotypes in relation to their phenotypes in local breast tissues and in association with breast cancer risk for Chinese women [91]. No association was found between breast cancer risk and the IGFBP3 SNP rs2854746, but the genotype correlated with IGF-I phenotypes in tumor samples. Peptide levels of IGF-I were inversely correlated with age and menopause status. The homozygous variant genotype of rs2854746 had lower IGF-I compared to the wild type. This suggested possible influences of the SNP on IGF-I activity in local tissues. IGF-I and its major binding protein IGFBP3 were also analyzed by Su et al. due to their implication in breast carcinogenesis [92]. They examined associations between genetic variants and circulating levels of IGF-I and IGFBP3 with proliferative benign breast disease (BBD), a marker of increased breast cancer risk. Higher circulating IGFBP3 levels were significantly associated with increased risk of proliferative BBD. The SNP rs2854746 was significantly associated with circulating IGFBP3 levels. D’Aloisio et al. reported that IGFBP3 plasma levels, associated with common diseases, are influenced by common IGFBP3 SNPs, especially rs2854746, among African American and Caucasian women [93]. Similar observations were made by Patel et al. [94] and Cheng et al. [95]. However, Tamimi et al. did not detect any significant association of the common haplotypes in three haplotype blocks, of which one included rs2854746, spanning the genes encoding IGFBP1/IGFBP3, with mammographic density, one of the strongest risk factors for breast cancer [96].

Regarding PIK3CA, we inferred the SNP rs121913279, which is referring to the known H1047R mutation in HCC1954. Tong et al. detected this SNP in Chinese breast cancer patients with invasive ductal carcinomas [97].

#### SNPs in the BTR50 cell line

S2 Variant Call Format File stores the SNPs called in the BTR50 sample. For three of the 13 genes of interest we detected SNPs in BTR50 which passed our filter criteria. We called 13 variations for HER2, seven variations for MAPK1 and one variation for IGFBP3. The related locations and reference SNP ID numbers are listed in Table 4. Two third of the SNPs have already been listed in the dbSNP database but just 30% of those have been analyzed more deeply in literature, even less in the context of breast cancer. Approximately 60% of the SNPs were also detected in HCC1954 which would indicate that this intersection of mutations contributes to resistance. Interestingly, the SNP of IGFBP3 in BTR50 was not matching the ones of HCC1954.

**Table 4:** SNPs called in the BTR50 cell line.

Gene	Chromosome	Position	Variation
HER2	17	37859083	rs34284966
HER2	17	37876179	rs4252639
HER2	17	37876835	rs2952156
HER2	17	37877221	-
HER2	17	37877412	rs2952157
HER2	17	37878113	rs115334808
HER2	17	37878311	-
HER2	17	37878371	-
HER2	17	37878574	-
HER2	17	37878635	-
HER2	17	37878696	-
HER2	17	37879030	rs2088126
HER2	17	37885332	rs2952158
IGFBP3	7	45952254	rs6670
MAPK1	22	22115004	rs6928
MAPK1	22	22115353	rs9340
MAPK1	22	22115498	rs3810610
MAPK1	22	22115886	rs13515
MAPK1	22	22116202	rs13943
MAPK1	22	22116467	rs1063311
MAPK1	22	22162072	-

The table displays information about chromosomal location of the detected SNPs in the genes of interest as well as corresponding SNP ID numbers [8]. In case of missing ID numbers (‘-’), we likely inferred a novel mutation.

#### SNPs in the BT474 cell line

S3 Variant Call Format File stores the SNPs called in the BT474 sample. For two of the 13 genes of interest we detected SNPs in BT474 which passed our filter criteria. We called three variations for HER2 and five variations for MAPK1. All mutations form a subset of the corresponding ones in BTR50. This makes sense, as BTR50 has been derived from BT474 and cultured to resistance, which obviously led to additional mutations in the cell line. The related locations and reference SNP ID numbers are listed in Table 5.

**Table 5:** SNPs called in the BT474 cell line.

Gene	Chromosome	Position	Variation
HER2	17	37876179	rs4252639
HER2	17	37877412	rs2952157
HER2	17	37878113	rs115334808
MAPK1	22	22115004	rs6928
MAPK1	22	22115498	rs3810610
MAPK1	22	22115886	rs13515
MAPK1	22	22116202	rs13943
MAPK1	22	22116467	rs1063311

The table displays information about chromosomal location of the detected SNPs in the genes of interest as well as corresponding SNP ID numbers [8].

## Conclusions

The aim of the study was to infer genes and corresponding variations, which influence trastuzumab action in HER2+ breast cancer. Comparing gene expression in three cell line models of different drug response characteristics with and without trastuzumab treatment, we inferred 54 candidate genes. Most of them have already been reported in the context of breast cancer but just a small proportion in the context of trastuzumab action.

The validated selection of the candidate genes was differentially expressed between the sensitive cell line BT474 and its trastuzumab treated version, as well as between BT474 and the resistant cell line HCC1954. The candidates of the latter comparison were mainly related to the different steroid receptor status of the cell lines, mostly including tumor enhancers with upregulation in HCC1954 which have already been ascribed to ER- breast cancer subtypes. Outstanding candidates were IL8, PTGS2, GDF15 and LCN2, as those have already been reported to hinder trastuzumab action. Interestingly, mechanisms involving the PI3K pathway have been reported, which fits to the PI3K gain-of-function mutation in the resistant cell line HCC1954. Furthermore, a cooperative functionality of IL8 and LCN2 would stabilize the resistant phenotype. The two outstanding candidates revealed in the comparison of BT474 and its drug treated version were CAV1 and IGFBP3, both of which have already been reported to influence trastuzumab efficacy. While low CAV1 expression seems to support trastuzumab action, high IGFBP3 levels are associated with trastuzumab sensitivity.

We further detected that the intrinsically resistant cell line HCC1954 differs more from the sensitive cell line BT474 than the cell line BTR50, representing acquired resistance. HCC1954 was also influenced less by trastuzumab treatment than BTR50. Thus, intrinsically resistant specimens seem to respond less to trastuzumab treatment than those which acquired resistance. This was supported by the fact that we called more SNPs in the intrinsically resistant cell line.

All detected mutations in BT474 formed a subset of the ones detected in BTR50, pointing at resistance related novel mutations in the cell line which acquired resistance. Four annotated SNPs called in BTR50, which were not called in BT474, overlapped with the variations called in HCC1954, i.e. rs2952156, rs2088126 and rs2952158 of HER2, as well as rs9340 of MAPK1. Those SNPs could likely contribute to trastuzumab resistance. In general, some of the inferred SNPs are already in the focus of breast cancer research, but mostly no influence on survival or risk has been detected for single ones. However, some seem to have an impact on the phenotype in combination with further variations. Regarding the SNP rs5275 of PTGS2 in HCC1954, we found hints at a relation to breast cancer risk. Additionally, the SNP rs2854746 of IGFBP3 in HCC1954 seems to influence IGF-I and IGFBP3 expression.

In conclusion, we applied successfully the RNA-Seq method to detect differentially expressed genes as well as SNPs in the context of trastuzumab resistance in different cell phenotypes. A high proportion of validated genes by RT-qPCR confirmed our results. Based on literature research, we elucidated the role of these candidate genes or mutations in influencing trastuzumab action. A deeper analysis of single candidates, supported by additional experiments, could reveal more detailed information about mechanisms leading to trastuzumab resistance. Hence, our results provide a basis to improve personalized medicine for HER2+ breast cancer patients.

## Supporting Information

S1 Table Primers used for RT-qPCR validation. The table lists the used forward and reverse primer sequences for the 40 candidate genes which were analyzed by RT-qPCR. Additionally, the sequences for the normalizer GAPDH are listed.
(XLS)

S2 Table RT-qPCR validation data. The table lists the RT-qPCR data of 40 selected genes from the candidate set. The ViiA 7 Real-Time PCR System (Applied Biosystems, Darmstadt, Germany) was used for RT-qPCR analysis. Data analysis was performed as described in Livak and Schmittgen [27] with two biological replicates and GAPDH as reference gene.
(XLS)

S3 Table DE genes revealed by RNA-Seq analysis. The table lists the genes which were significantly differentially expressed (DE), i.e. genes with a fold change (FC) of at least two and a false discovery rate (column *padj*) of at least 0.05. Multiple testing correction of p-values (column *pval*) was done according to Benjamini and Hochberg. Genes are annotated with the HUGO Gene Nomenclature Committee (HGNC) [98] symbol and identifier (ID), Ensembl ID as well as Entrez ID [99]. Furthermore the table stores gene description as well as GO annotation for corresponding biological processes (BP). The conducted statistical test is given in the column *Test (B vs. A)*. The five two sample tests included *BT474 vs. HCC1954*, *BT474 vs. BTR50*, *HCC1954+T vs. HCC1954*, *BTR50+T vs. BTR50*, and *BT474+T vs. BT474*. The column *baseMeanA* holds the base mean, i.e. the mean of the counts divided by the size factors, for the counts of condition A. An analogous definition holds for *baseMeanB*. The column *baseMean* holds the mean of baseMeanA and baseMeanB. The FC, i.e. the ratio (baseMeanB+1)/(baseMeanA+1) is stored in the column *foldChange*. Analogously, the log2 FC is stored in the column *log2FoldChange*.
(XLS)

S1 Fig Expression data of the candidate genes in the transNOAH breast cancer trial. The boxplot displays expression (log2) data of the 54 candidate genes from seven patients treated with trastuzumab for one year in addition to neoadjuvant chemotherapy (red) and four patients treated with neoadjuvant chemotherapy only (gray). The patient data samples were selected from the transNOAH breast cancer trial (GEO series GSE50948).
(PDF)

S1 Variant Call Format File SNP calls in HCC1954. The VCF (Variant Call Format) file format is used by GATK for representation of variant calls. This file stores the SNPs called in the HCC1954 sample.
(VCF)

S2 Variant Call Format File SNP calls in BTR50. The VCF (Variant Call Format) file format is used by GATK for representation of variant calls. This file stores the SNPs called in the BTR50 sample.
(VCF)

S3 Variant Call Format File SNP calls in BT474. The VCF (Variant Call Format) file format is used by GATK for representation of variant calls. This file stores the SNPs called in the BT474 sample.
(VCF)
