# Supplementary material for: mRNA Profiling Reveals Determinants of Trastuzumab Efficiency in HER2-Positive Breast Cancer
Source: PLoS One. 2015 Feb 24;10(2):e0117818. doi: 10.1371/journal.pone.0117818 (PMC4339844; doi:10.1371/journal.pone.0117818)
Supplement: S1 Fig — The boxplot displays expression (log2) data of the 54 candidate genes from seven patients treated with trastuzumab for one year in addition to neoadjuvant chemotherapy (red) and four patients treated with neoadjuvant chemotherapy only (gray). The patient data samples were selected from the transNOAH breast cancer trial (GEO series GSE50948). (PDF) [file pone.0117818.s004.pdf]

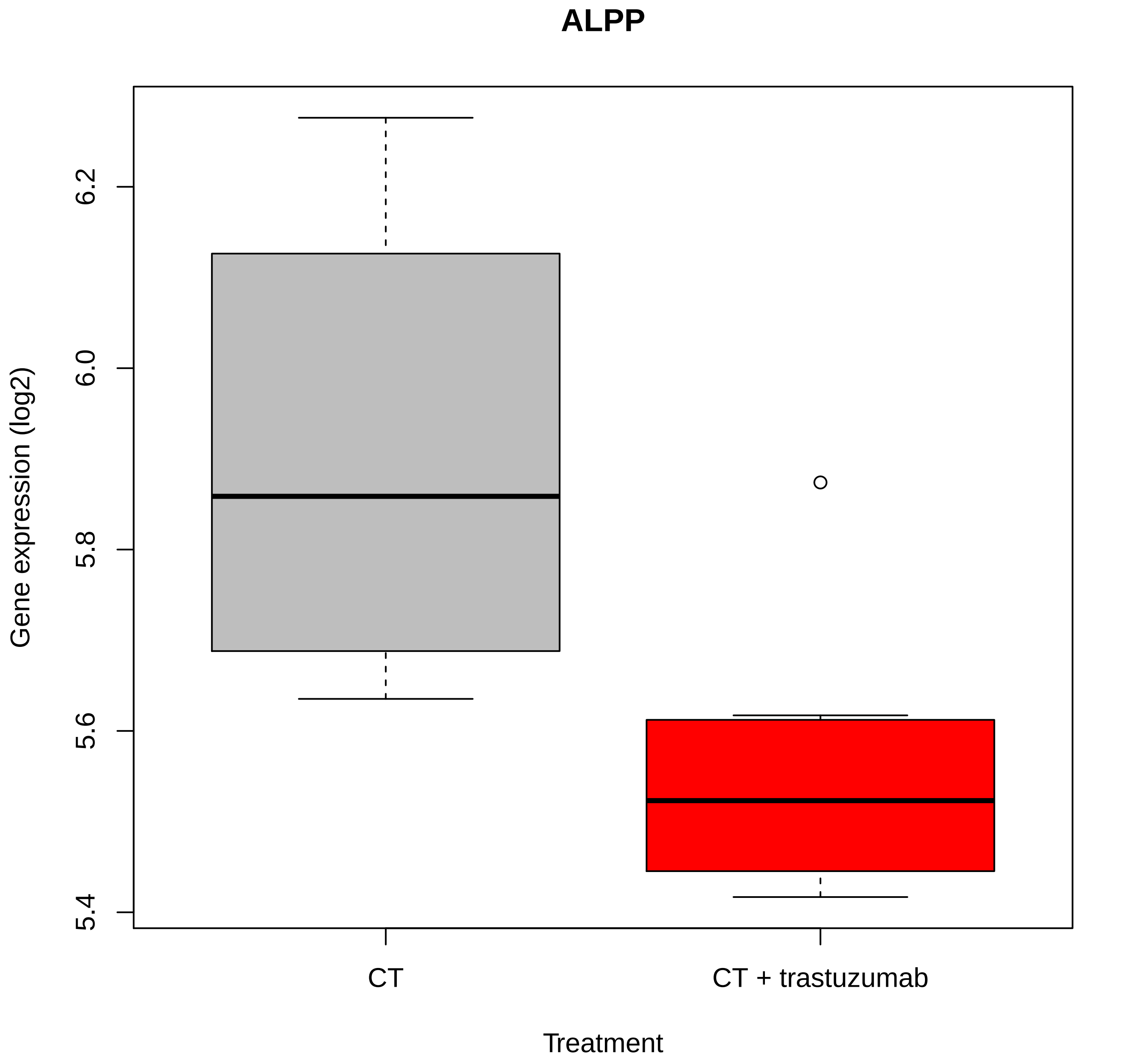
ALPP
Gene expression (log2)
6.2
6.0
5.8
5.6
5.4
CT
CT + trastuzumab
Treatment

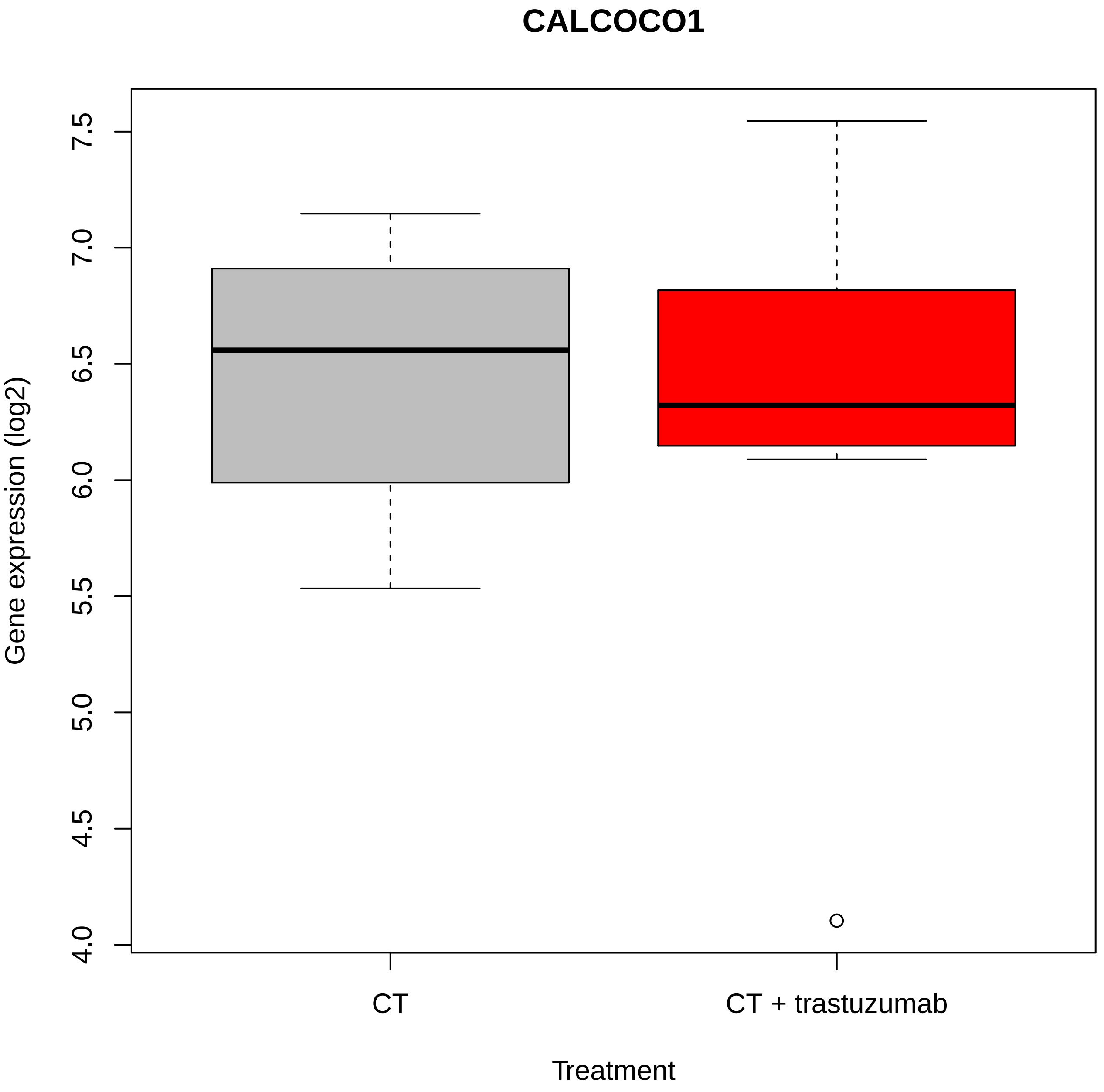
CALCOCO1
Gene expression (log2)
4.0
4.5
5.0
5.5
6.0
6.5
7.0
7.5
CT
CT + trastuzumab
Treatment

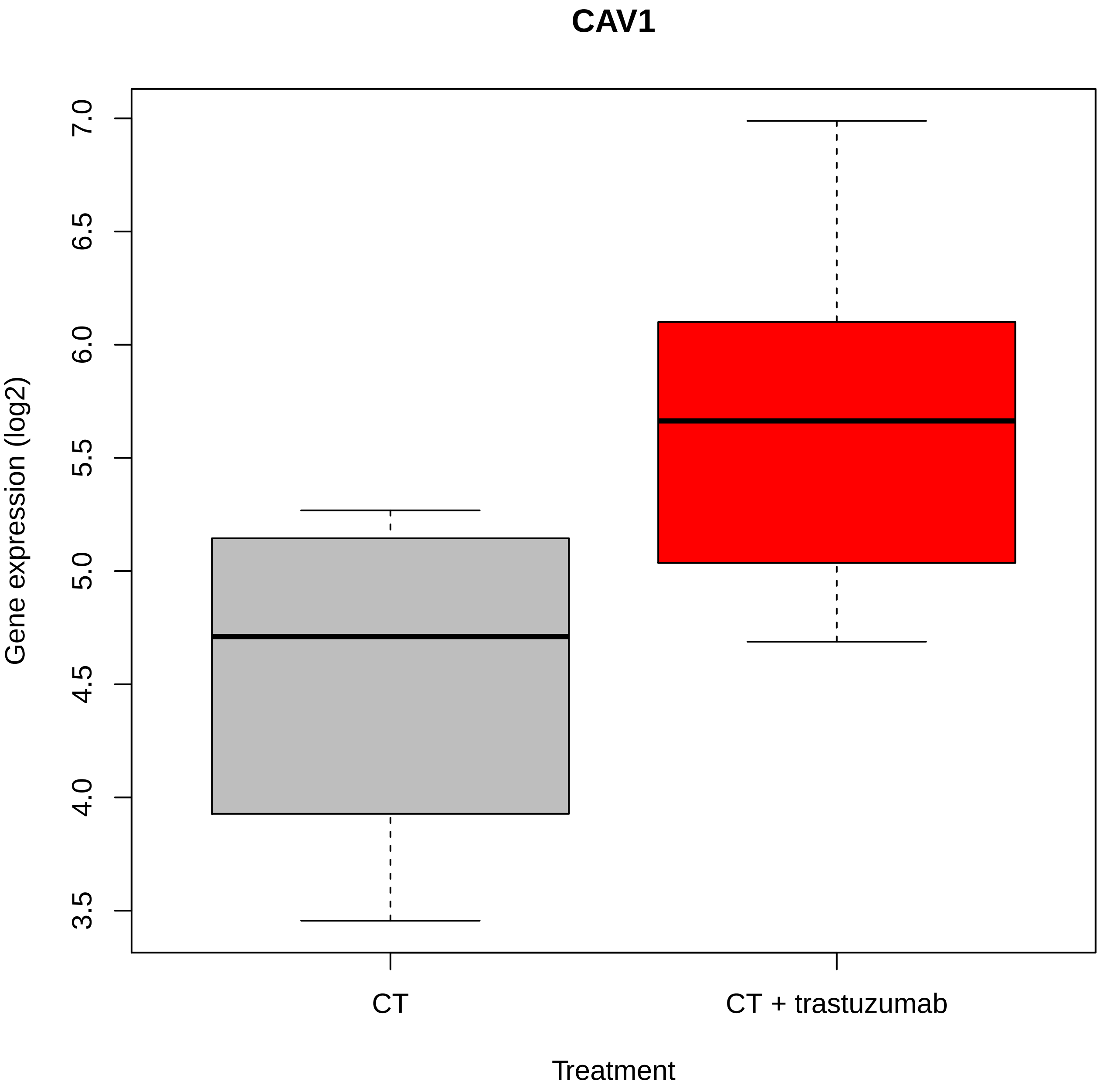
CAV1
Gene expression (log2)
3.5
4.0
4.5
5.0
5.5
6.0
6.5
7.0
CT
CT + trastuzumab
Treatment

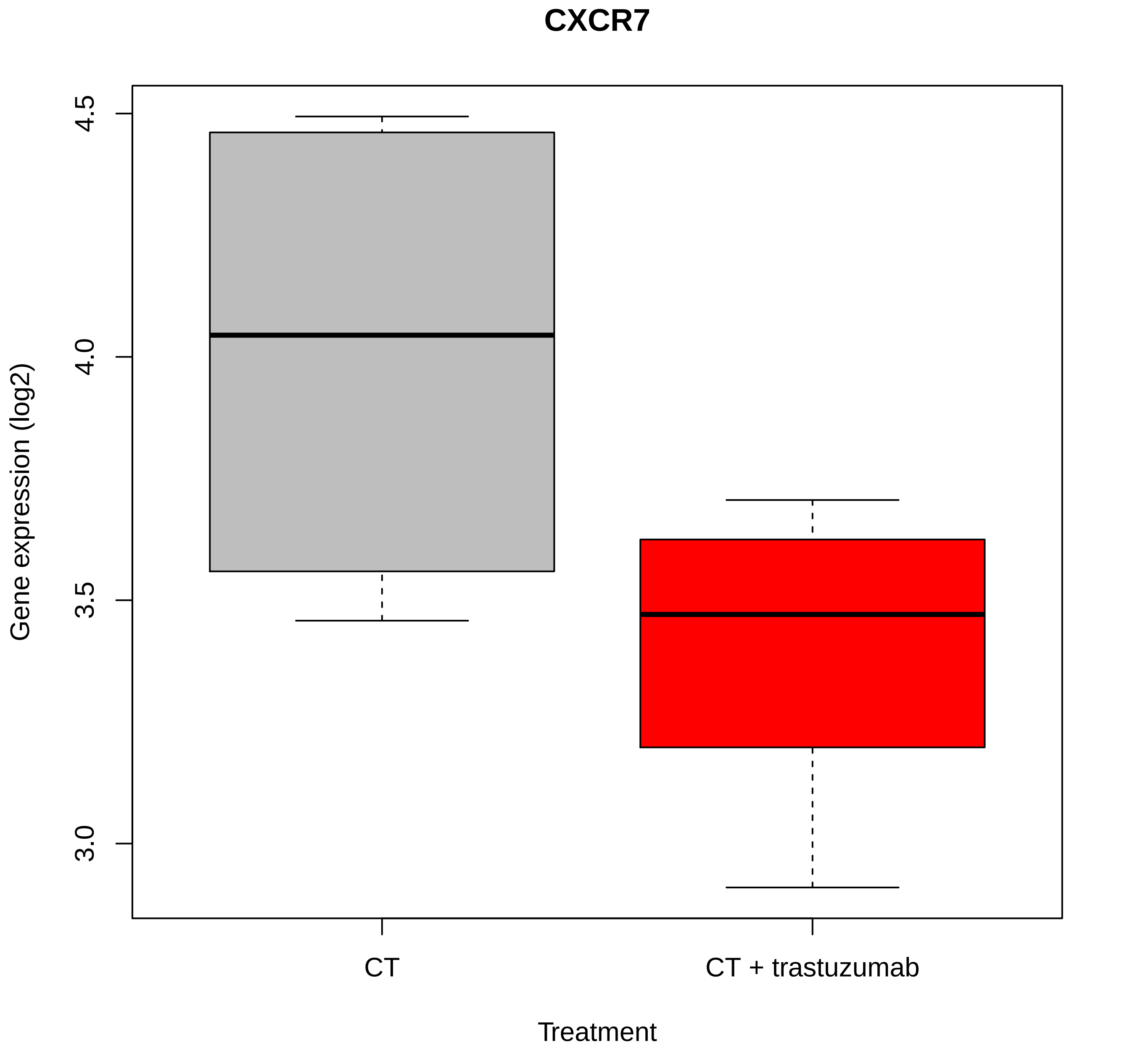
CXCR7
Gene expression (log2)
4.5
4.0
3.5
3.0
CT
CT + trastuzumab
Treatment

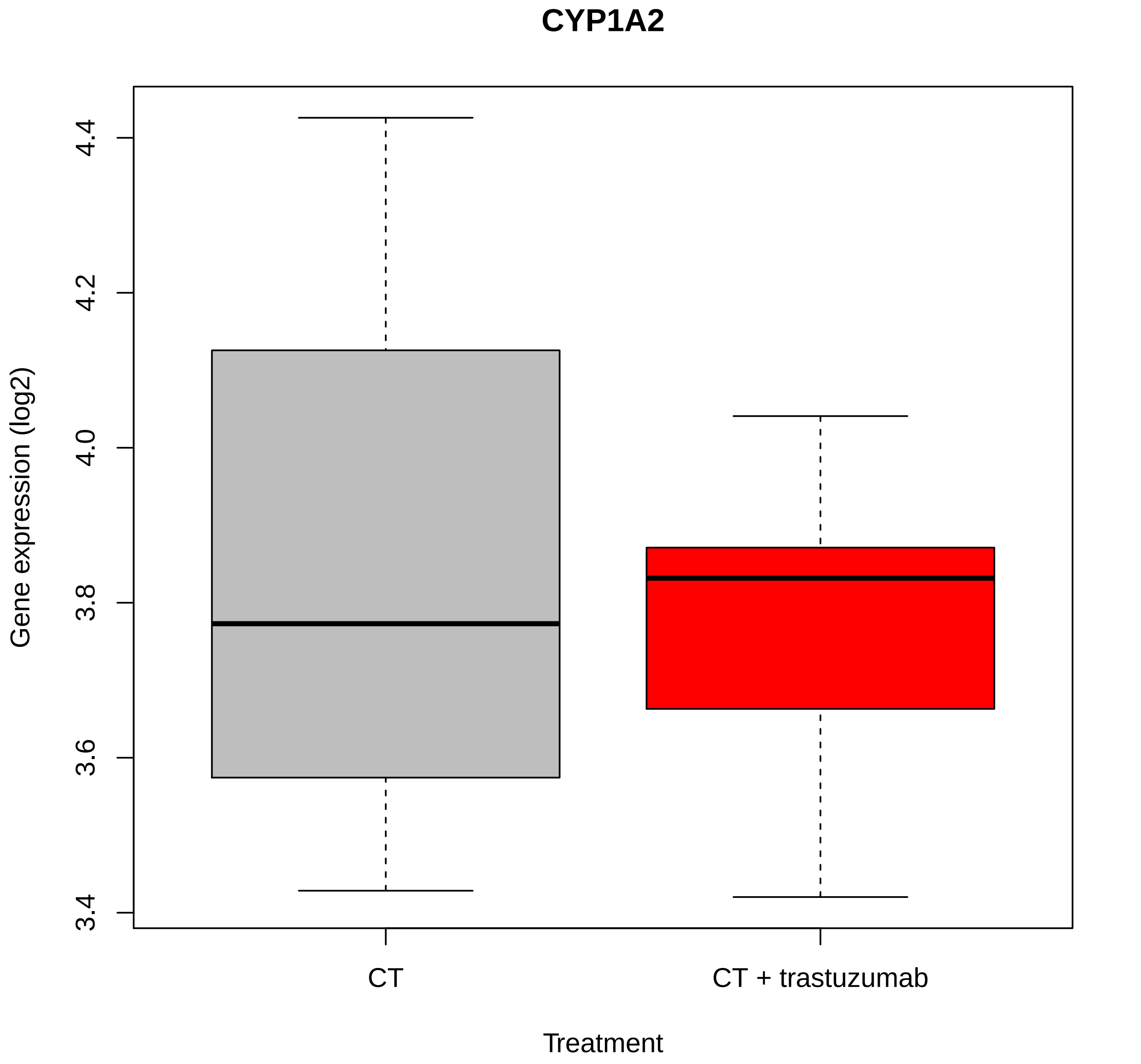
CYP1A2
Gene expression (log2)
4.4
4.2
4.0
3.8
3.6
3.4
CT
CT + trastuzumab
Treatment

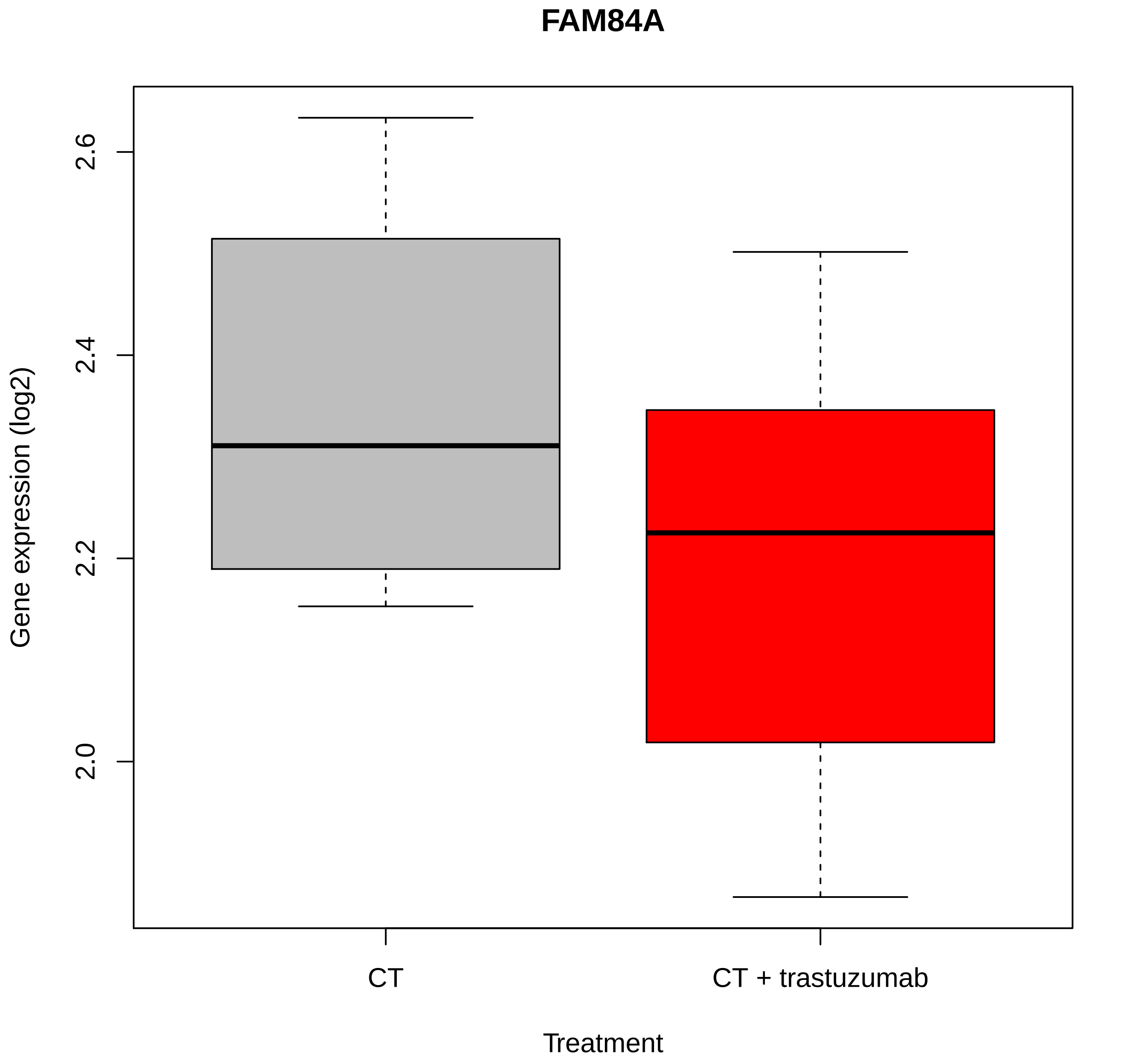
FAM84A
Gene expression (log2)
2.0
2.2
2.4
2.6
CT
CT + trastuzumab
Treatment

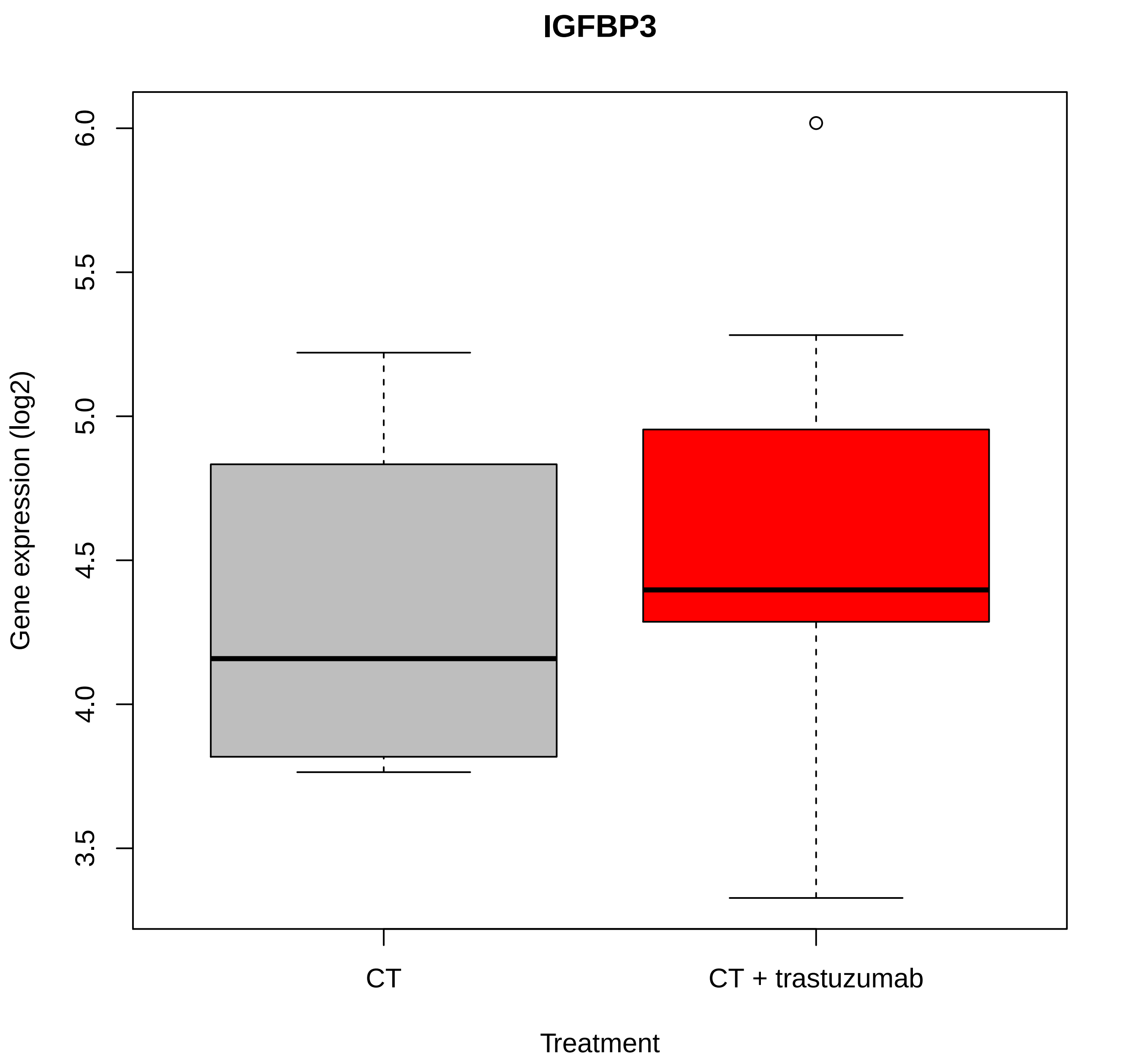
IGFBP3
Gene expression (log2)
6.0
5.5
5.0
4.5
4.0
3.5
CT
CT + trastuzumab
Treatment

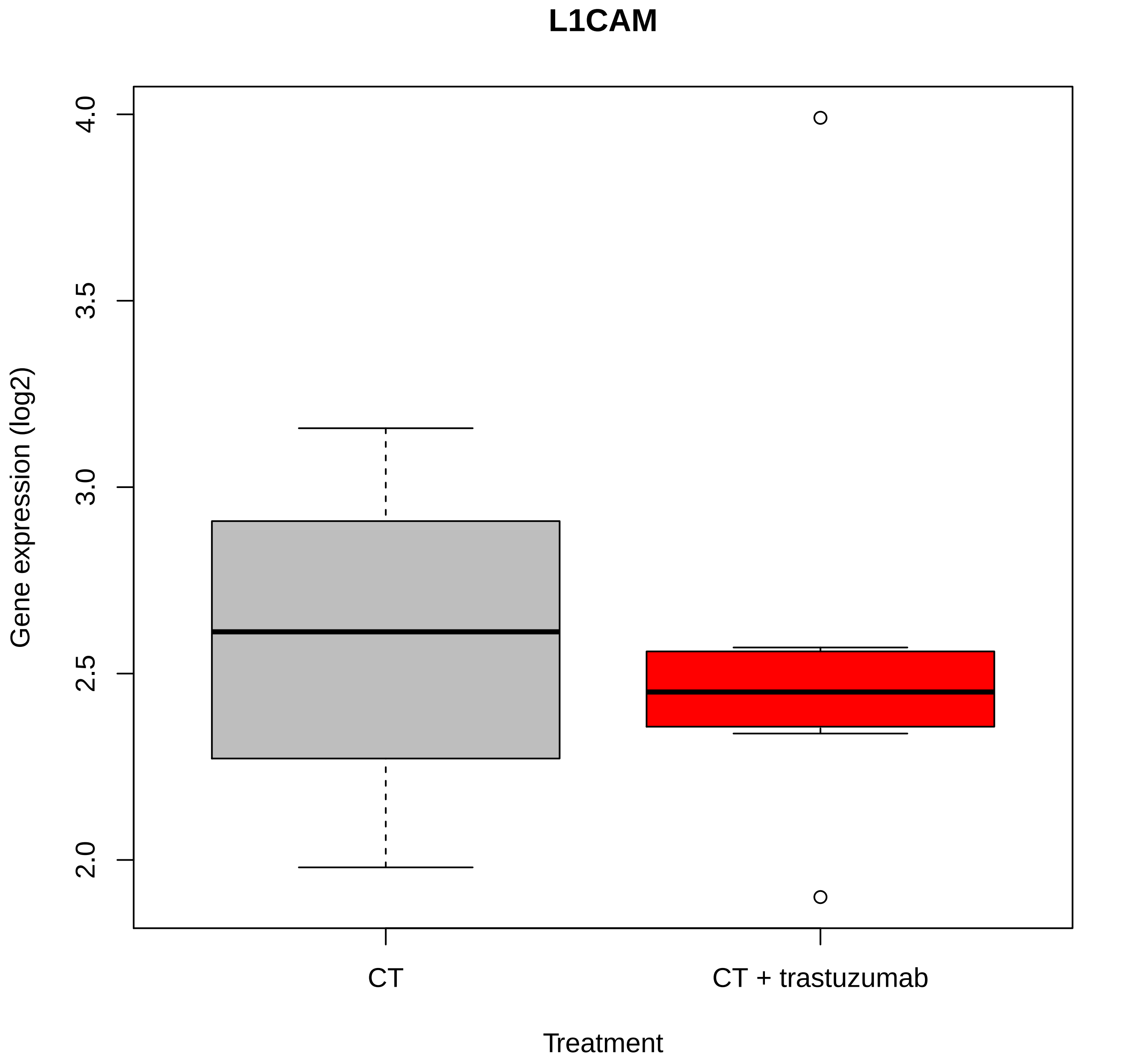
L1CAM
Gene expression (log2)
2.0
2.5
3.0
3.5
4.0
CT
CT + trastuzumab
Treatment

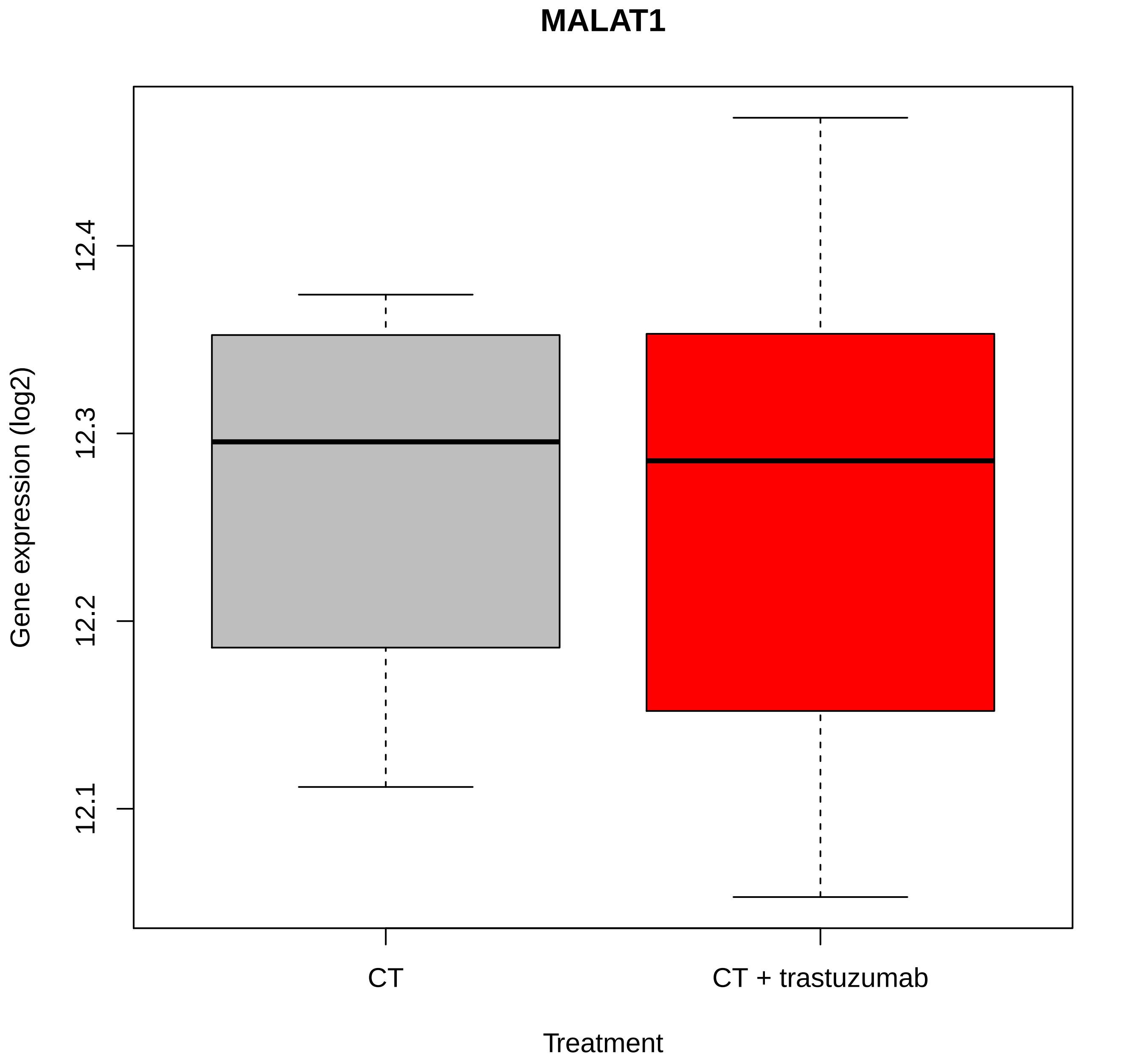
MALAT1
Gene expression (log2)
12.1
12.2
12.3
12.4
CT
CT + trastuzumab
Treatment

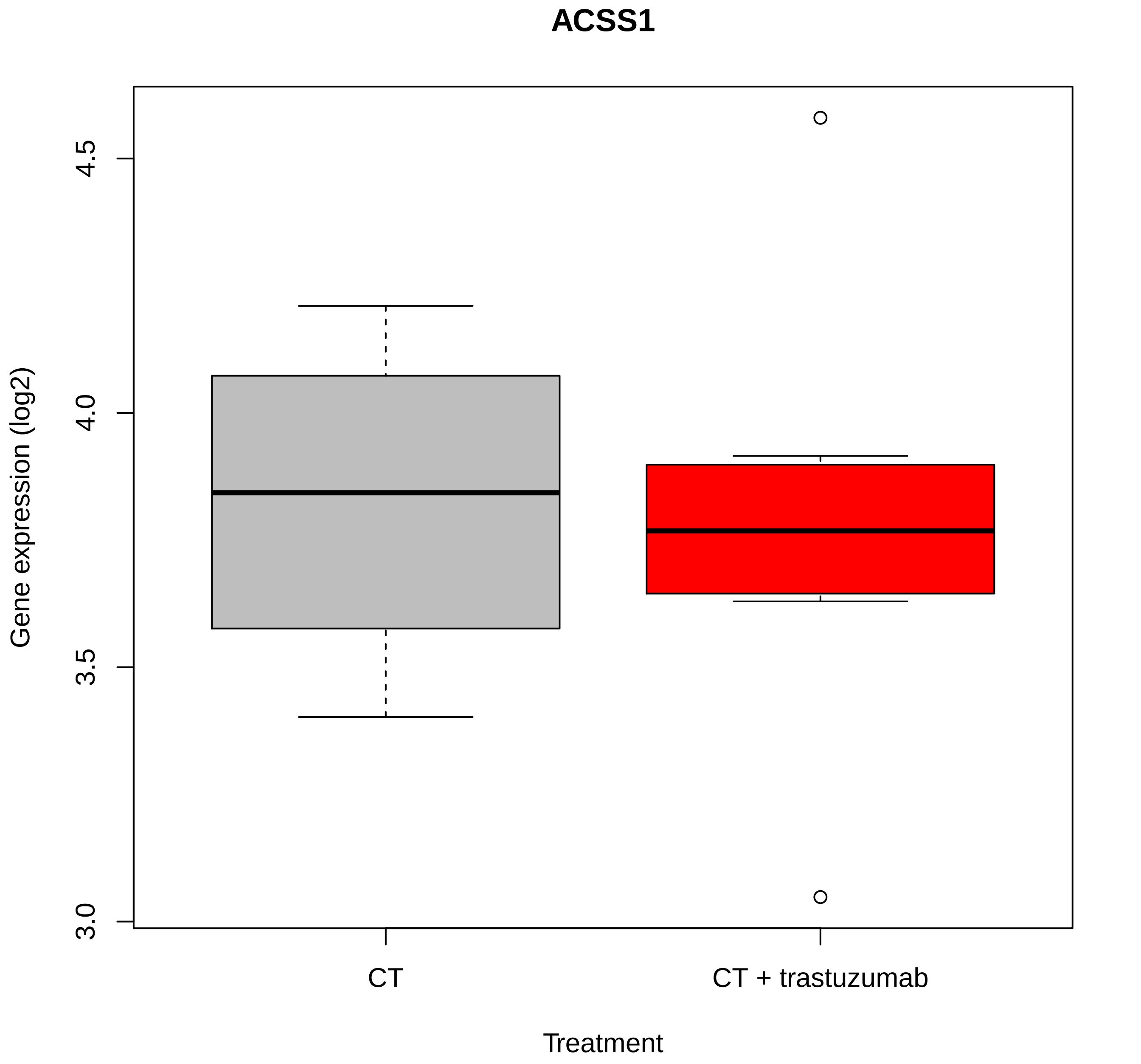
ACSS1
Gene expression (log2)
4.5
4.0
3.5
3.0
CT
CT + trastuzumab
Treatment

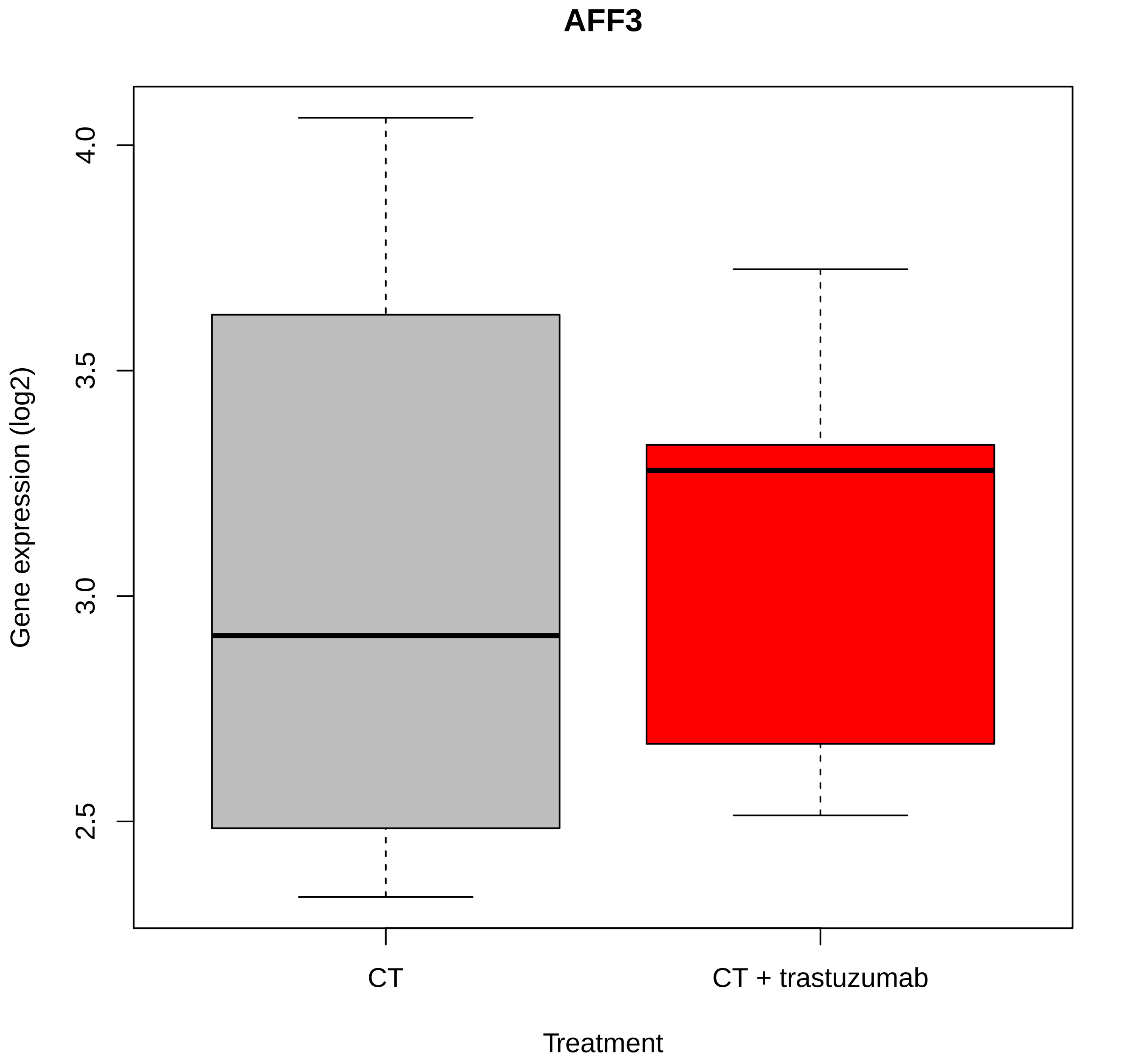
AFF3
Gene expression (log2)
4.0
3.5
3.0
2.5
CT
CT + trastuzumab
Treatment

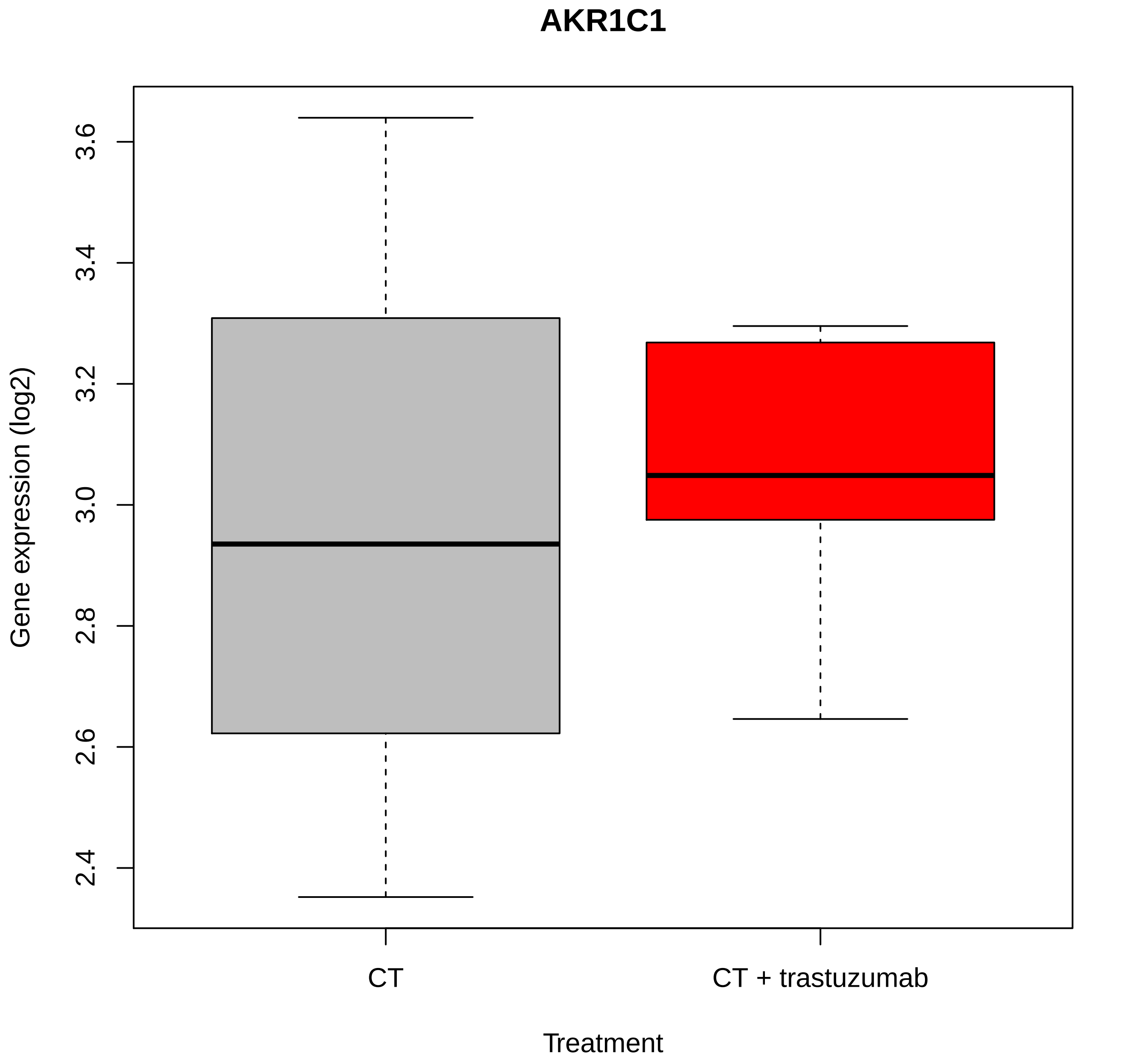
AKR1C1
Gene expression (log2)
3.6
3.4
3.2
3.0
2.8
2.6
2.4
CT
CT + trastuzumab
Treatment

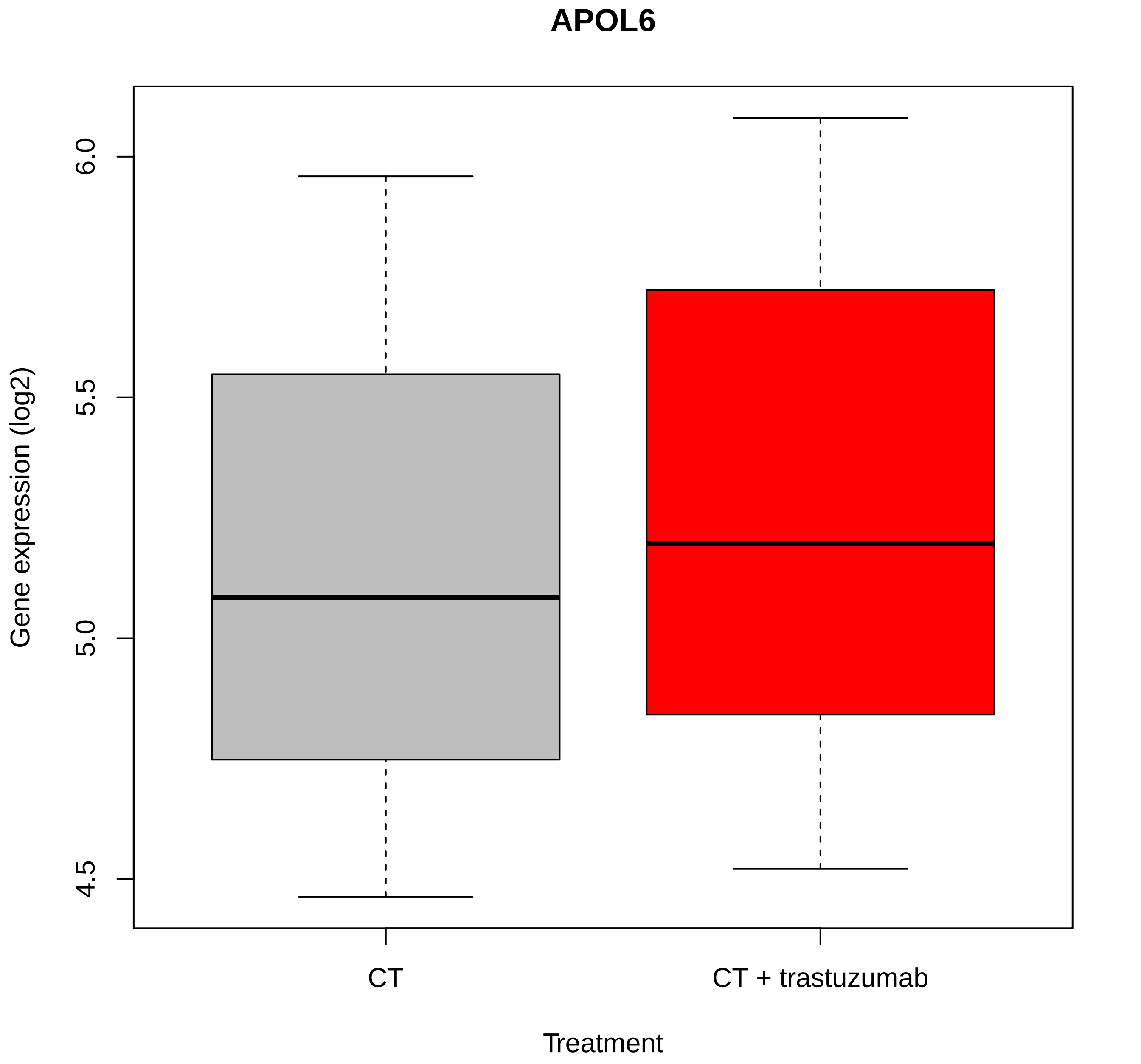

APOL6
Gene expression (log2)
6.0
5.5
5.0
4.5
CT
CT + trastuzumab
Treatment

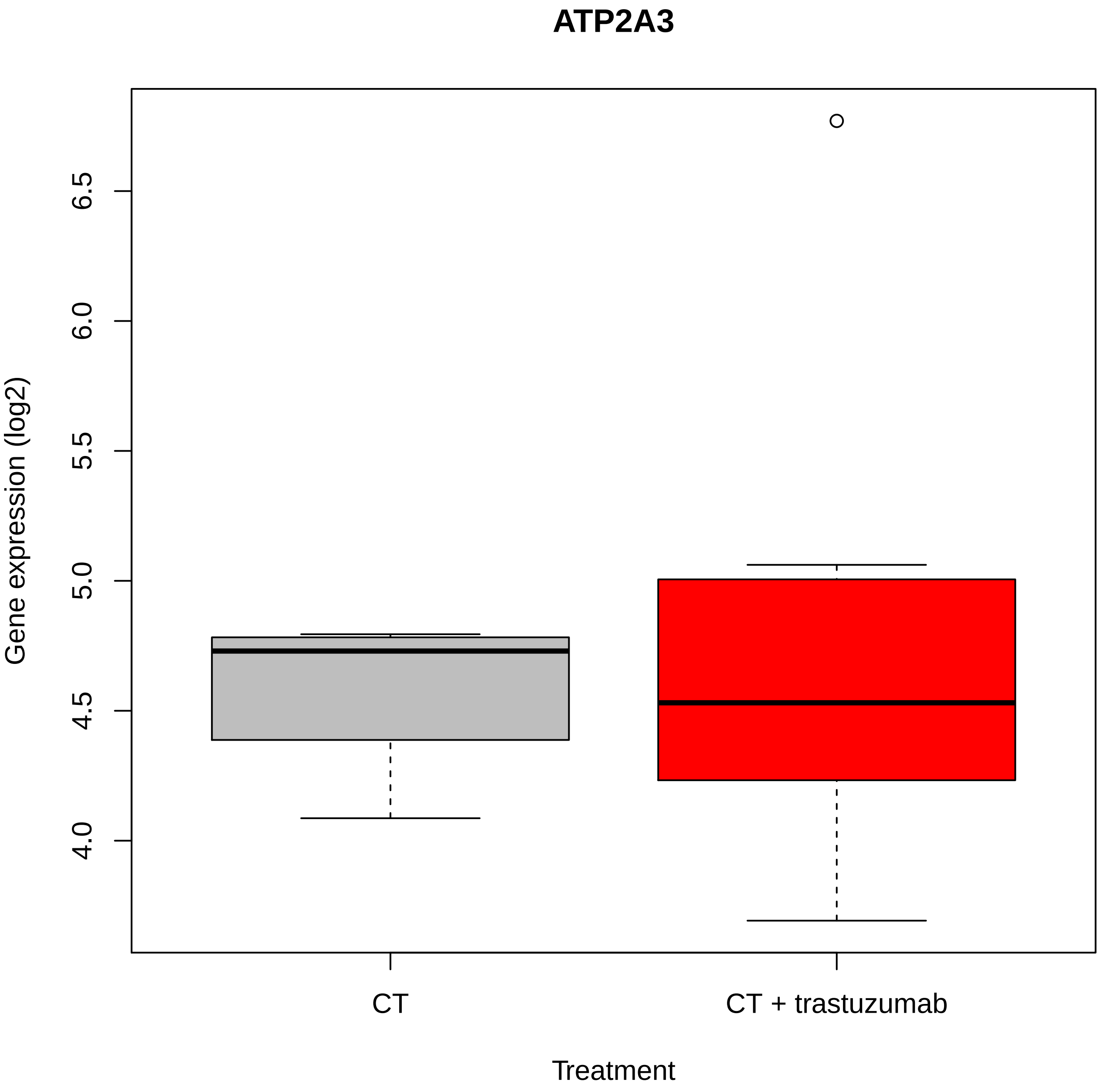
ATP2A3
Gene expression (log2)
6.5
6.0
5.5
5.0
4.5
4.0
CT
CT + trastuzumab
Treatment

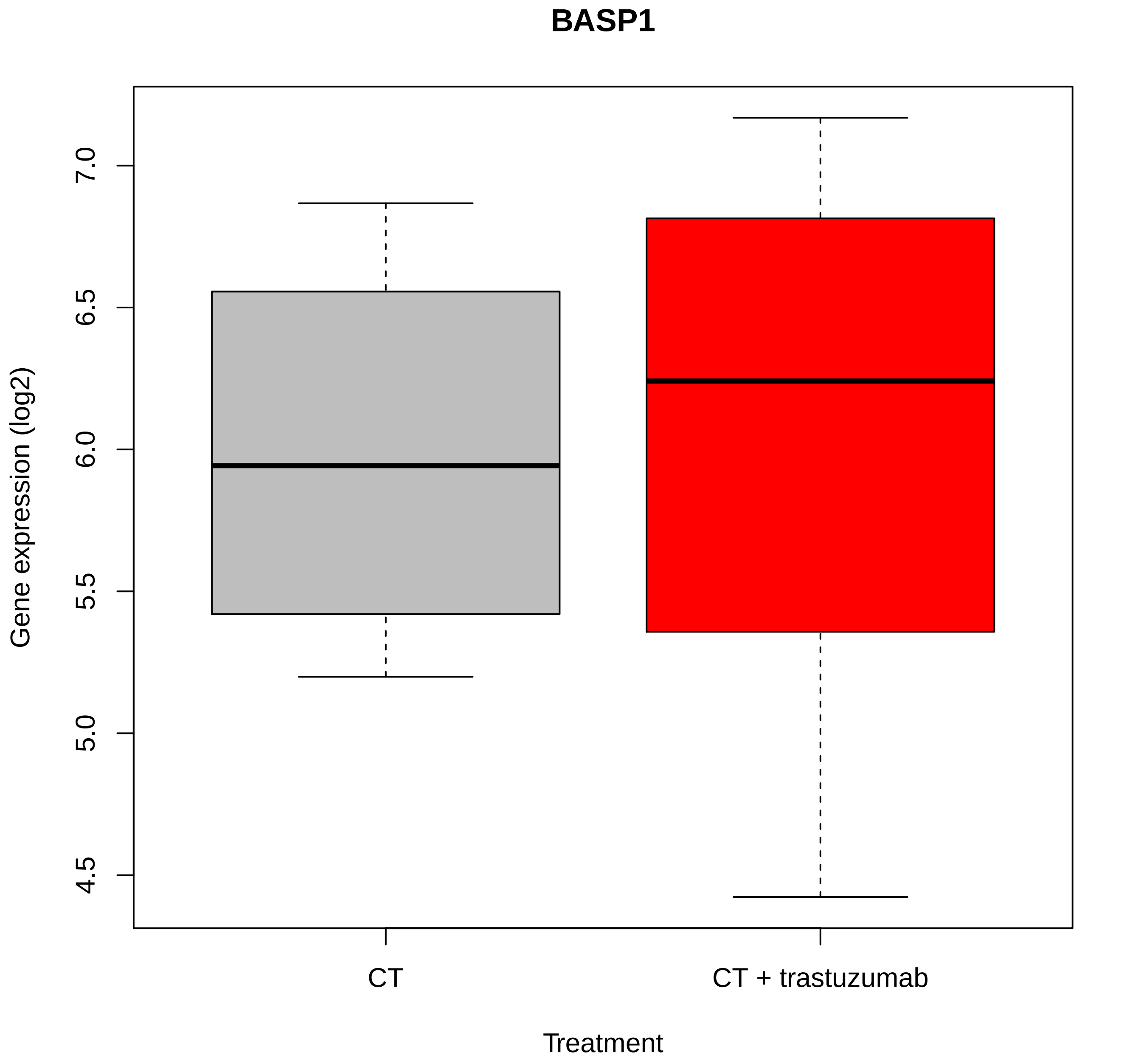
BASP1
Gene expression (log2)
7.0
6.5
6.0
5.5
5.0
4.5
CT
CT + trastuzumab
Treatment

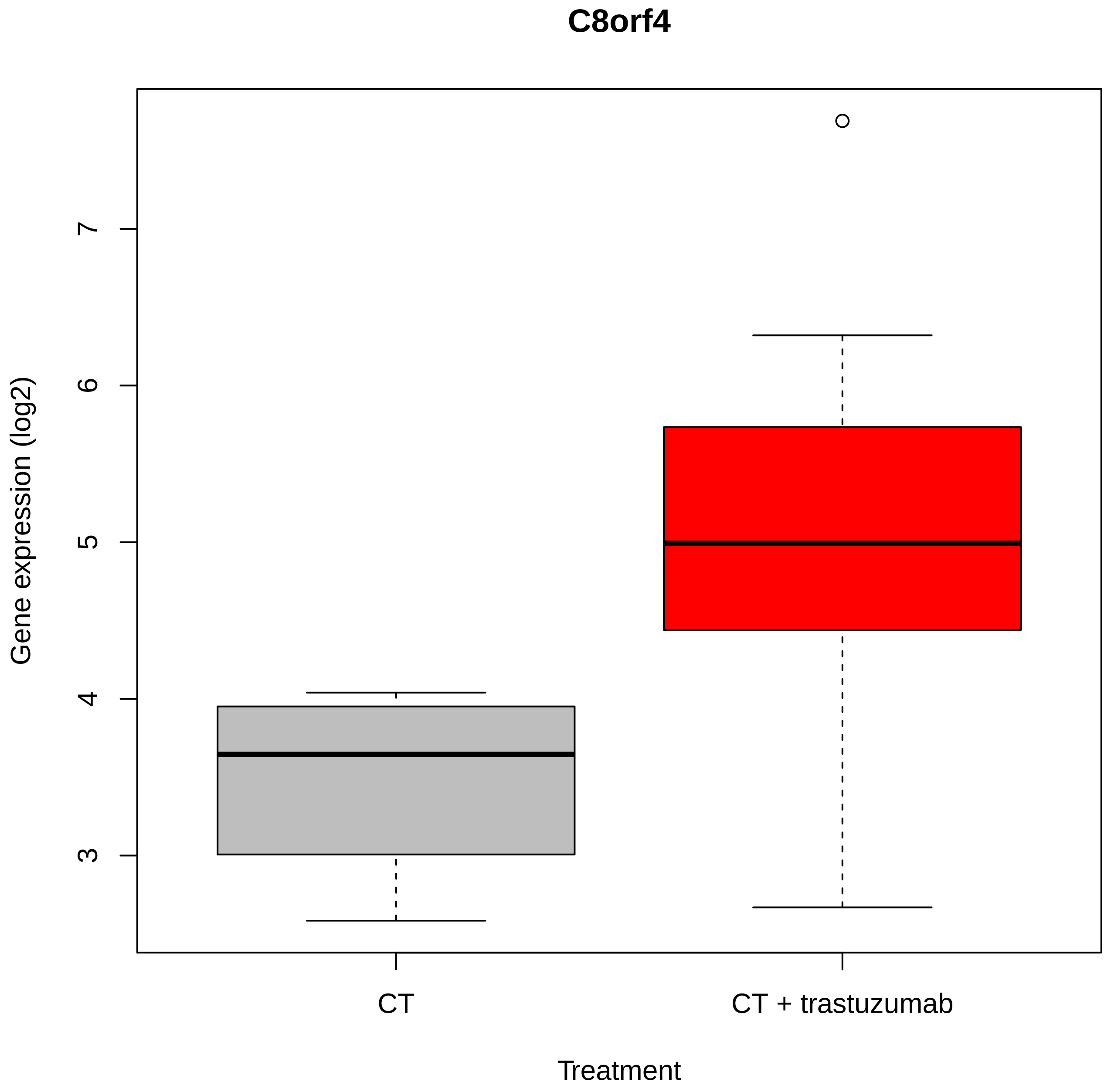
C8orf4
Gene expression (log2)
3
4
5
6
7
CT
CT + trastuzumab
Treatment

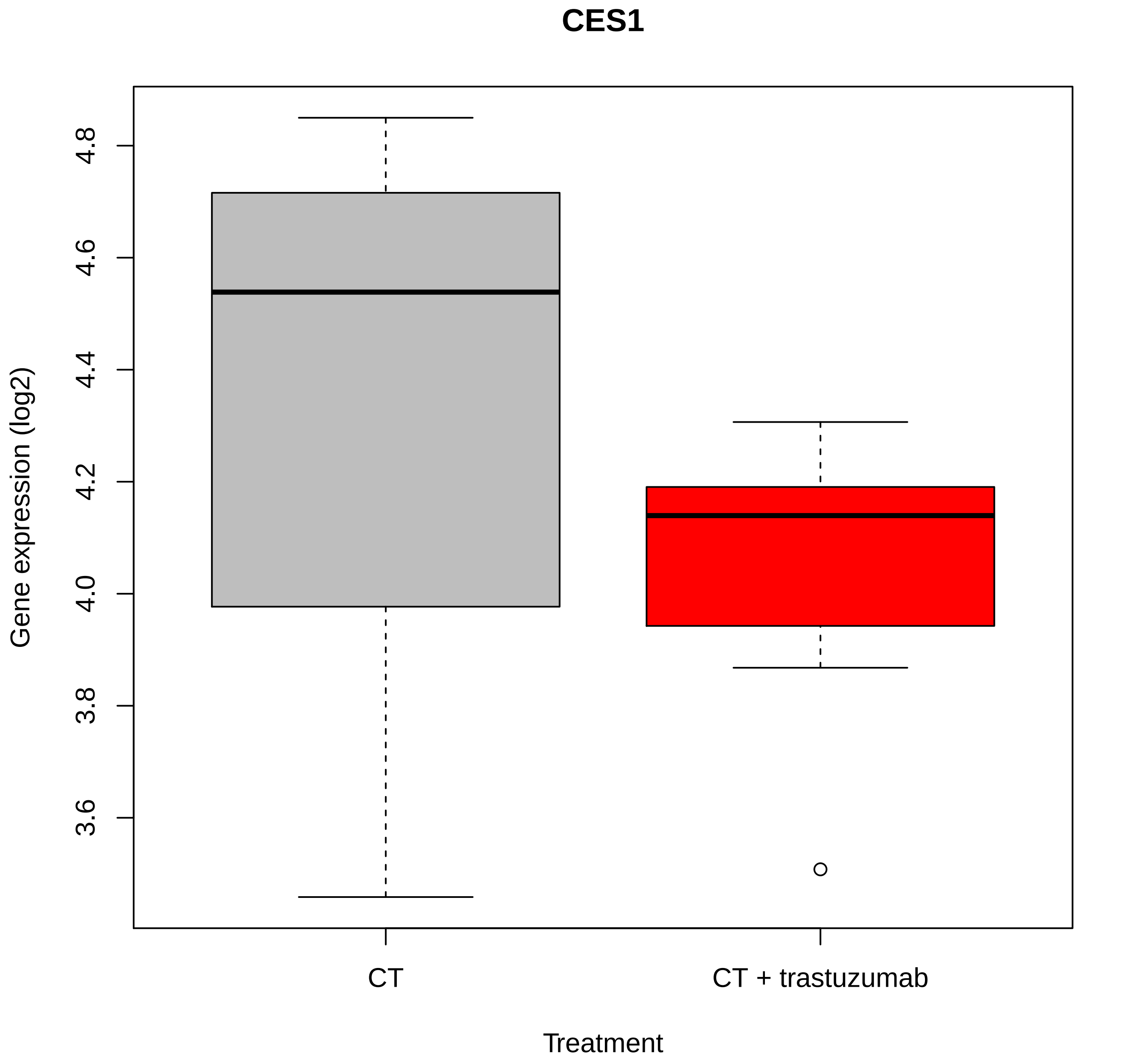

CES1
Gene expression (log2)
3.6
3.8
4.0
4.2
4.4
4.6
4.8
CT
CT + trastuzumab
Treatment

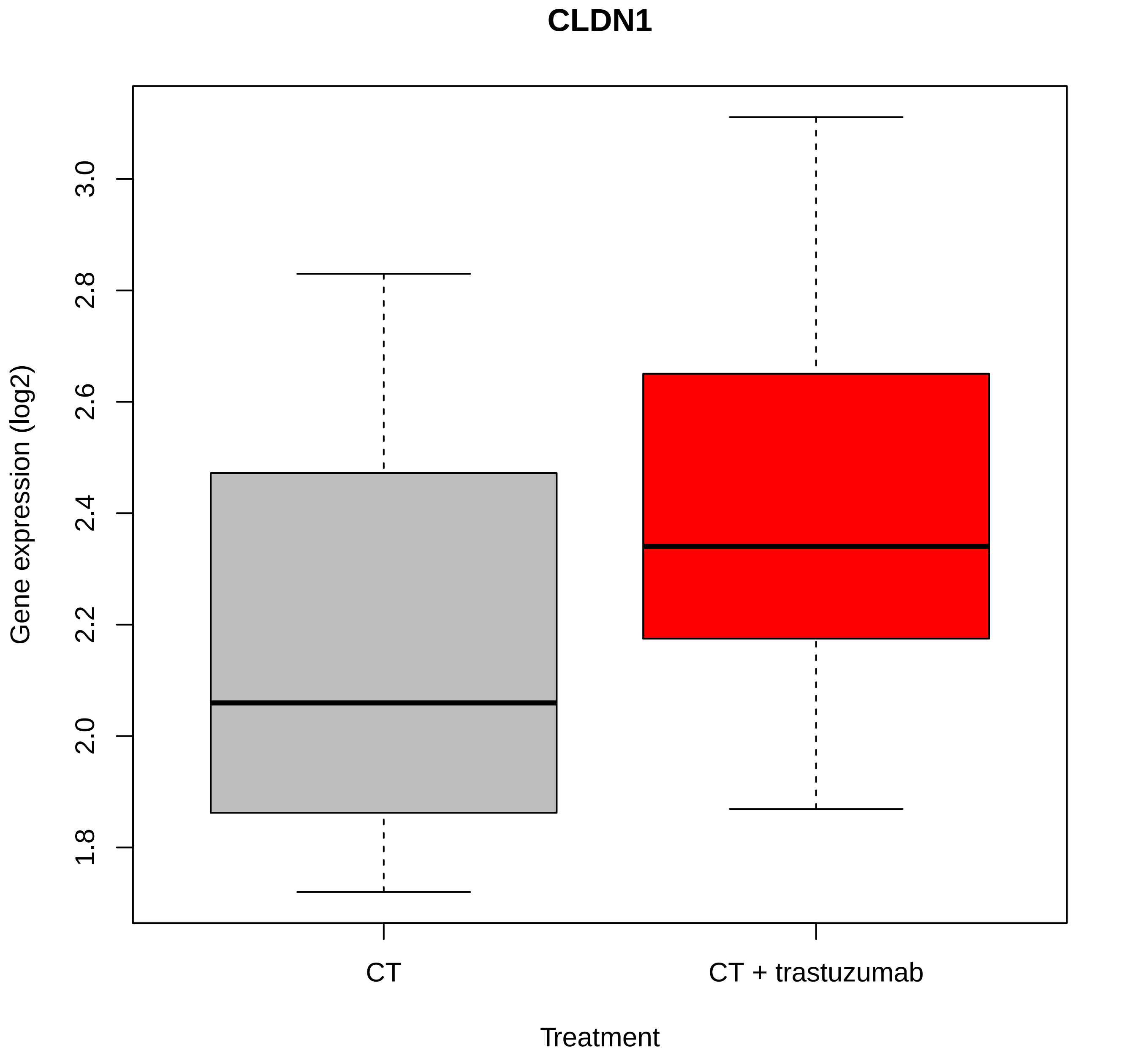
CLDN1
Gene expression (log2)
1.8
2.0
2.2
2.4
2.6
2.8
3.0
CT
CT + trastuzumab
Treatment

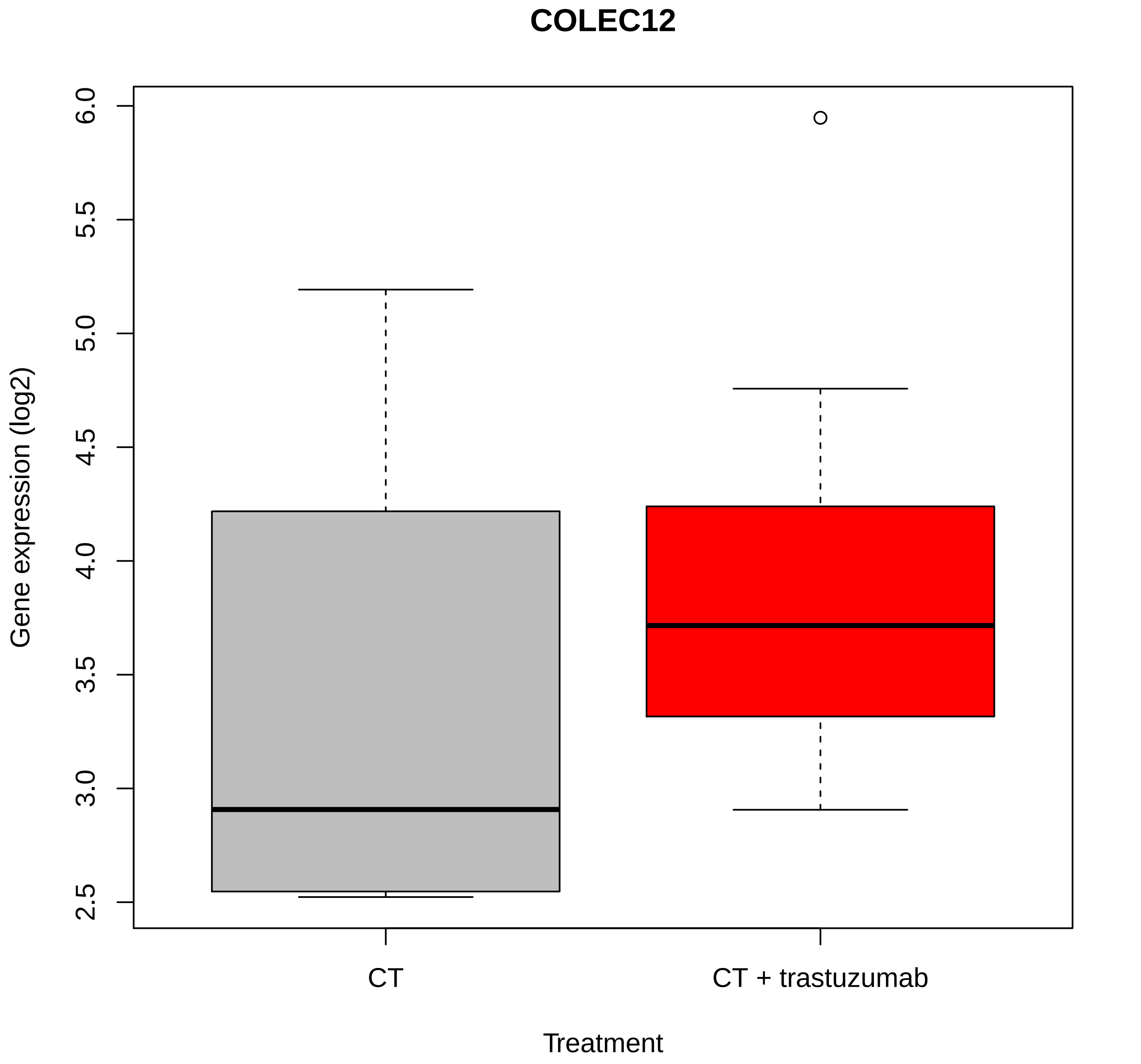
COLEC12
Gene expression (log2)
6.0
5.5
5.0
4.5
4.0
3.5
3.0
2.5
CT
CT + trastuzumab
Treatment

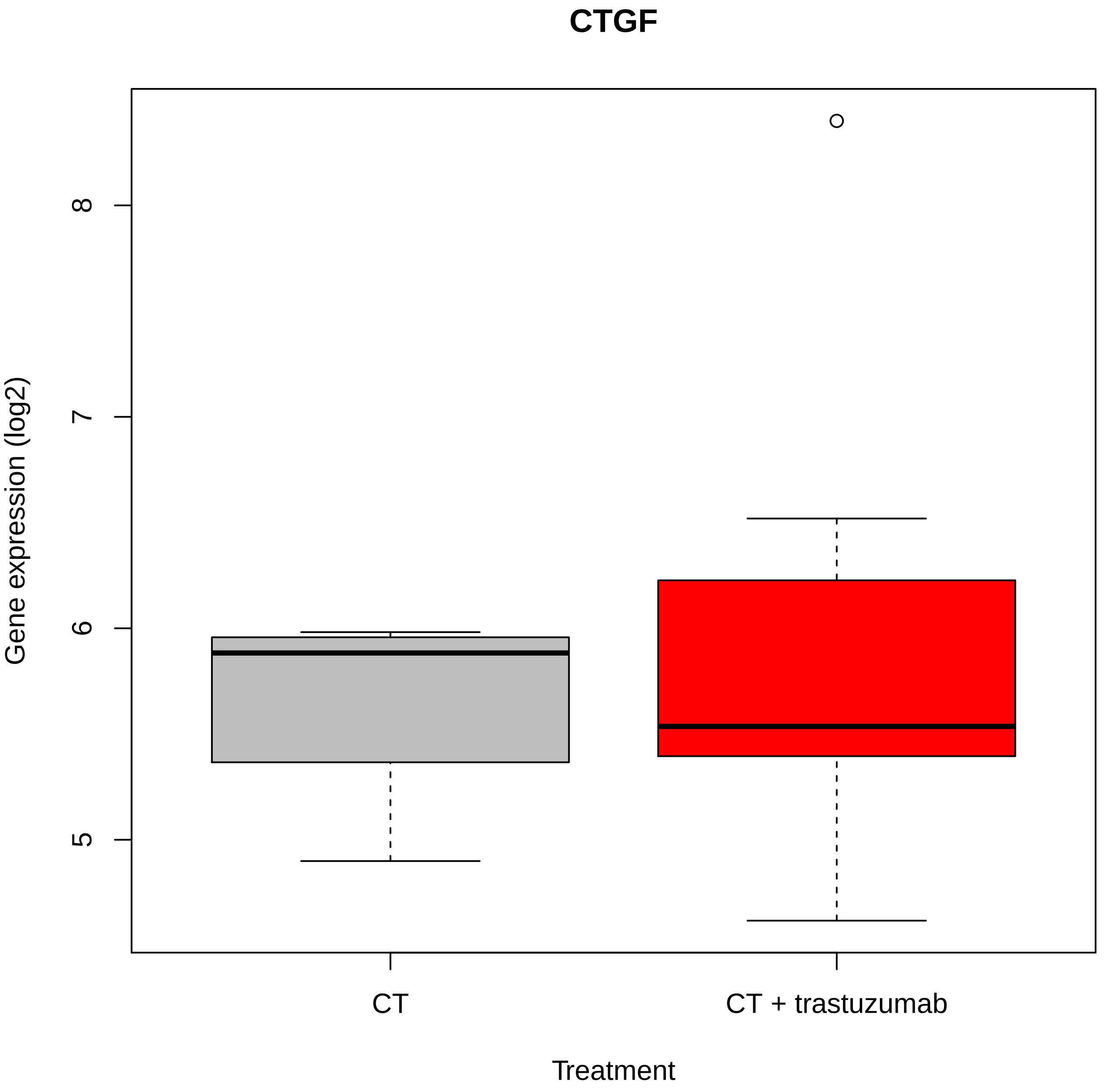
CTGF
Gene expression (log2)
8
7
6
5
CT
CT + trastuzumab
Treatment

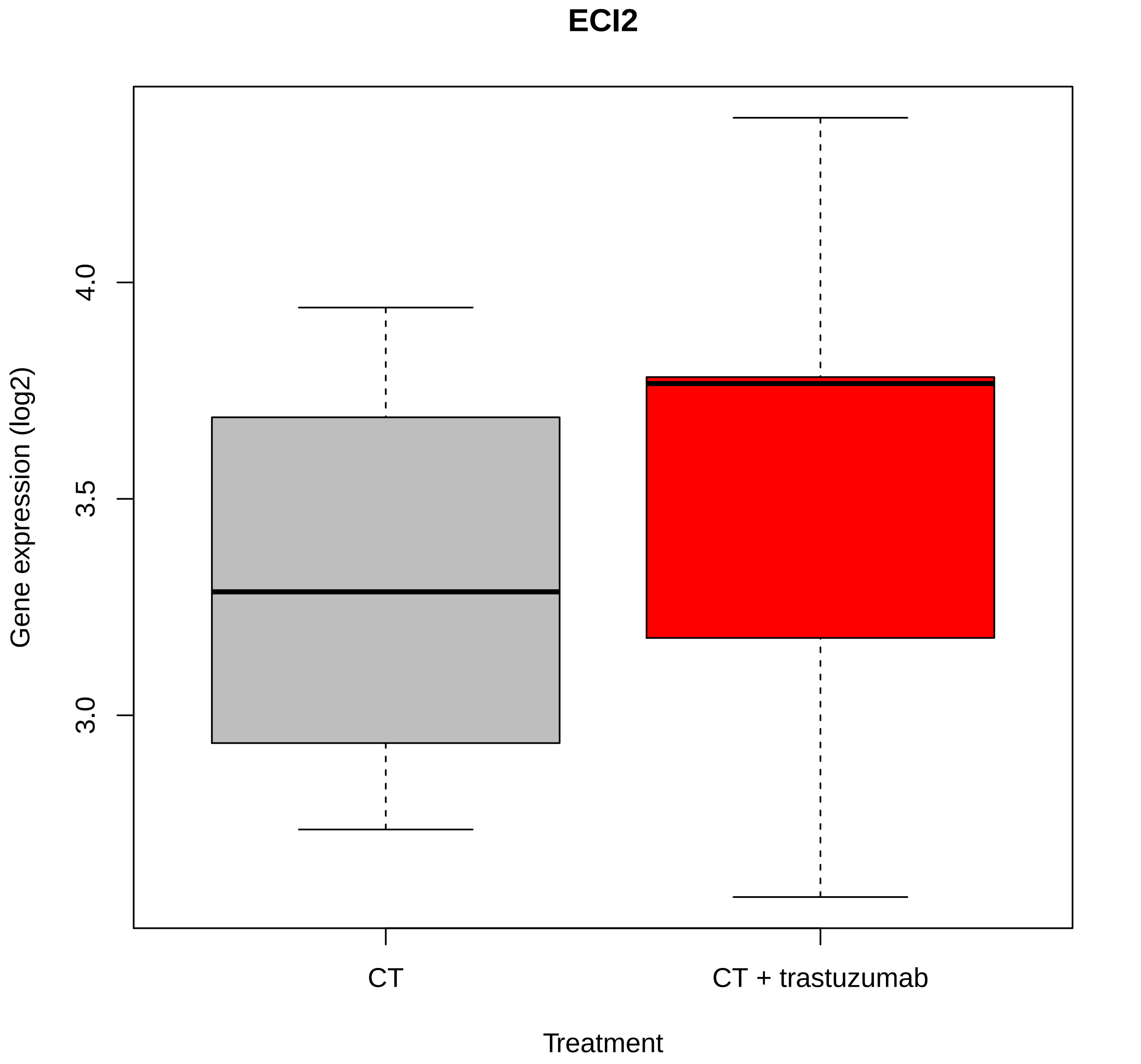

ECI2
Gene expression (log2)
3.0
3.5
4.0
CT
CT + trastuzumab
Treatment

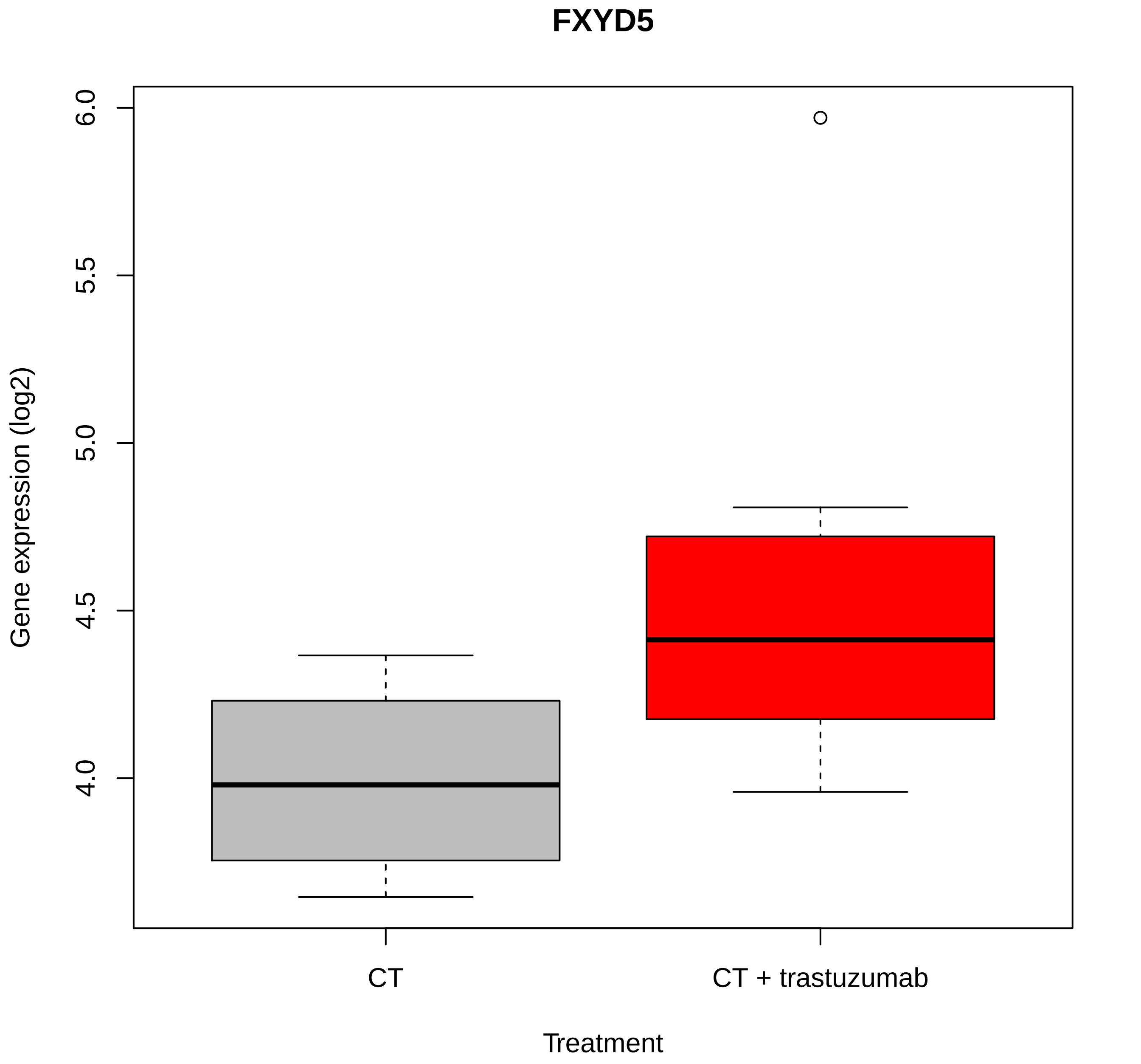
FXYD5
Gene expression (log2)
6.0
5.5
5.0
4.5
4.0
CT
CT + trastuzumab
Treatment

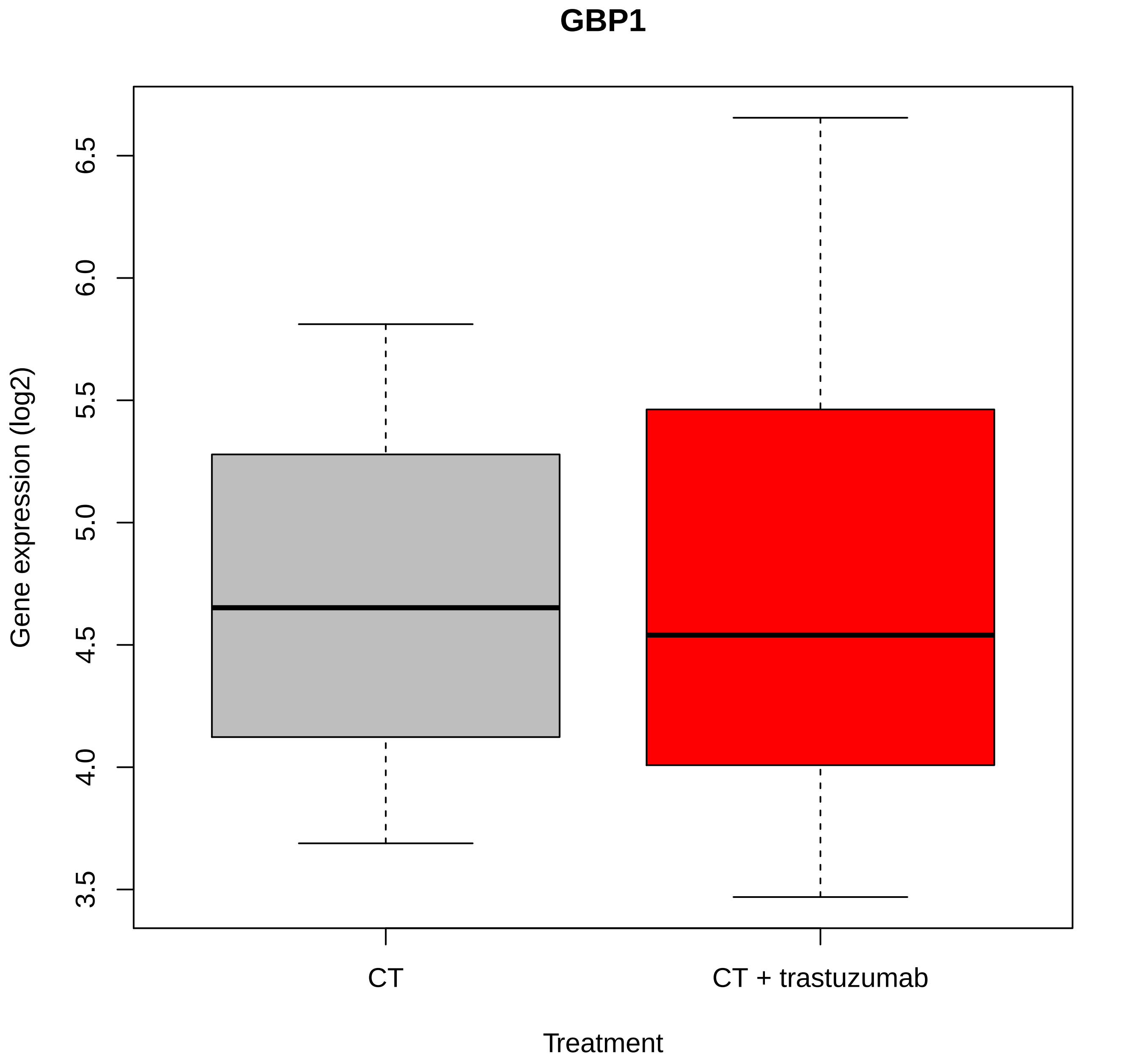
GBP1
Gene expression (log2)
6.5
6.0
5.5
5.0
4.5
4.0
3.5
CT
CT + trastuzumab
Treatment

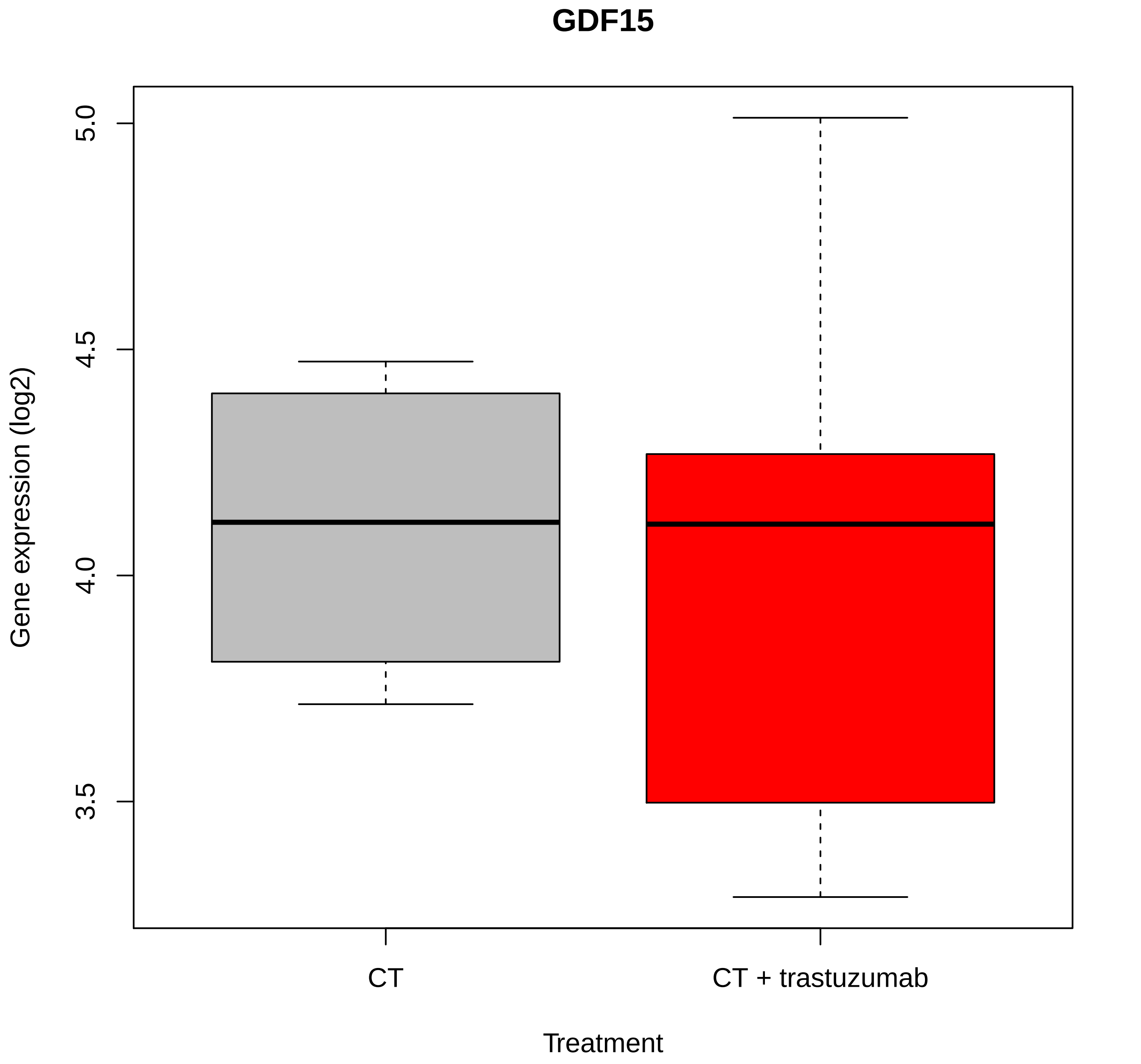
GDF15
Gene expression (log2)
5.0
4.5
4.0
3.5
CT
CT + trastuzumab
Treatment

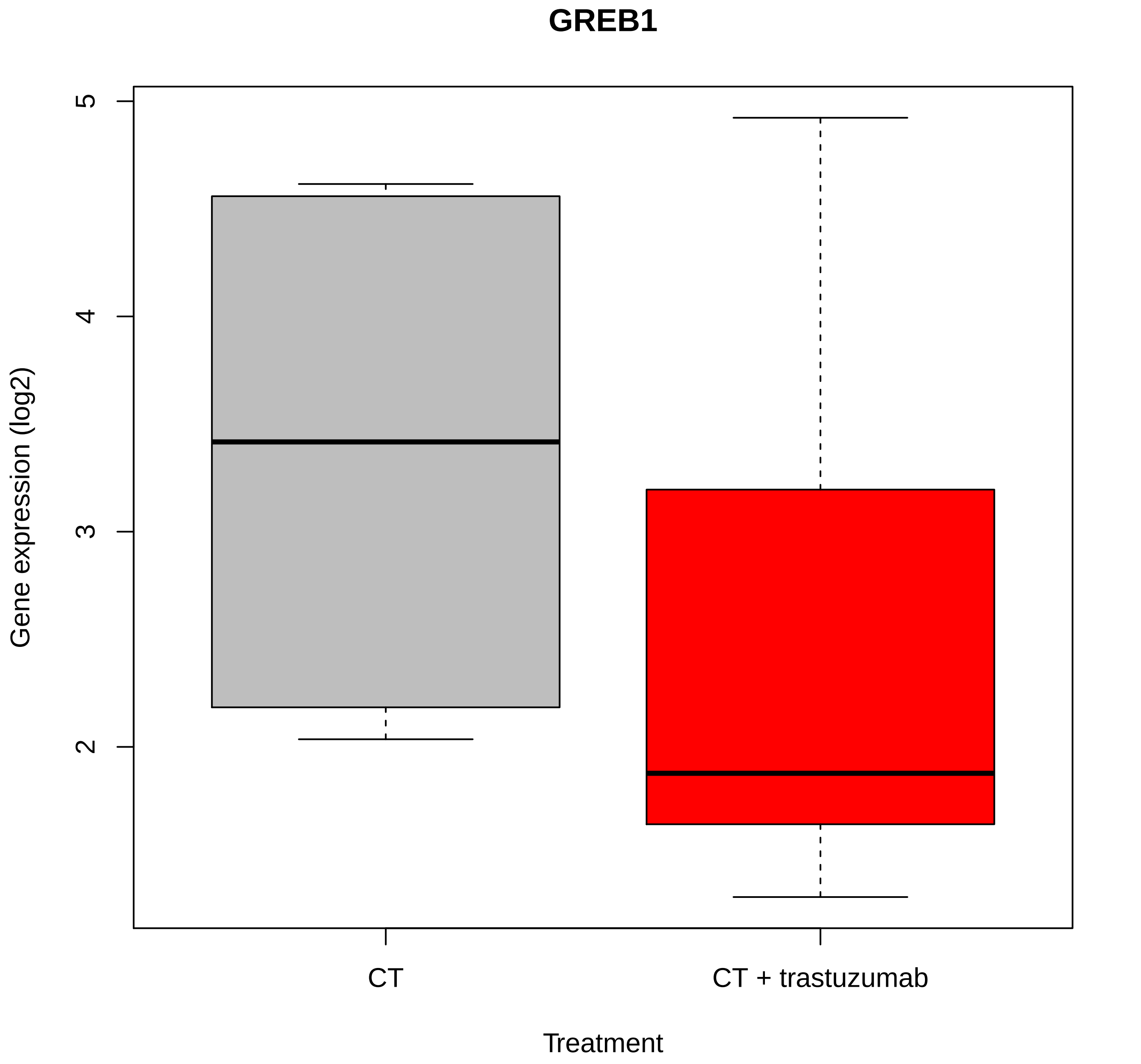
GREB1
Gene expression (log2)
2
3
4
5
CT
CT + trastuzumab
Treatment

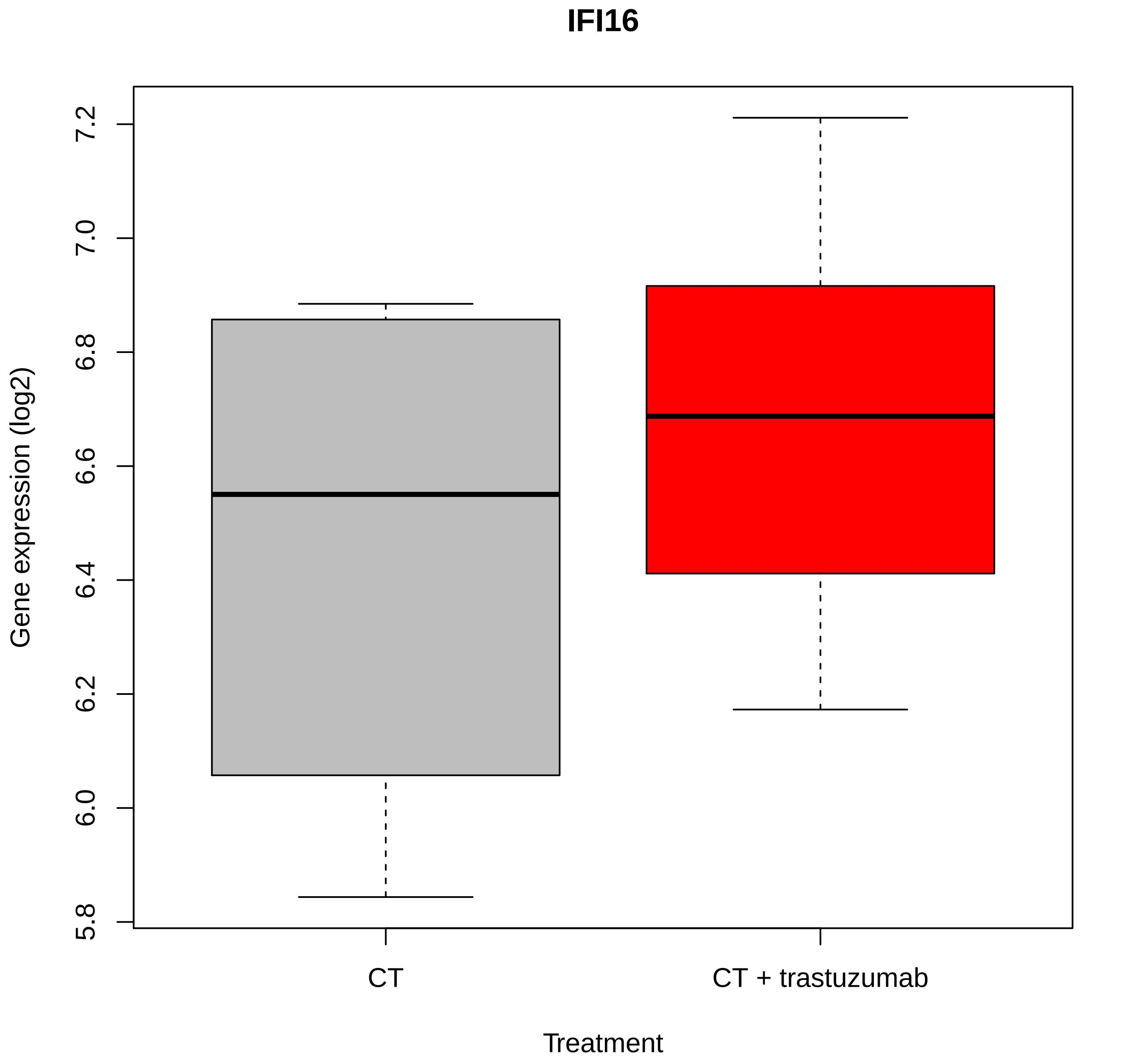
IFI16
Gene expression (log2)
7.2
7.0
6.8
6.6
6.4
6.2
6.0
5.8
CT
CT + trastuzumab
Treatment

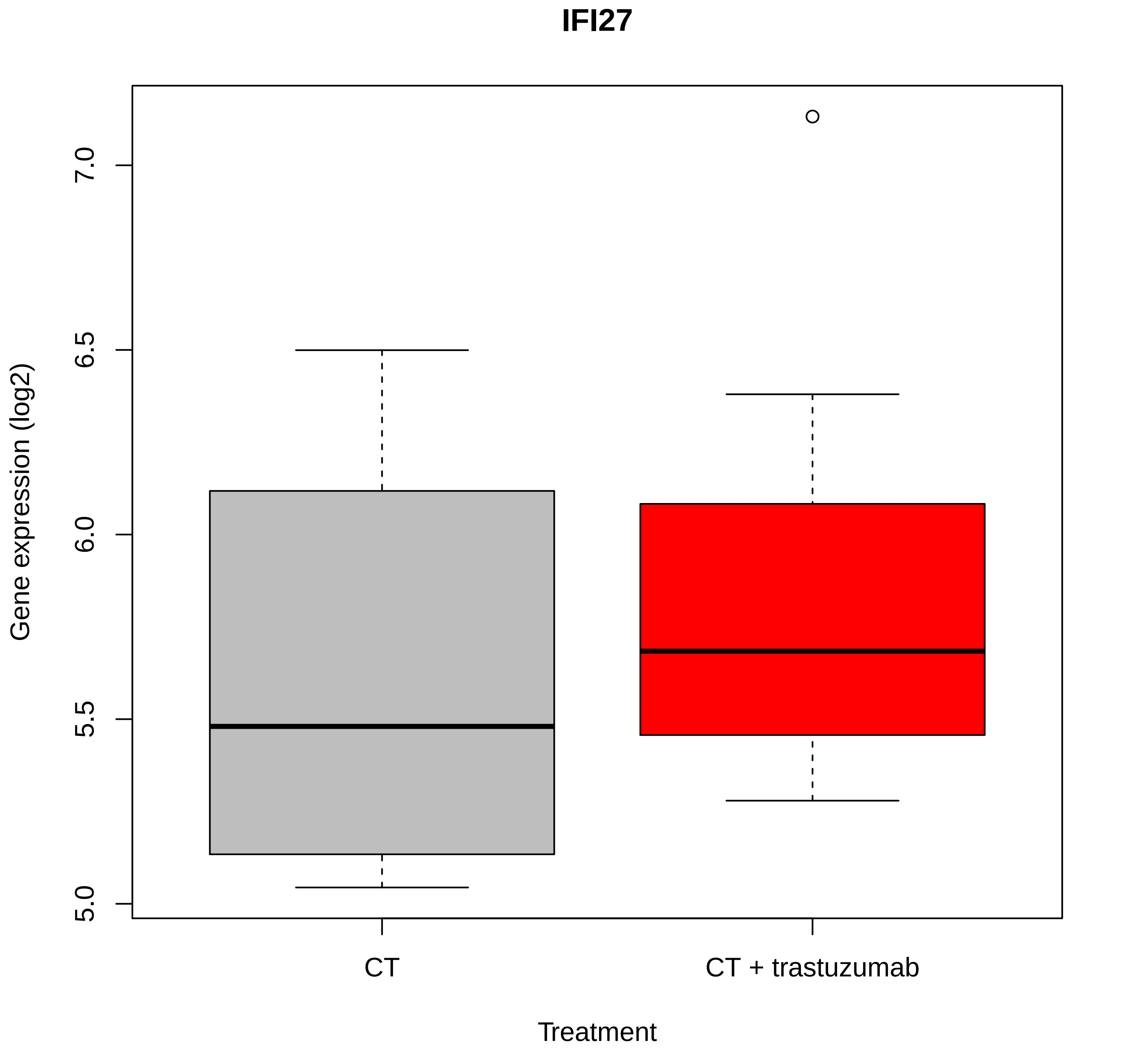
IFI27
Gene expression (log2)
5.0
5.5
6.0
6.5
7.0
CT
CT + trastuzumab
Treatment

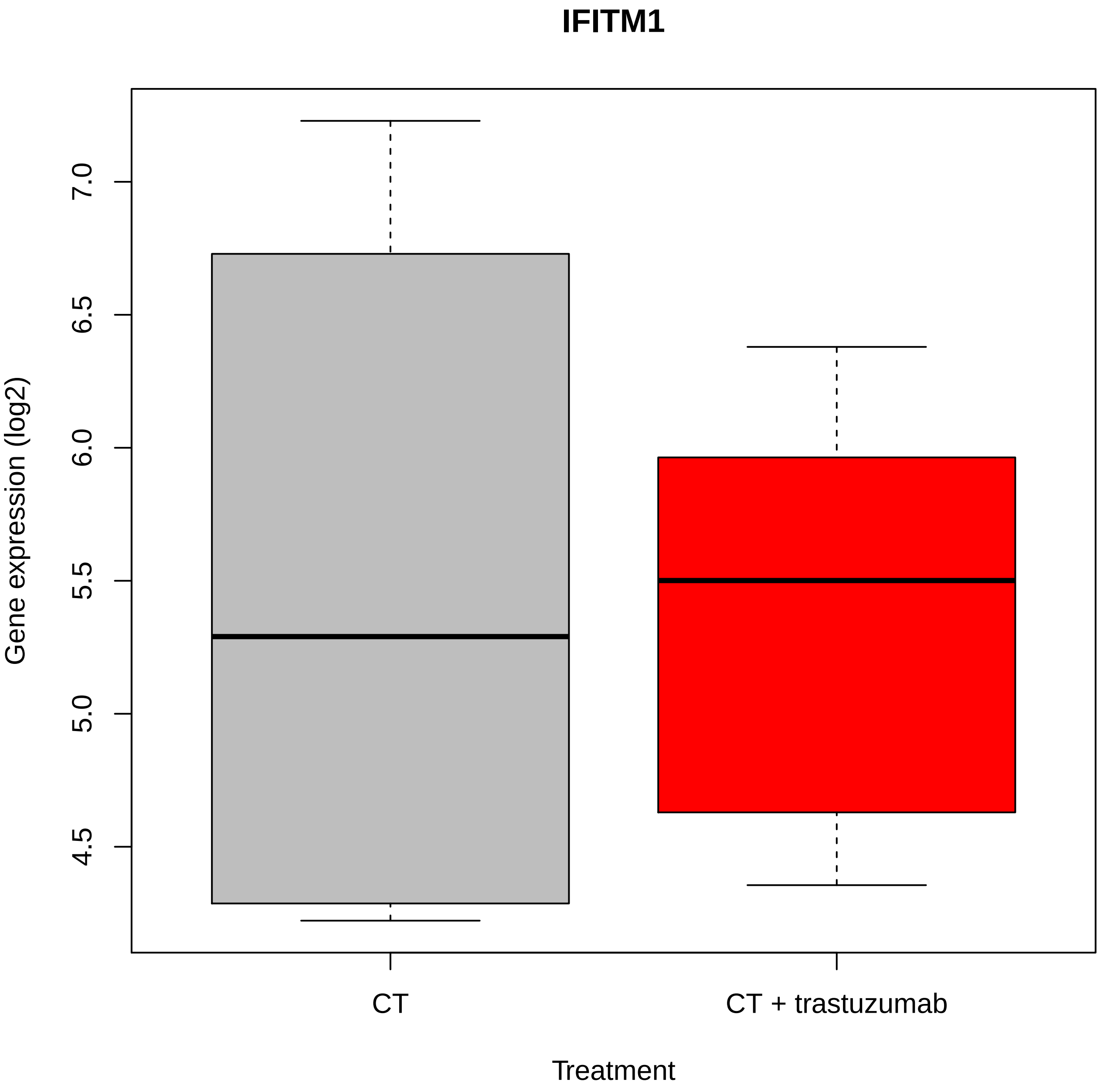

IFITM1
Gene expression (log2)
7.0
6.5
6.0
5.5
5.0
4.5
CT
CT + trastuzumab
Treatment

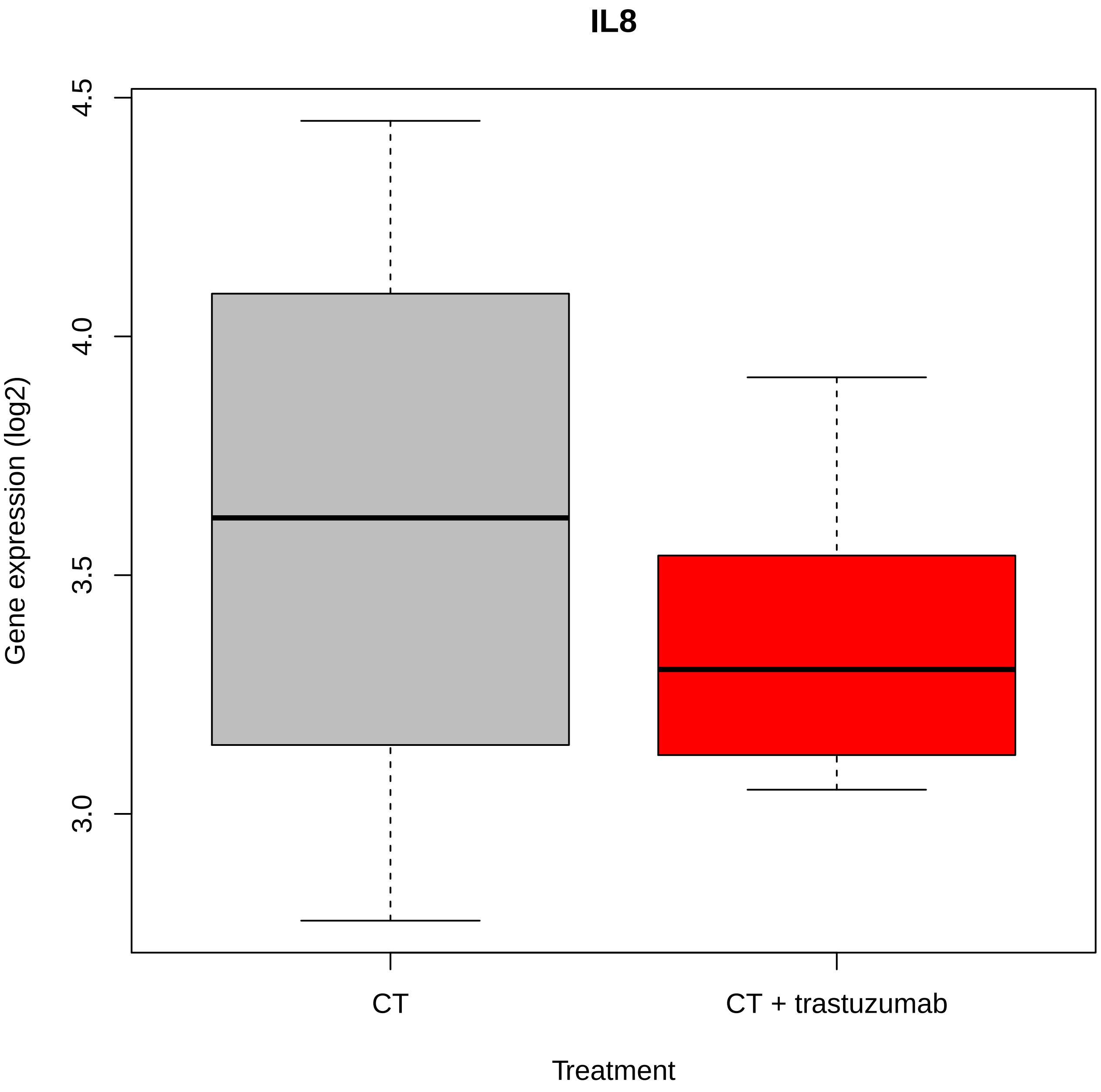
IL8
Gene expression (log2)
4.5
4.0
3.5
3.0
CT
CT + trastuzumab
Treatment

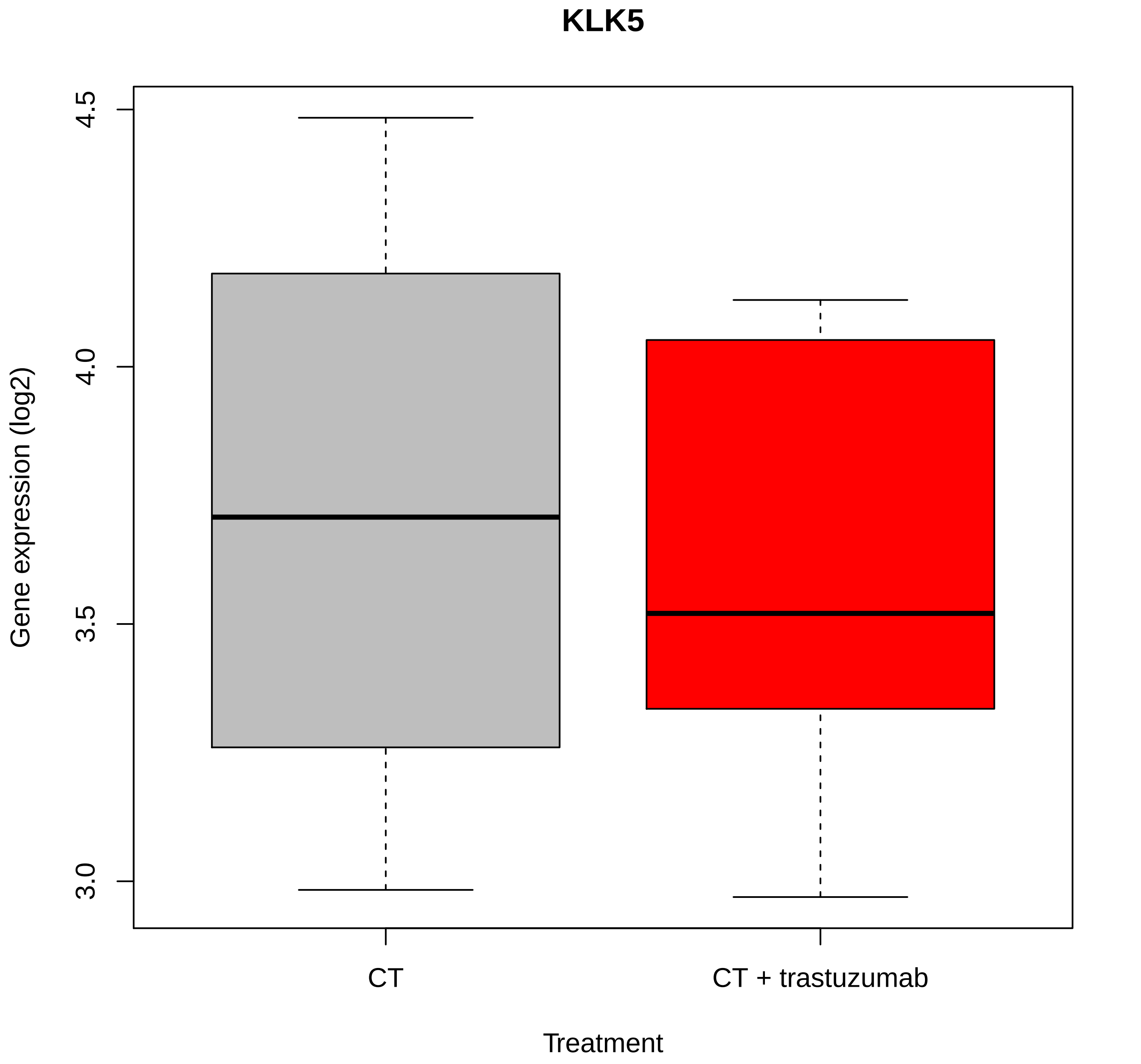
KLK5
Gene expression (log2)
4.5
4.0
3.5
3.0
CT
CT + trastuzumab
Treatment

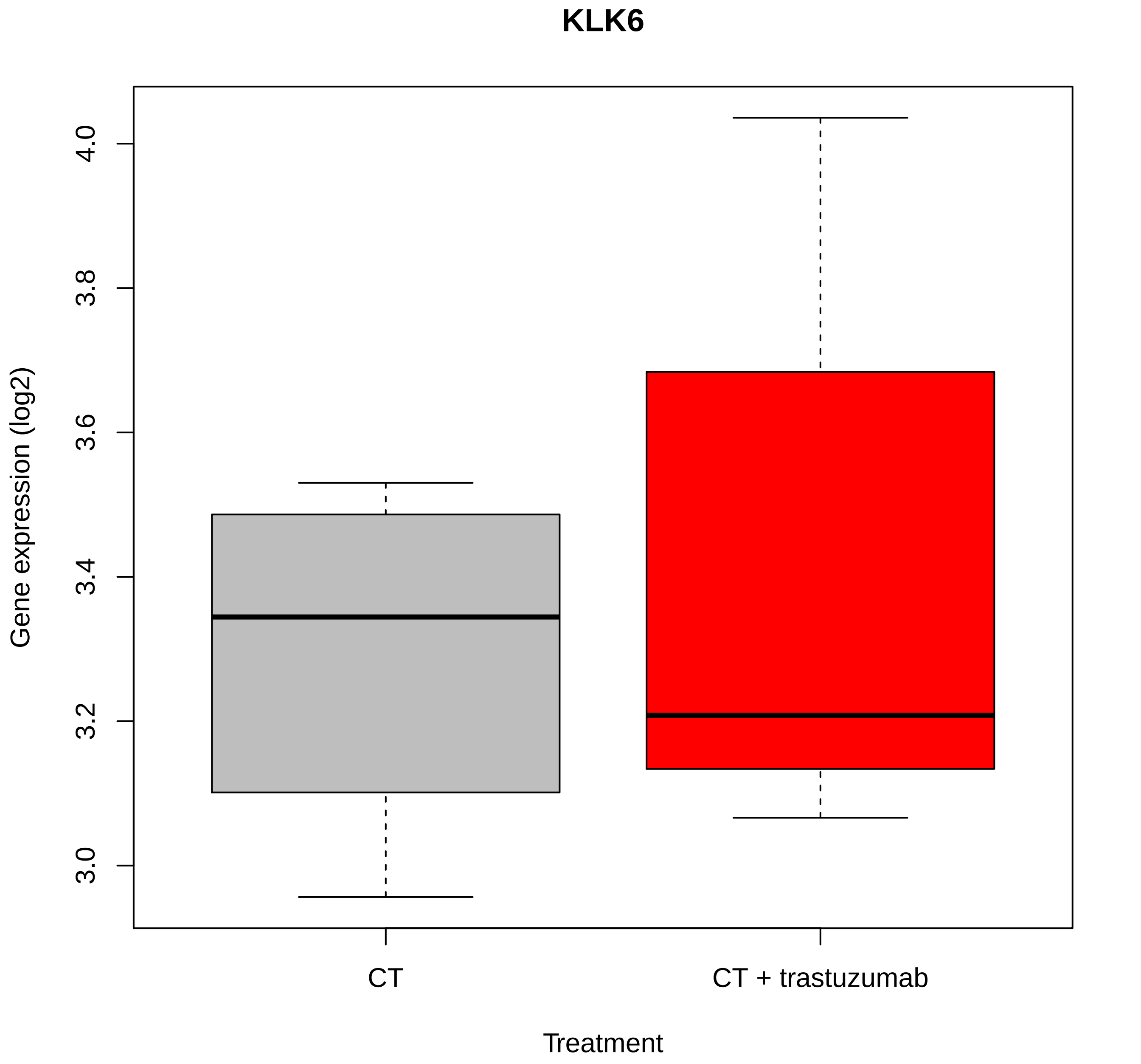
KLK6
Gene expression (log2)
3.0
3.2
3.4
3.6
3.8
4.0
CT
CT + trastuzumab
Treatment

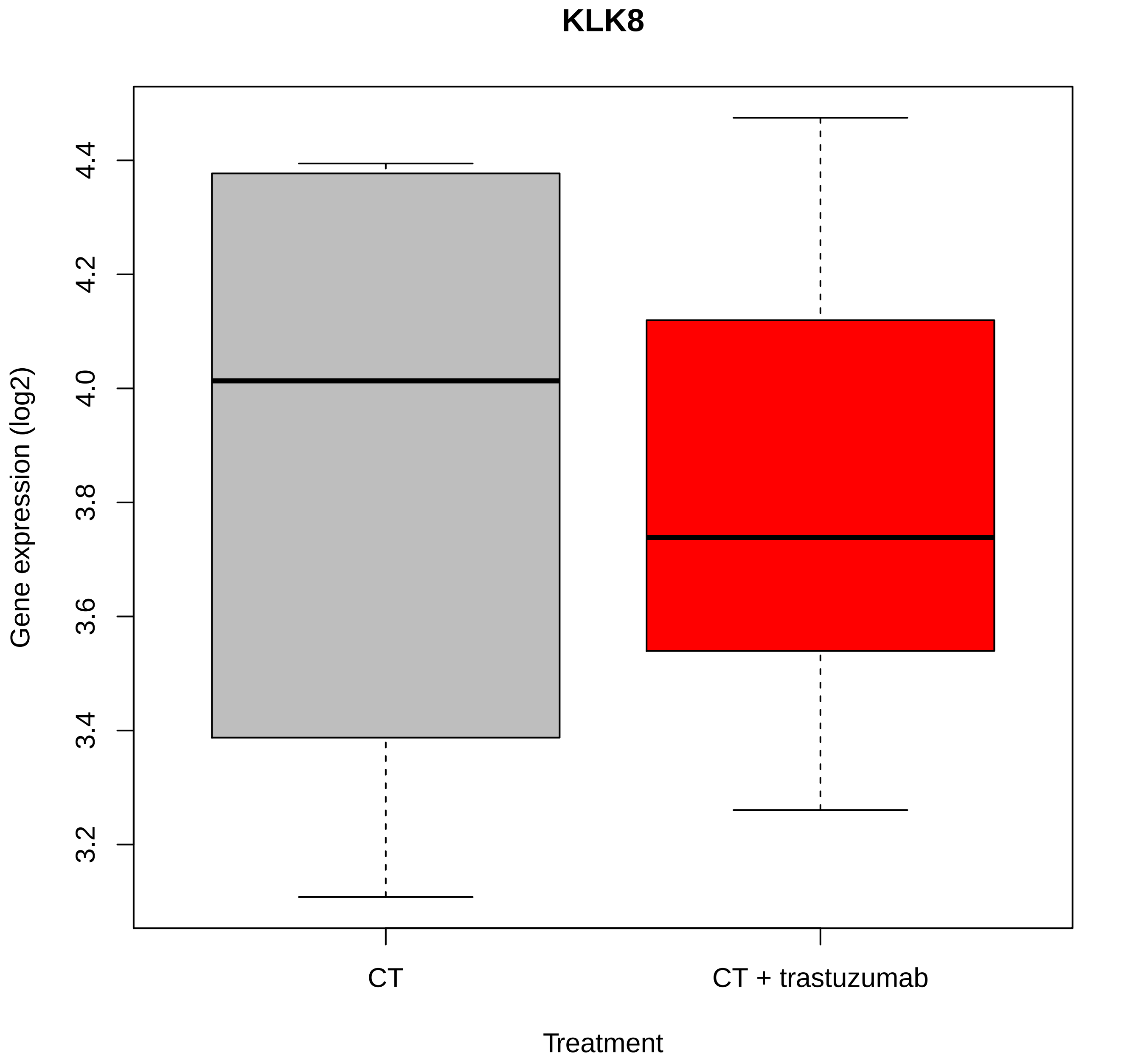

KLK8
Gene expression (log2)
3.2
3.4
3.6
3.8
4.0
4.2
4.4
CT
CT + trastuzumab
Treatment

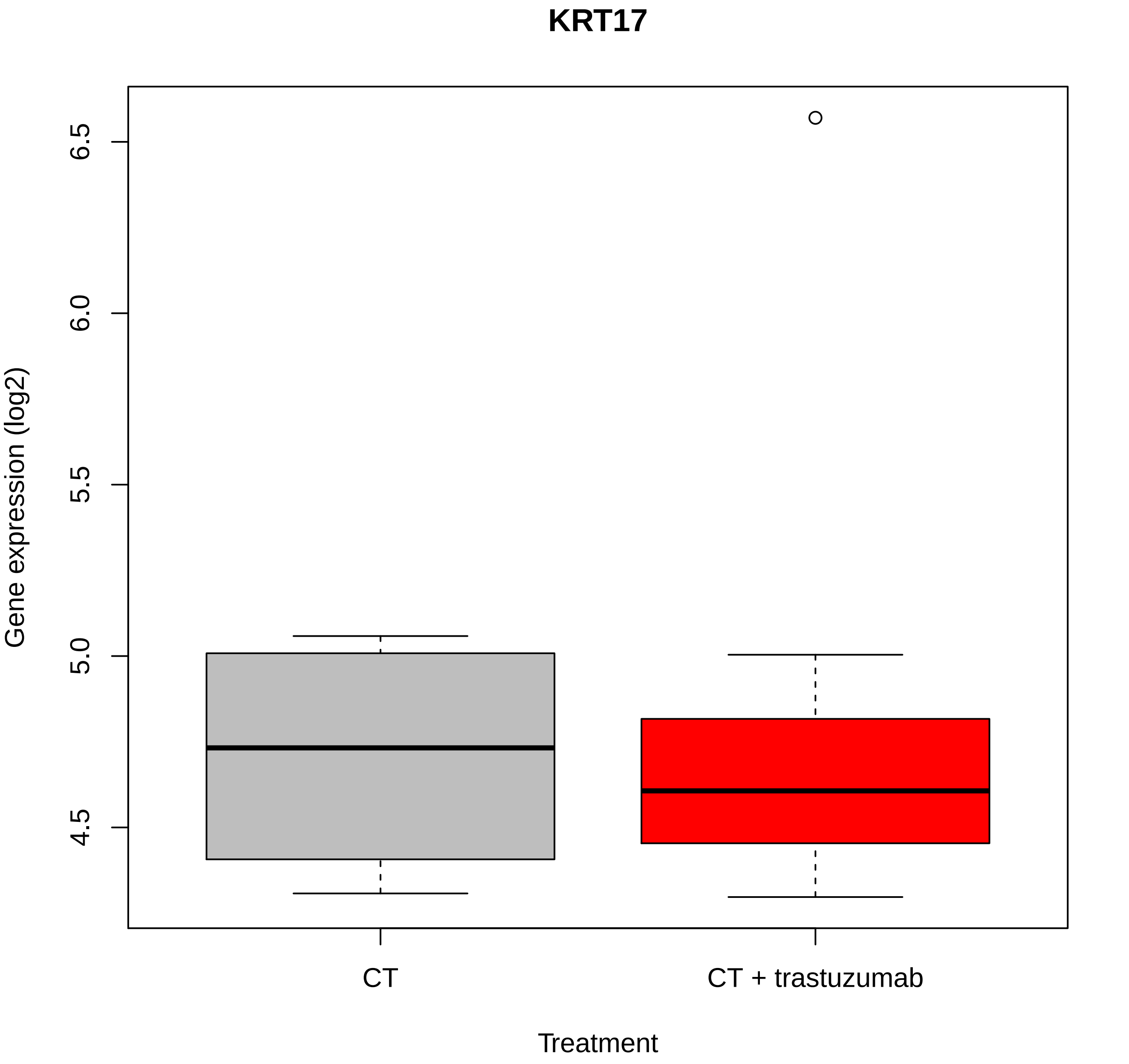
KRT17
Gene expression (log2)
6.5
6.0
5.5
5.0
4.5
CT
CT + trastuzumab
Treatment

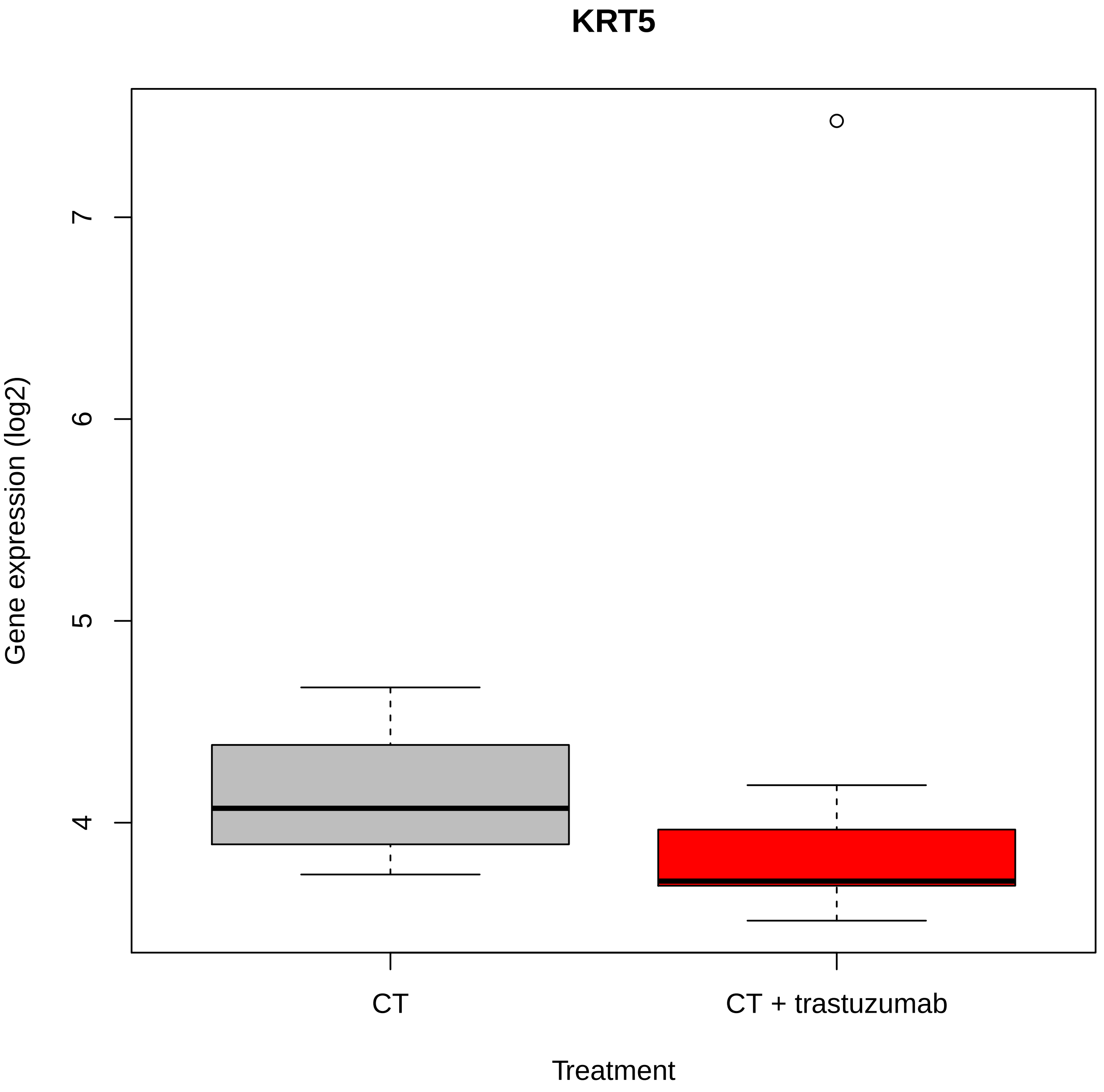
KRT5
Gene expression (log2)
7
6
5
4
CT
CT + trastuzumab
Treatment

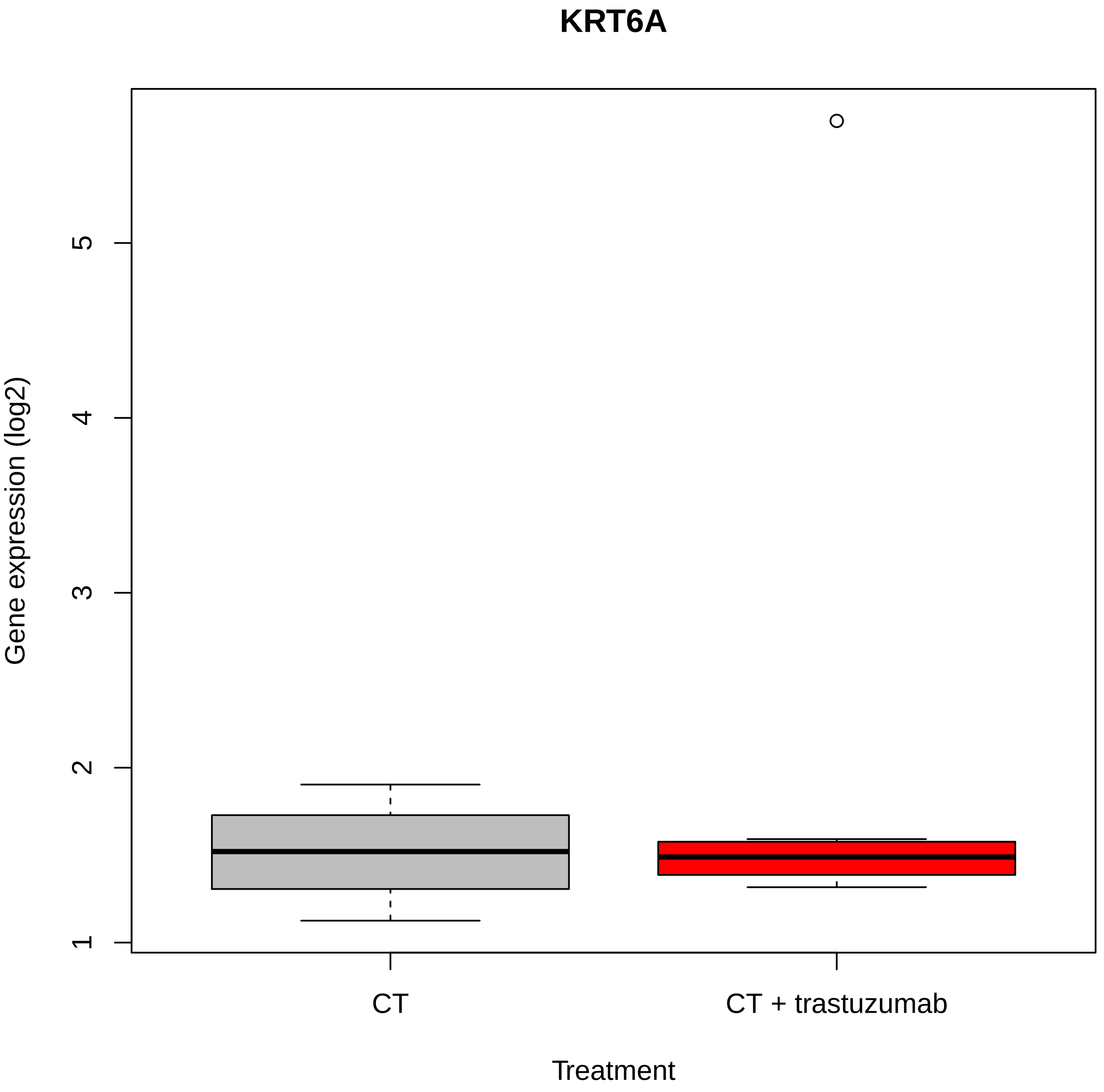
KRT6A
Gene expression (log2)
1
2
3
4
5
CT
CT + trastuzumab
Treatment

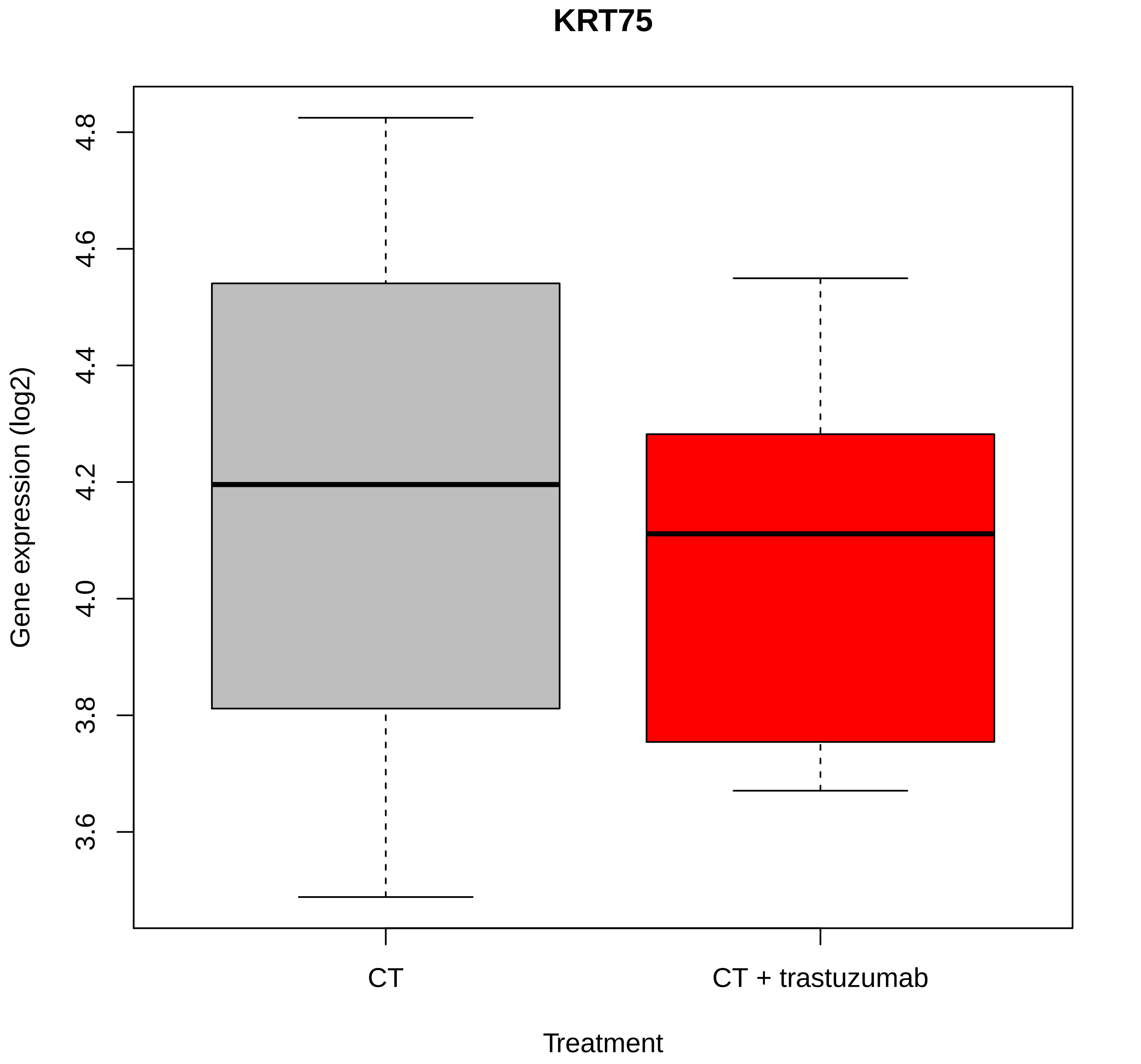
KRT75
4.8
4.6
4.4
4.2
4.0
3.8
3.6
Gene expression (log2)
CT
CT + trastuzumab
Treatment

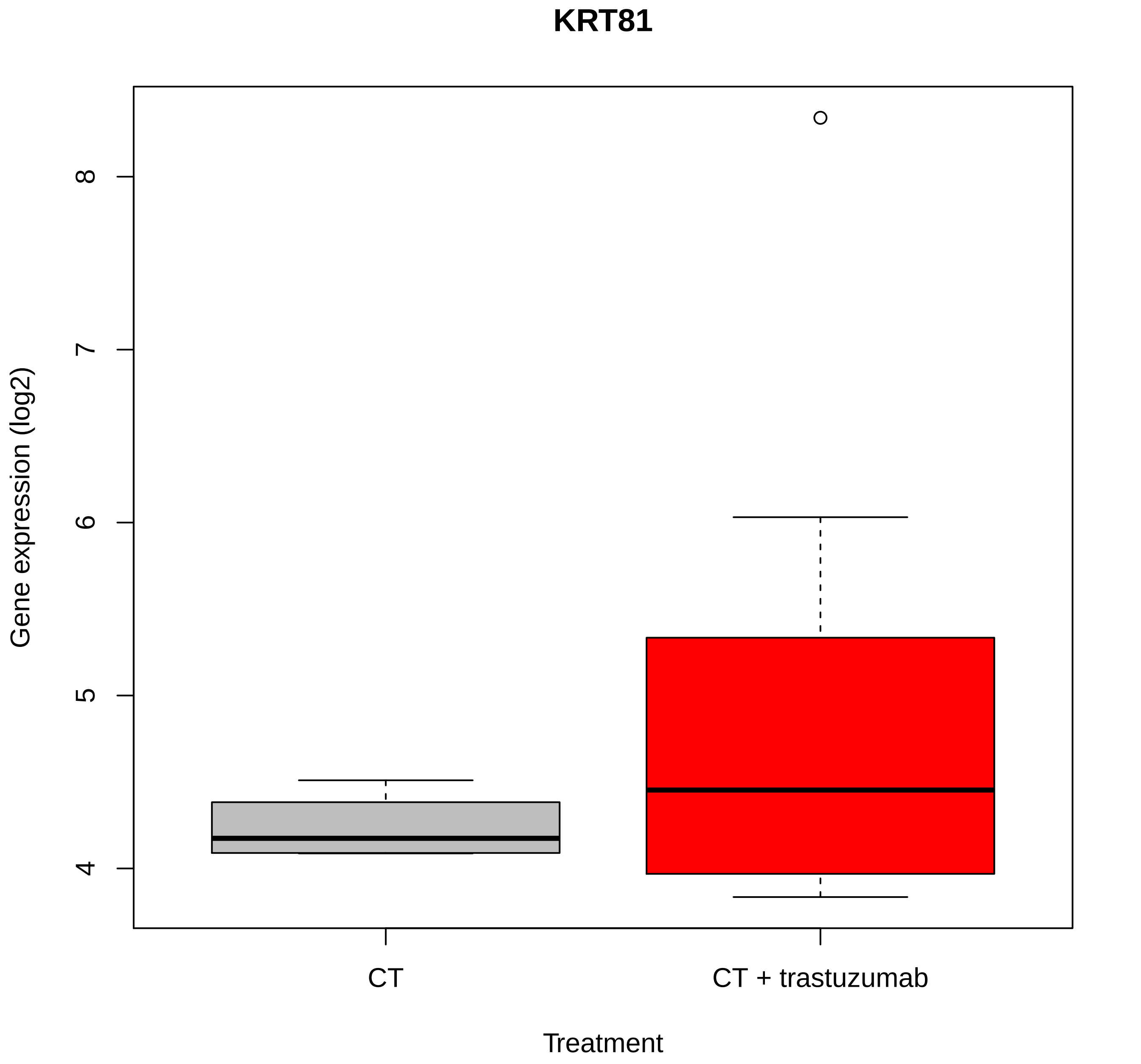
KRT81
Gene expression (log2)
4
5
6
7
8
CT
CT + trastuzumab
Treatment

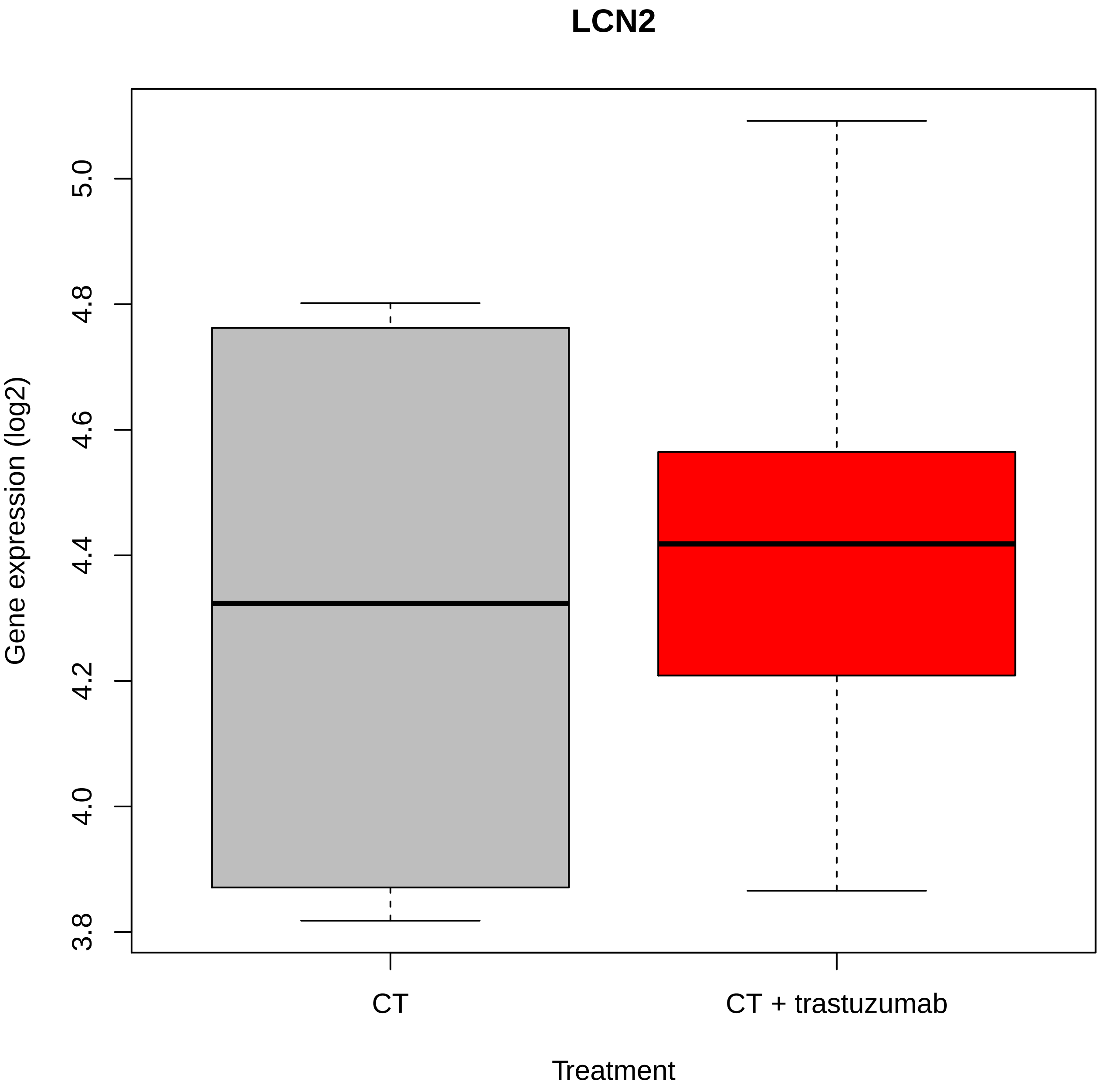
LCN2
Gene expression (log2)
3.8
4.0
4.2
4.4
4.6
4.8
5.0
CT
CT + trastuzumab
Treatment

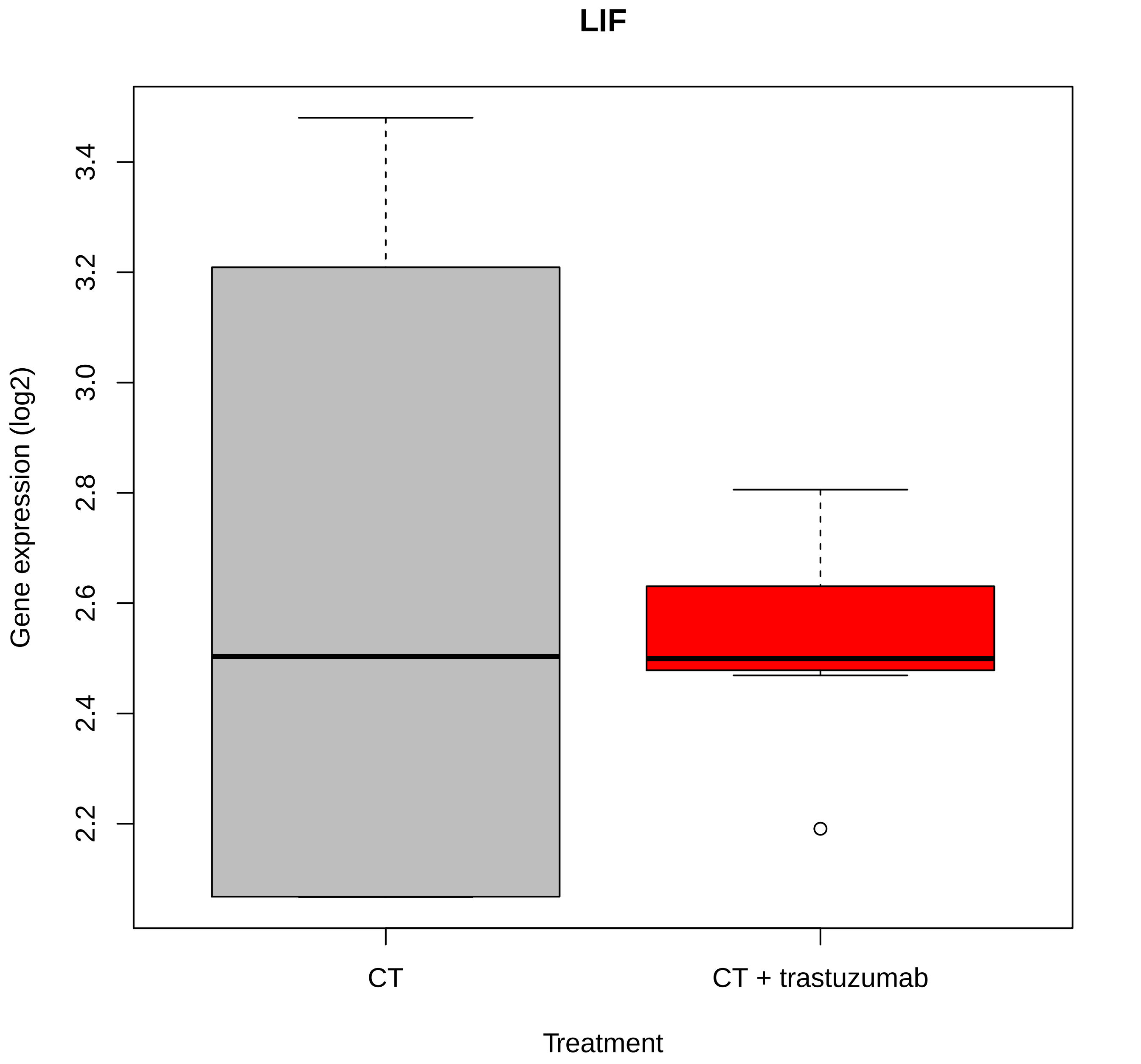

LIF
Gene expression (log2)
2.2
2.4
2.6
2.8
3.0
3.2
3.4
CT
CT + trastuzumab
Treatment

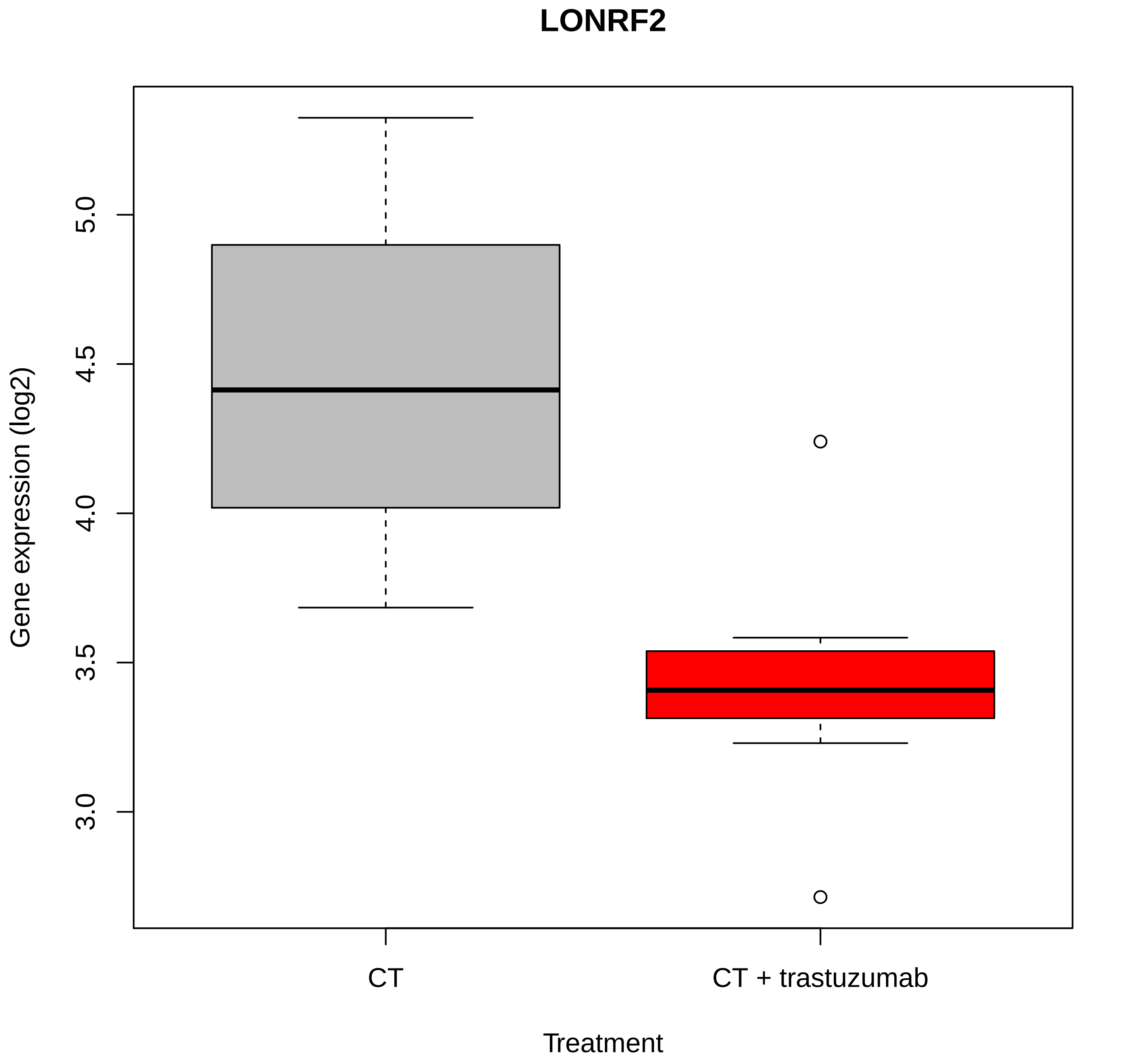
LONRF2
Gene expression (log2)
3.0
3.5
4.0
4.5
5.0
CT
CT + trastuzumab
Treatment

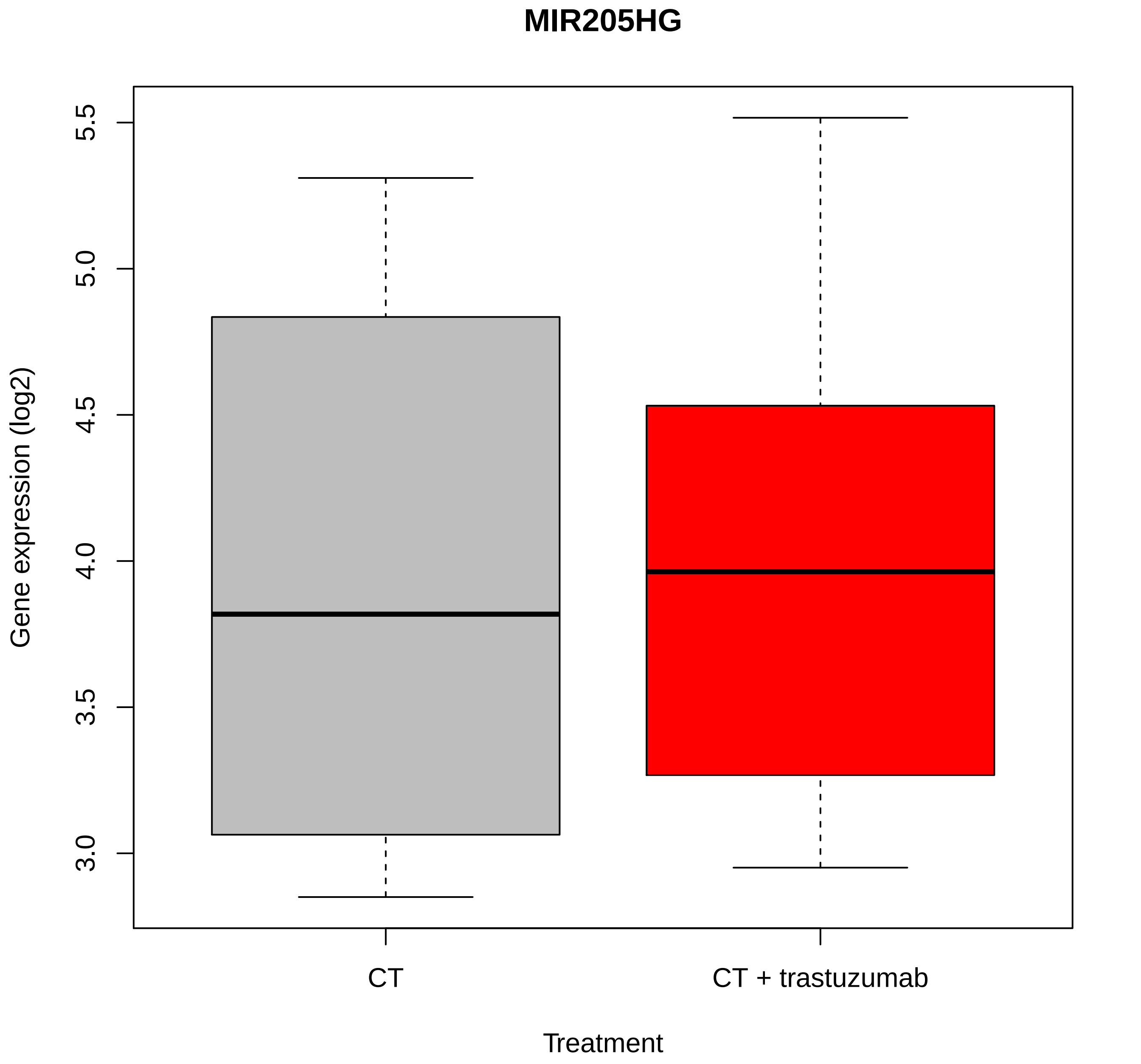
MIR205HG
Gene expression (log2)
3.0
3.5
4.0
4.5
5.0
5.5
CT
CT + trastuzumab
Treatment

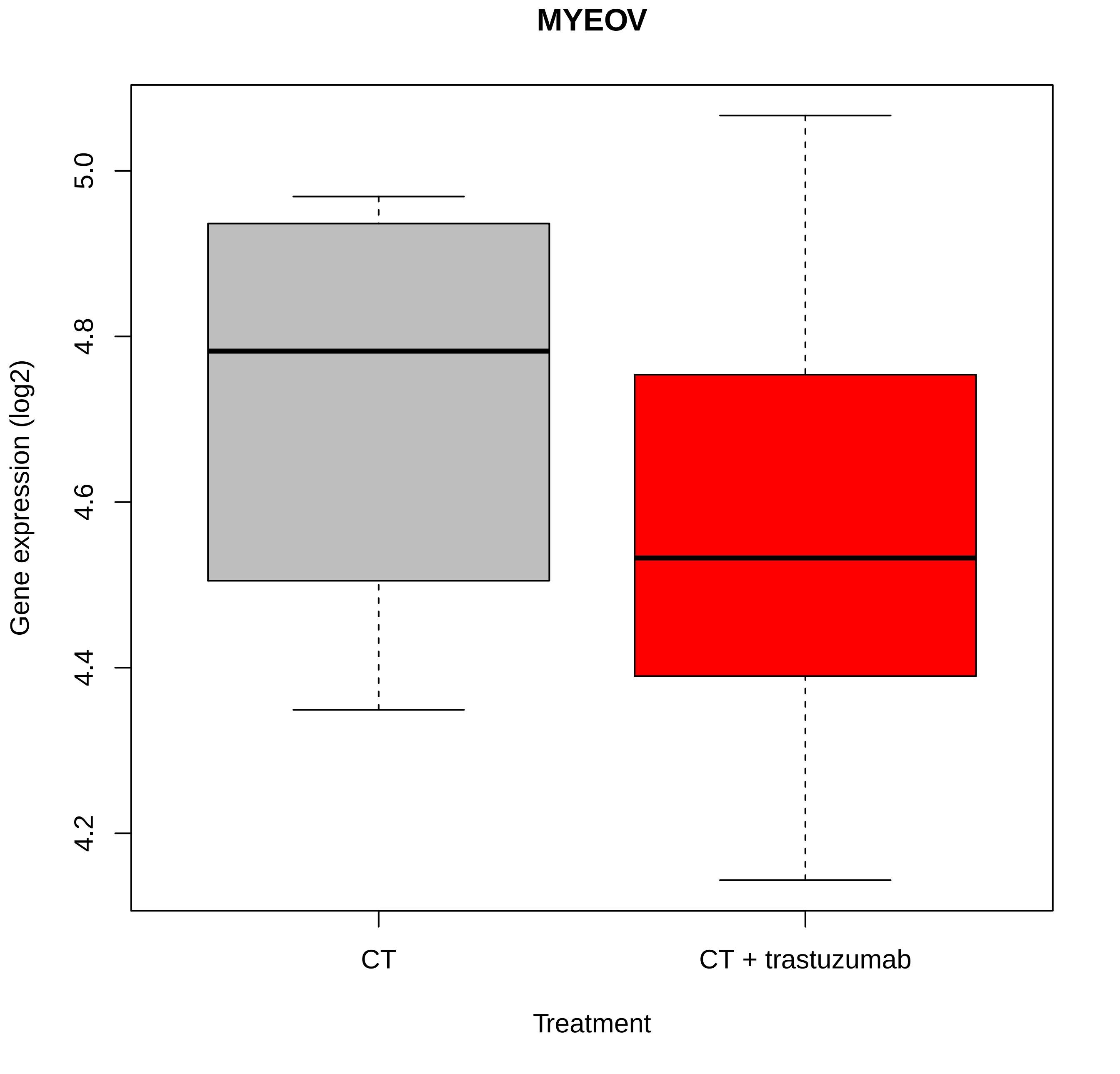

MYEOV
Gene expression (log2)
5.0
4.8
4.6
4.4
4.2
CT
CT + trastuzumab
Treatment

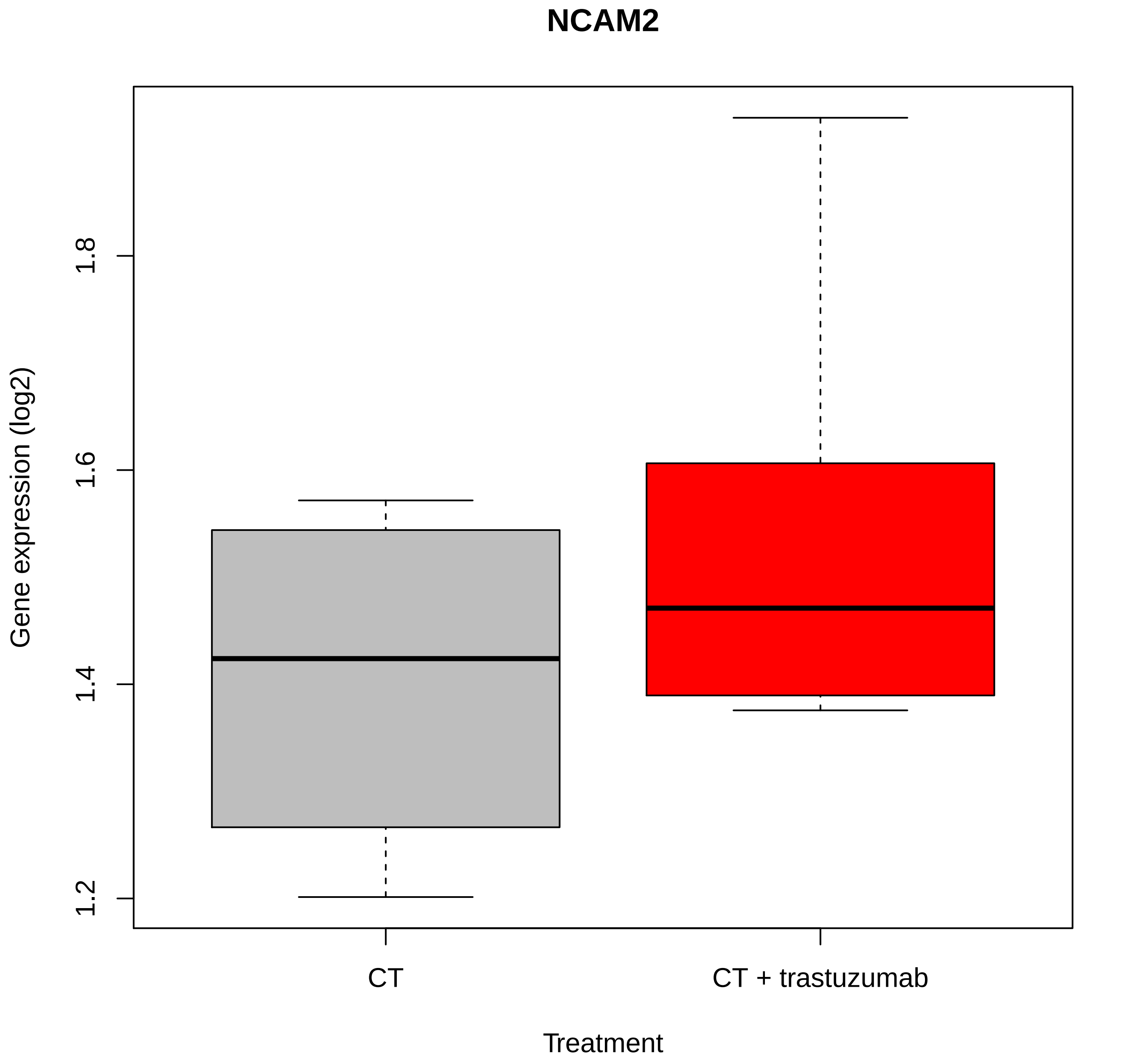
NCAM2
Gene expression (log2)
1.2
1.4
1.6
1.8
CT
CT + trastuzumab
Treatment

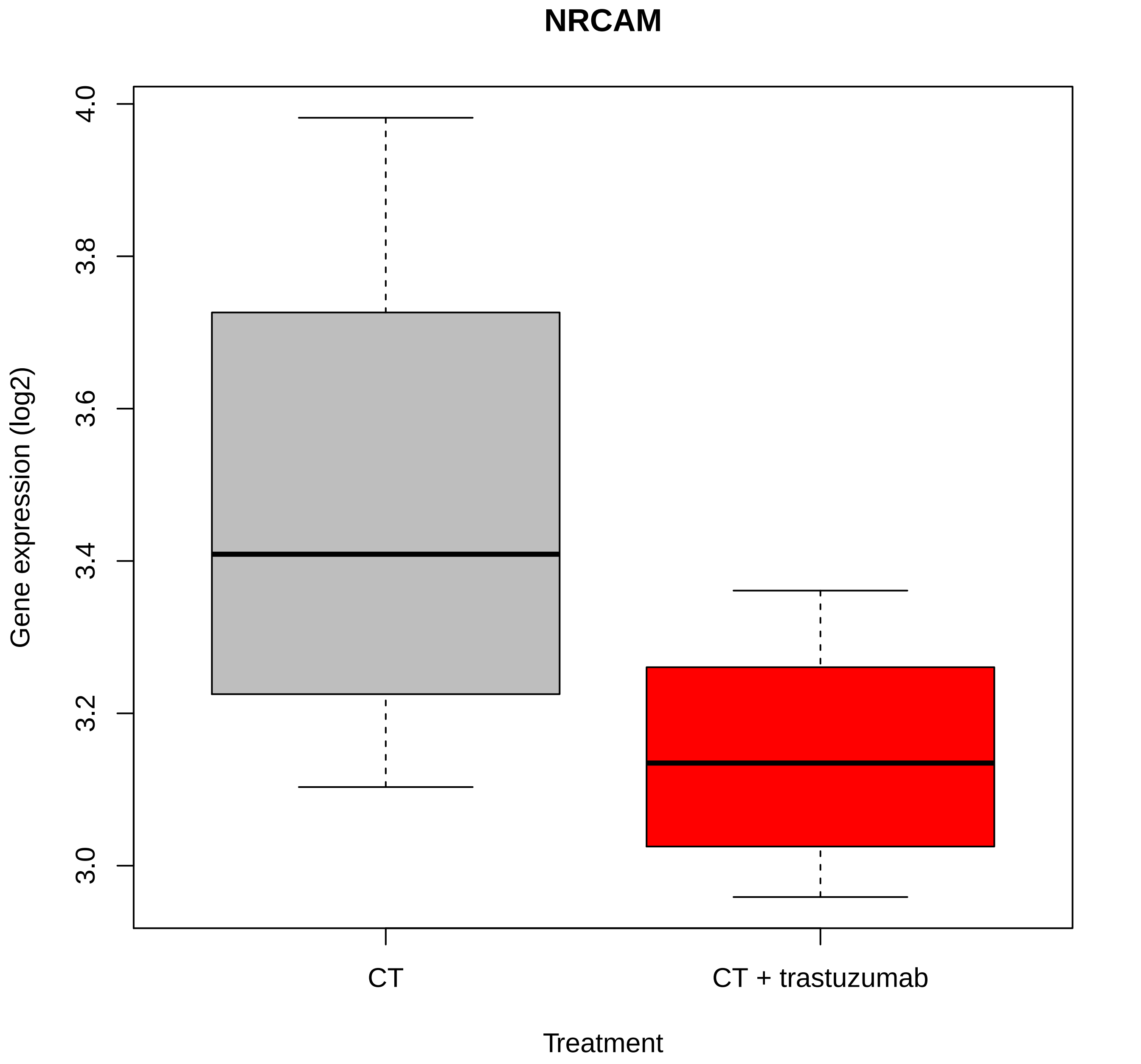

NRCAM
Gene expression (log2)
4.0
3.8
3.6
3.4
3.2
3.0
CT
CT + trastuzumab
Treatment

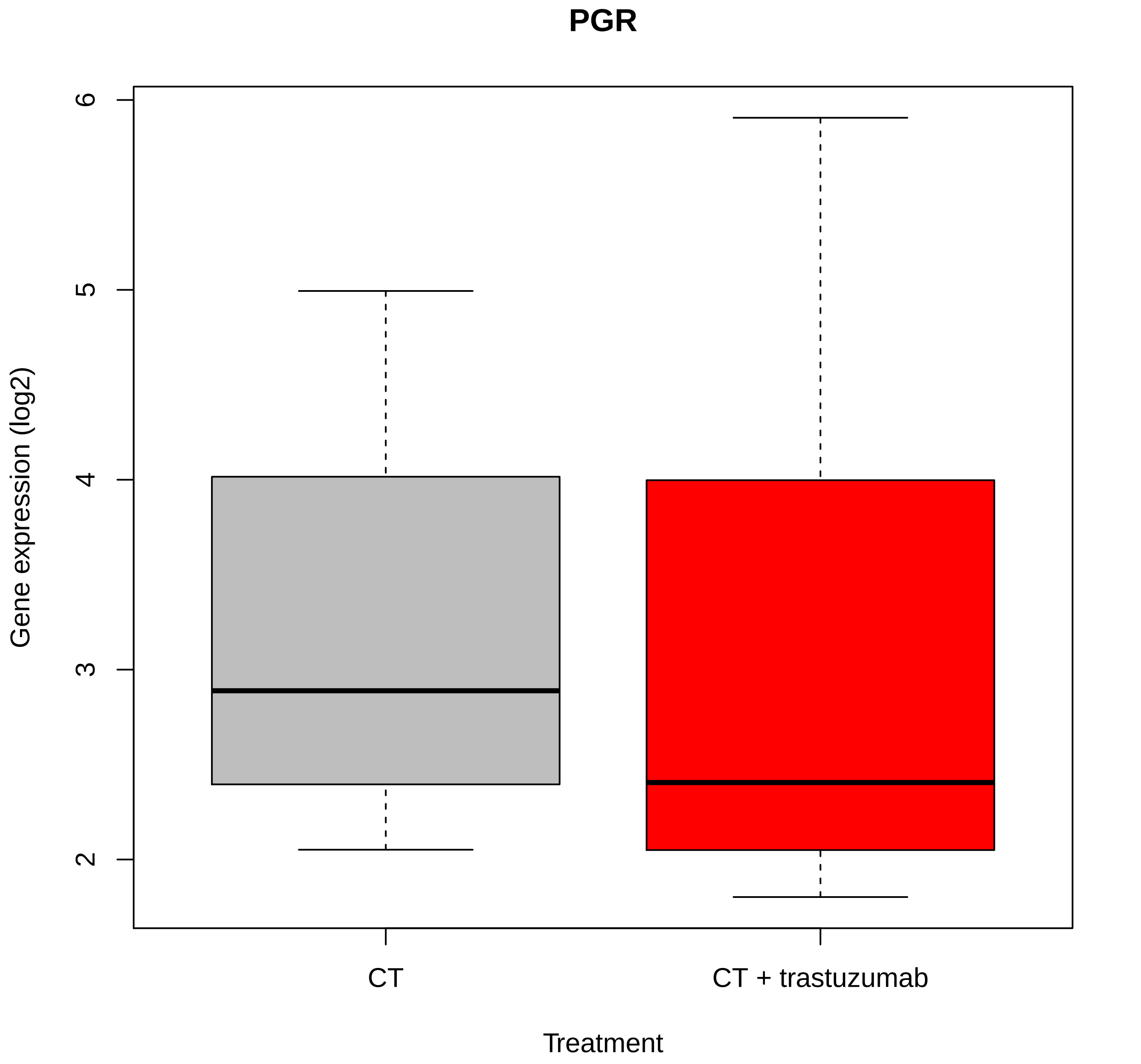
PGR
Gene expression (log2)
2
3
4
5
6
CT
CT + trastuzumab
Treatment

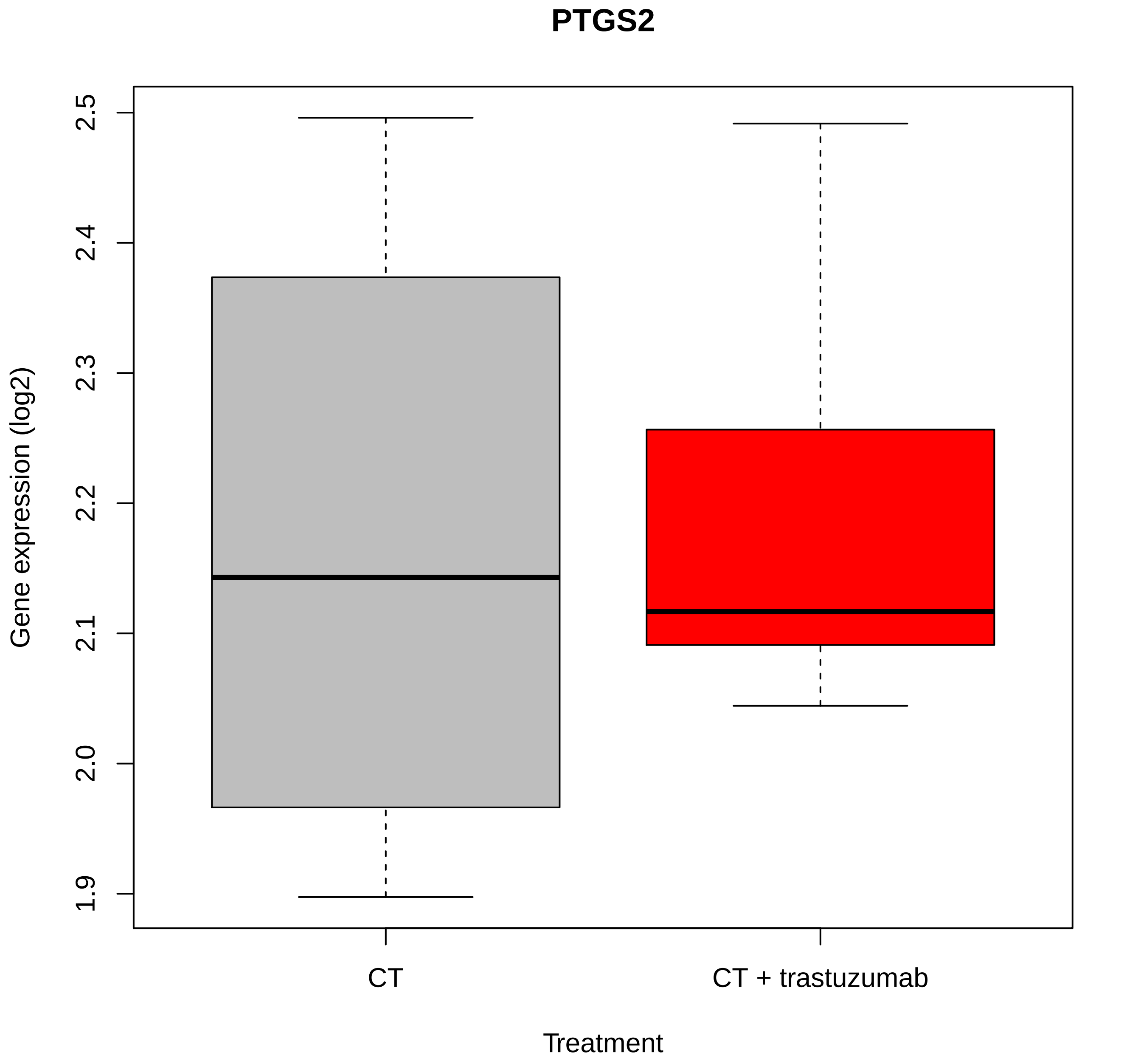

PTGS2
Gene expression (log2)
2.5
2.4
2.3
2.2
2.1
2.0
1.9
CT
CT + trastuzumab
Treatment

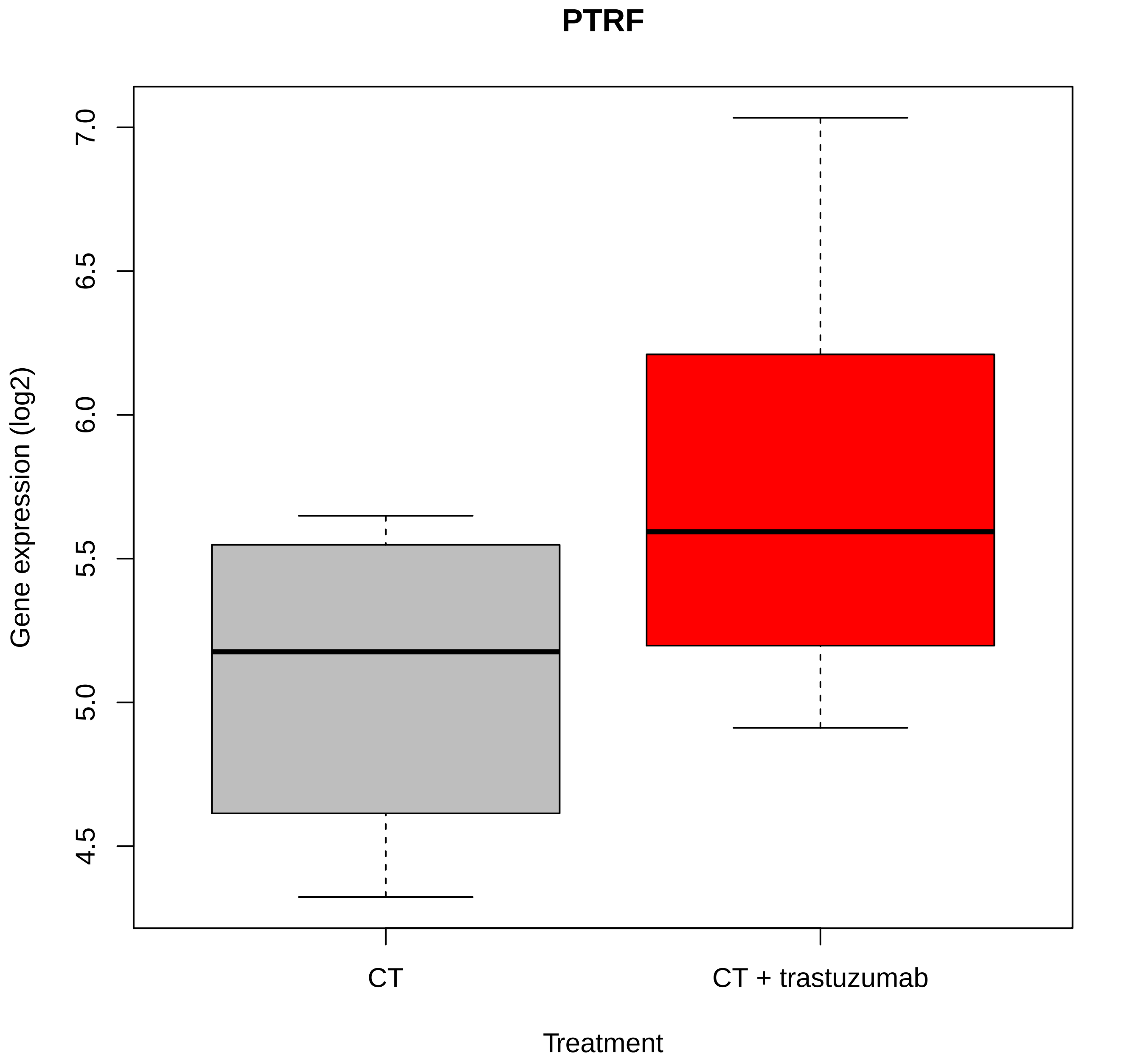
PTRF
Gene expression (log2)
7.0
6.5
6.0
5.5
5.0
4.5
CT
CT + trastuzumab
Treatment

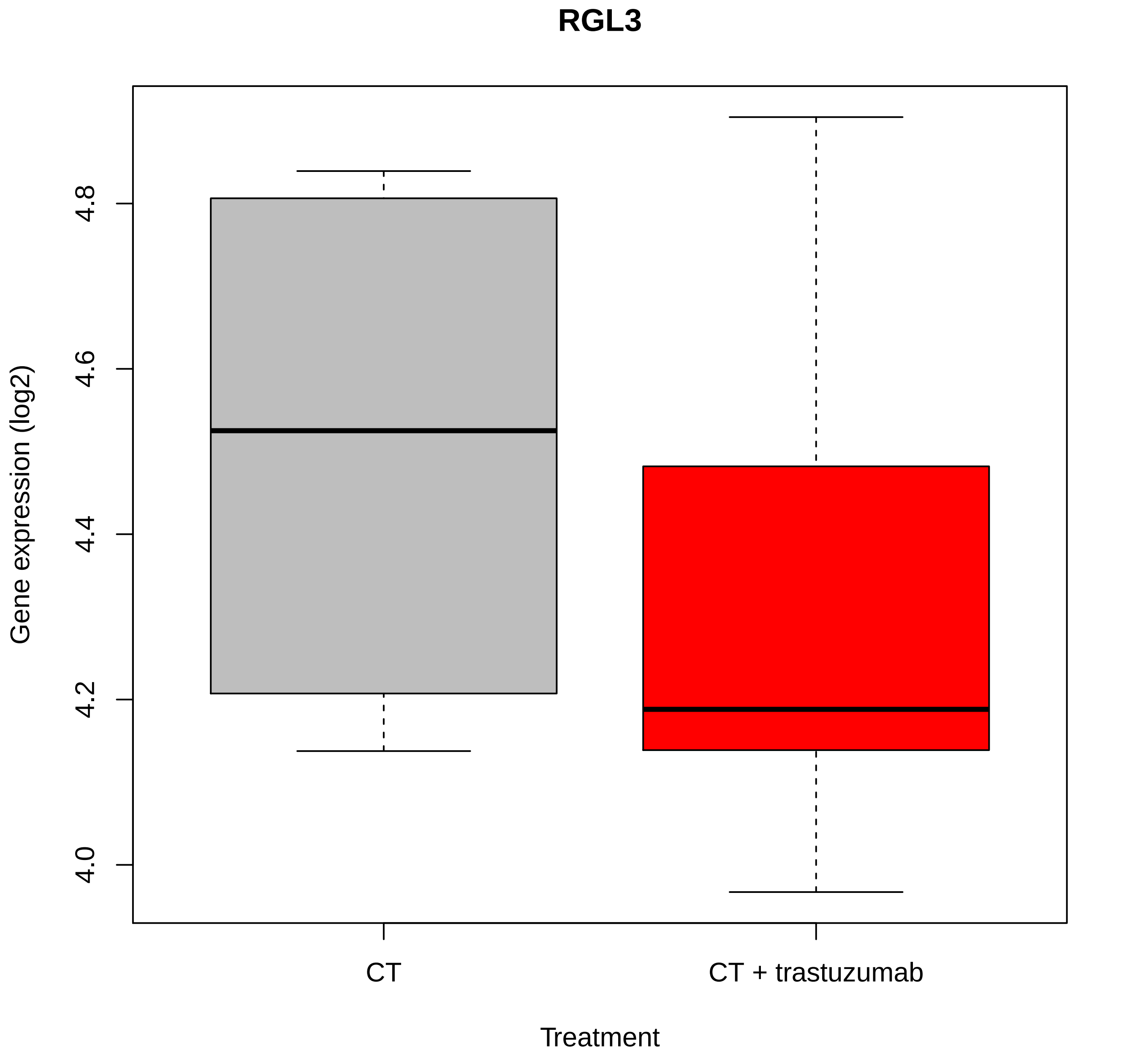
RGL3
Gene expression (log2)
4.0
4.2
4.4
4.6
4.8
CT
CT + trastuzumab
Treatment

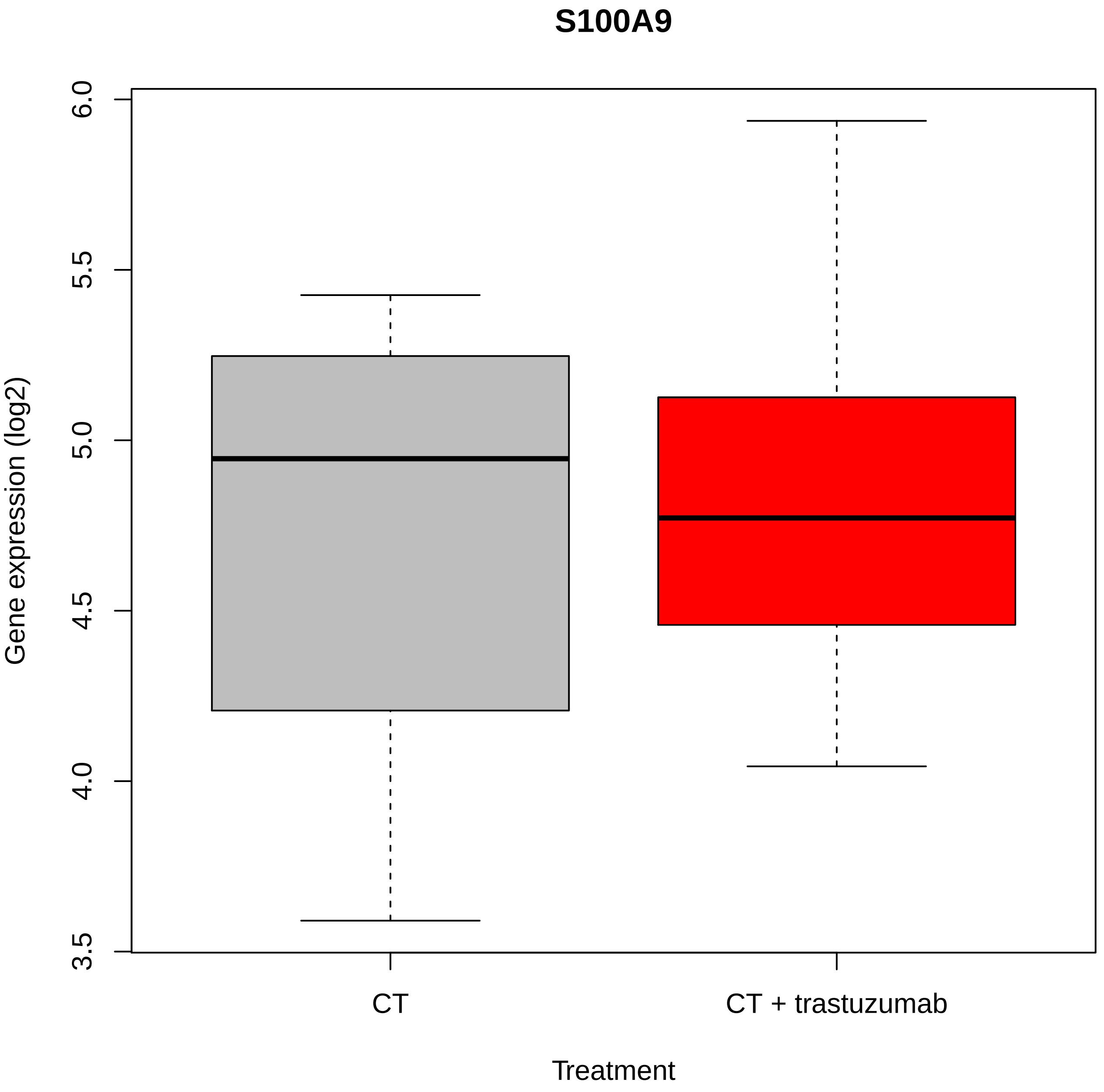

S100A9
Gene expression (log2)
3.5
4.0
4.5
5.0
5.5
6.0
CT
CT + trastuzumab
Treatment

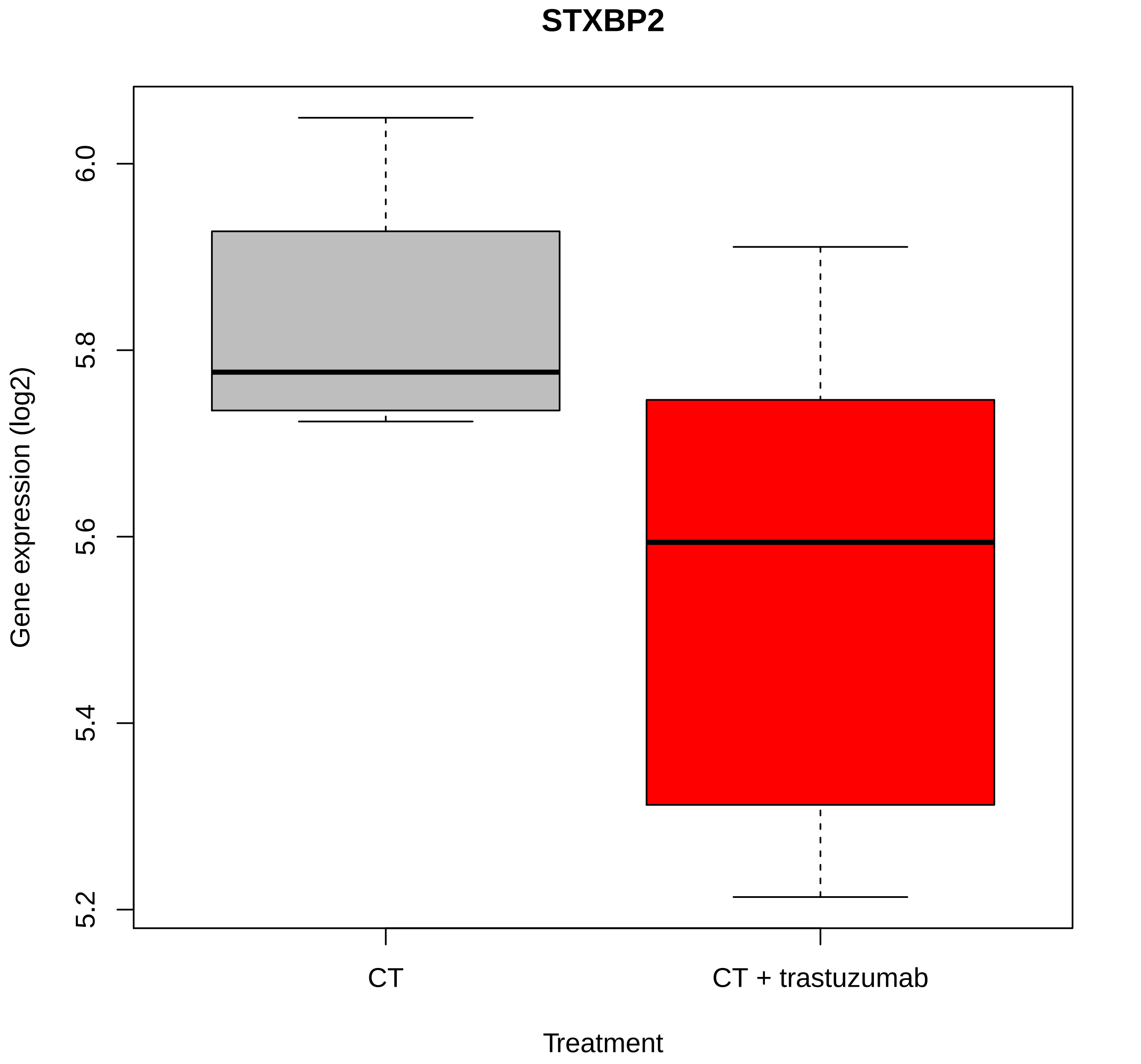
STXBP2
Gene expression (log2)
6.0
5.8
5.6
5.4
5.2
CT
CT + trastuzumab
Treatment

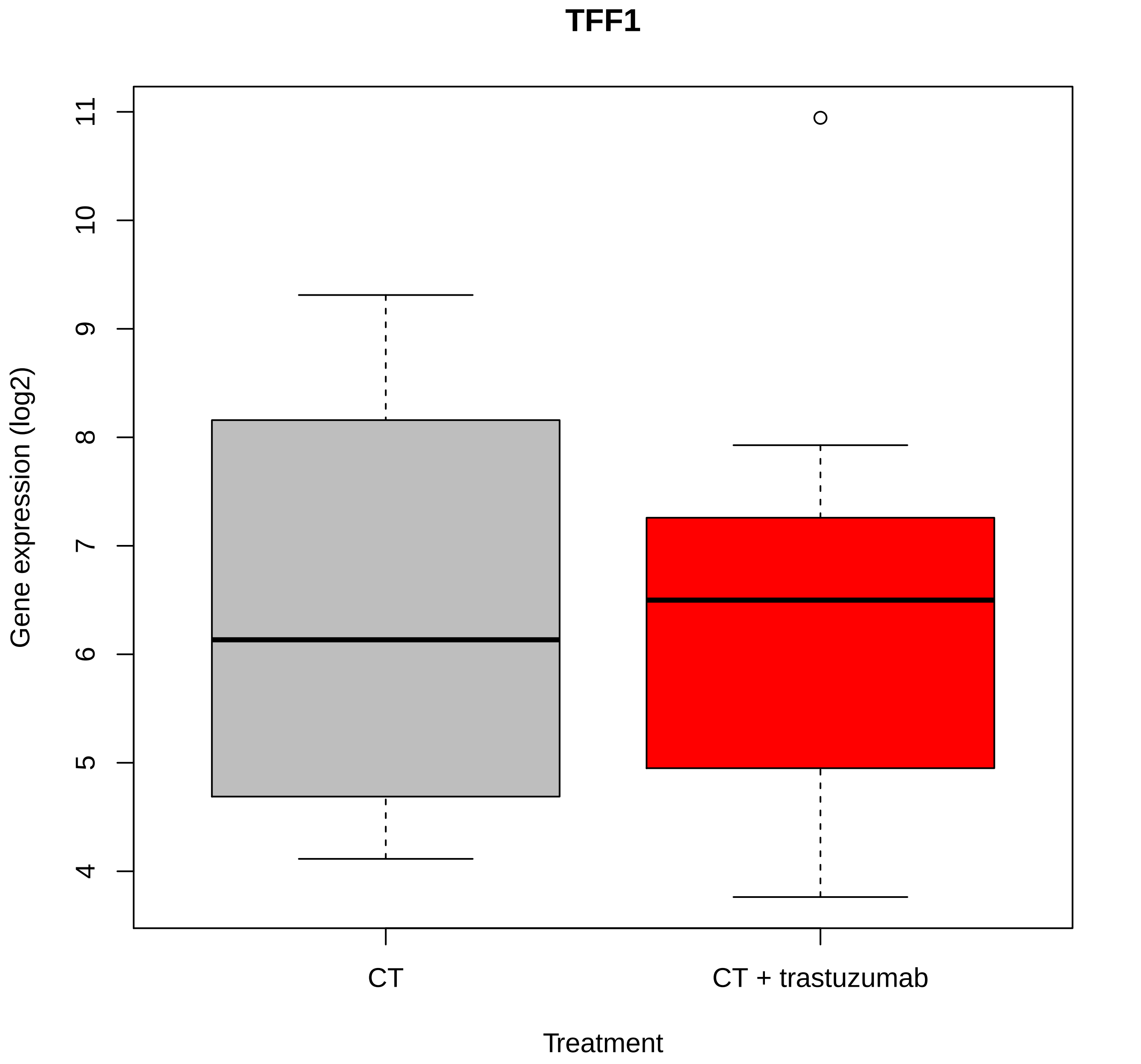
TFF1
Gene expression (log2)
4
5
6
7
8
9
10
11
CT
CT + trastuzumab
Treatment

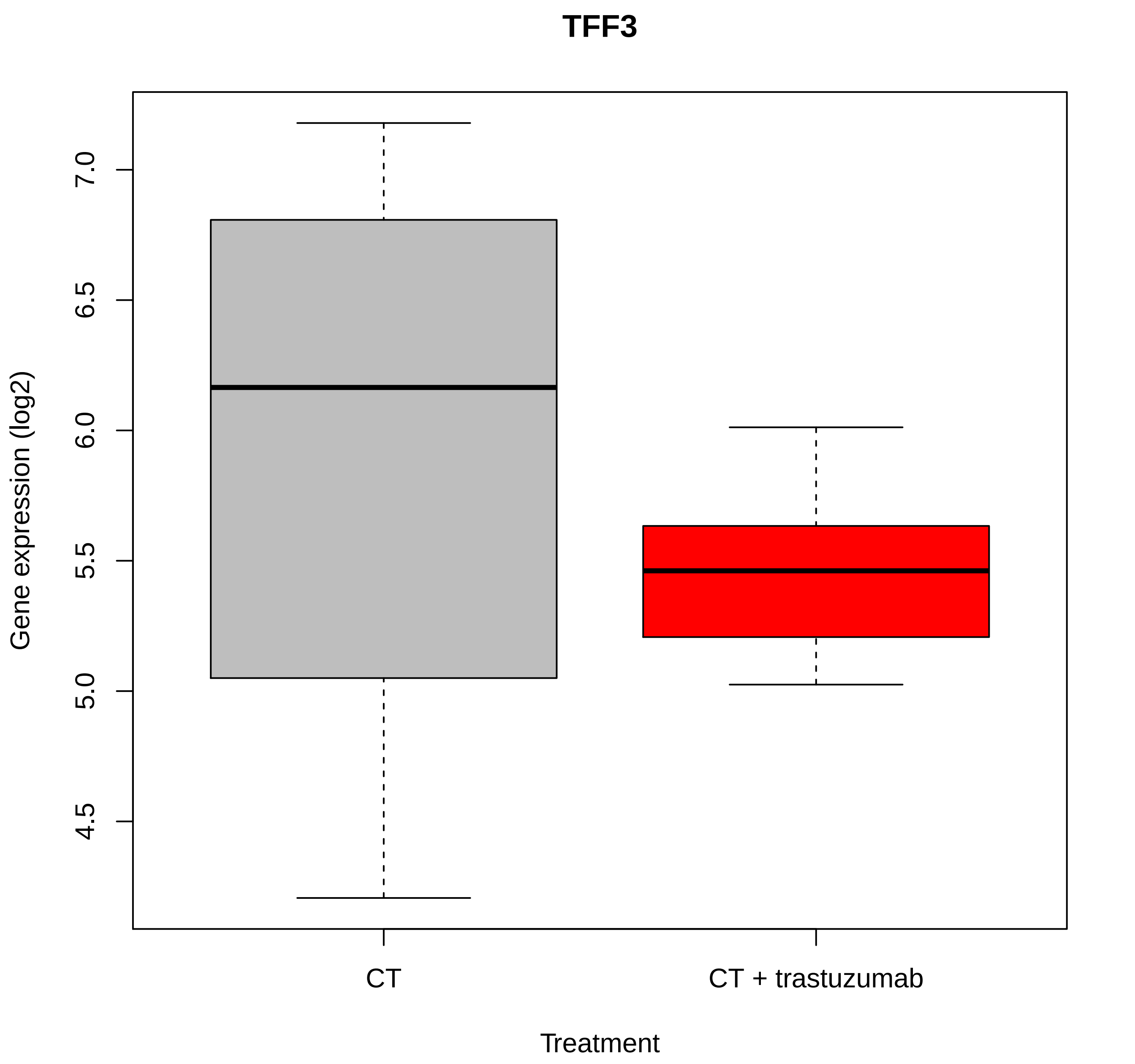

TFF3
Gene expression (log2)
4.5
5.0
5.5
6.0
6.5
7.0
CT
CT + trastuzumab
Treatment

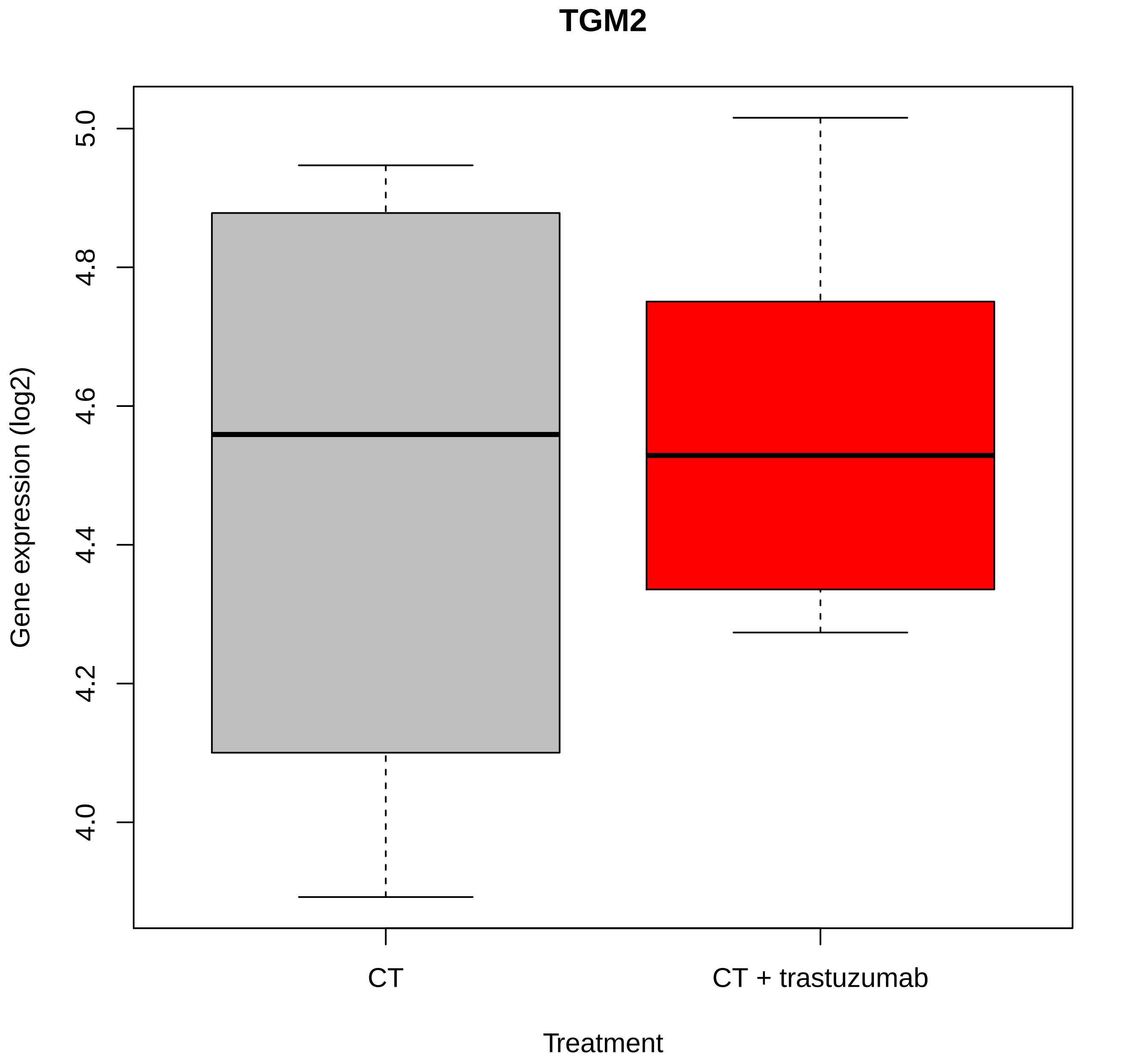
TGM2
Gene expression (log2)
5.0
4.8
4.6
4.4
4.2
4.0
CT
CT + trastuzumab
Treatment

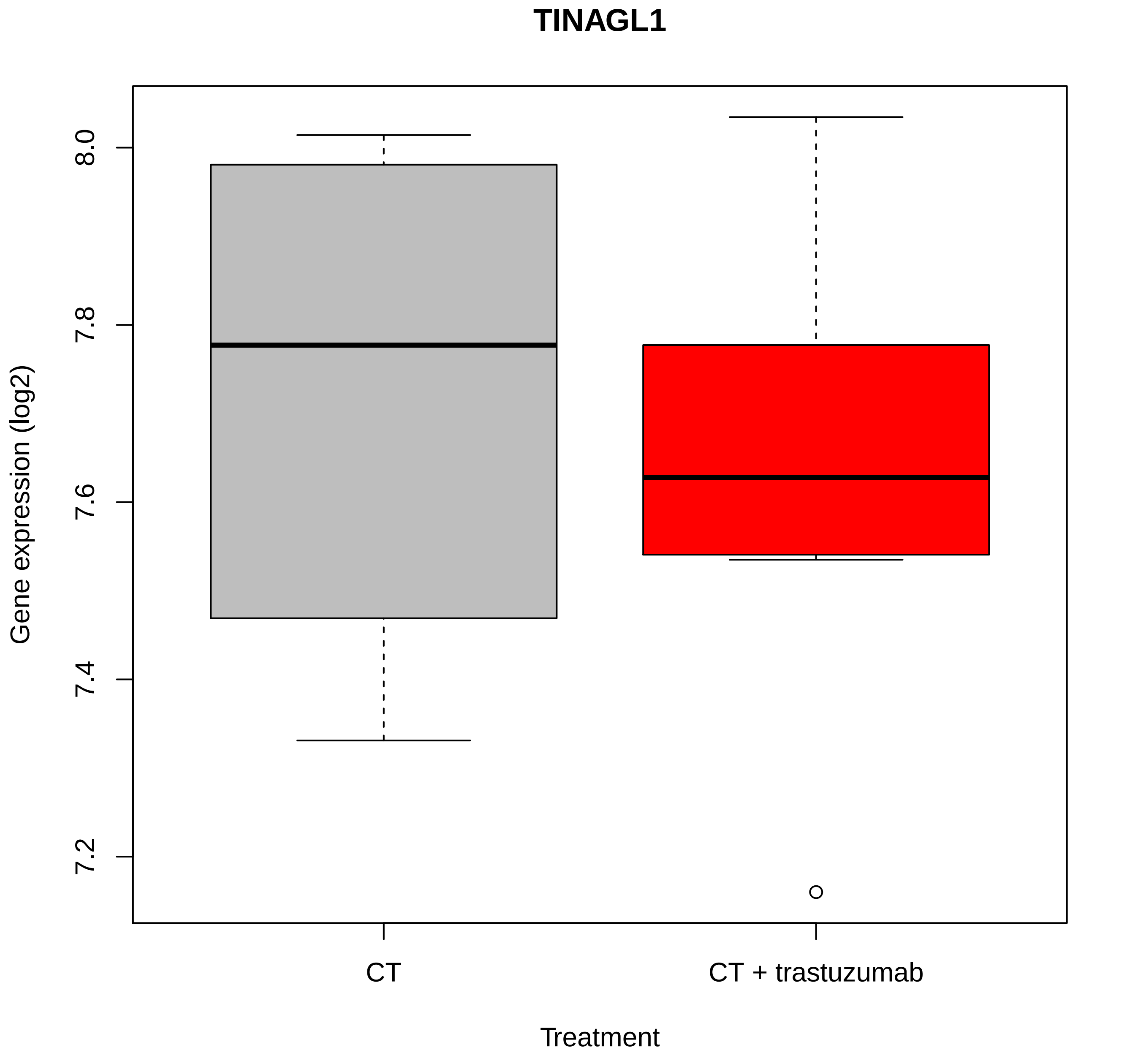
TINAGL1
Gene expression (log2)
7.2
7.4
7.6
7.8
8.0
CT
CT + trastuzumab
Treatment
